# Modulation of the substrate preference of a MYST acetyltransferase by a scaffold protein

**DOI:** 10.1016/j.jbc.2025.108262

**Published:** 2025-02-03

**Authors:** Raghuvir N. Sengupta, Oleg Brodsky, Patrick Bingham, Wade C. Diehl, RoseAnn Ferre, Samantha E. Greasley, Eric Johnson, Michelle Kraus, Whitney Lieberman, Jordan L. Meier, Thomas A. Paul, Karen A. Maegley

**Affiliations:** 1 Oncology Research and Development, Pfizer, La Jolla, California, USA; 2 Medicine Design, Pfizer Research and Development, Pfizer, La Jolla, California, USA; 3 Chemical Biology Laboratory, National Cancer Institute, Frederick, Maryland, USA

**Keywords:** acetylation, histone acetyltransferases, multiprotein complexes, specificity

## Abstract

The MYST family of lysine acetyltransferases are transcriptional regulators often dysregulated in cancer. In cells, MYST members form distinct multiprotein complexes that guide their histone substrate specificity, but how this selectivity is conferred is not fully understood. Here we interrogate a complex-mediated change in the substrate preference of the MYST member KAT6A, a target for cancer therapeutics. KAT6A forms a 4-protein complex with BRPF1, ING4/5, and MEAF6 to acetylate H3K23. However, additional substrates (H3K9, H3K14, and H3K27) have been proposed, and whether these residues are modified by KAT6A is unclear. We determined the histone substrate specificity of uncomplexed forms of KAT6A, including full-length KAT6A (KAT6A^FL^) and the isolated acetyltransferase (MYST) domain, and the KAT6A^FL^ 4-protein complex (KAT6A^FL^ 4-plex). We show that the MYST domain and KAT6A^FL^ preferentially acetylate H3K14, with this selectivity linked to a glycine pair preceding K14. A structure of the MYST domain bound to an H3K14-CoA bisubstrate inhibitor is consistent with a model in which the small size and flexibility of this glycine pair facilitate K14 acetylation. Notably, when KAT6A^FL^ assembles into the 4-plex, H3K23 emerges as the favored substrate, with favorable recognition of an alanine-threonine pair before K23. These changes are mediated by BRPF1 and steady-state assays with H3 peptides indicate that this scaffold protein can alter the substrate preference of KAT6A^FL^ by ≈10^3^-fold. Such context-dependent specificity illustrates how the functional properties of MYST members can be modulated by associated proteins and underscores the importance of characterizing these enzymes in their free and complex forms.

Posttranslational modification of chromatin is critical for eukaryotic gene regulation, and the enzymes that mediate these reactions are often dysregulated in cancer (1). An emerging paradigm in cancer therapy is the use of small molecule inhibitors against the catalysts that install (writers) or remove (erasers) these modifications, with multiple FDA-approved therapeutics that target DNA methyltransferases, lysine methyltransferases, and lysine deacetylases (2, 3). In addition, recent advances have led to the development of small molecule inhibitors against lysine acetyltransferases (4, 5), which are commonly involved in cancer cell proliferation. For example, we recently reported the small molecule CTx-648 (PF-9363), which targets the lysine acetyltransferase KAT6A and its paralog KAT6B (6).

Mutation, gene amplification, and gene fusion of *KAT6A* and/or *KAT6B* are associated with oncogenesis (7, 8, 9), and cancer cell lines that overexpress KAT6A are dependent on this enzyme, strongly suggesting that it is a therapeutic vulnerability (6). Indeed, PF-9363, a potent and selective acetyl-CoA competitive inhibitor against KAT6A/B^1^, exhibits robust anti-tumor activity in estrogen receptor-positive (ER+) breast cancer models, including tumors resistant to endocrine therapy (6), and structurally related inhibitor shows clinical efficacy in a Phase 1 trial in advanced or metastatic solid tumors (10).

KAT6A and KAT6B are members of the MYST family of acetyltransferases, a group of transcriptional regulators implicated in cellular proliferation and development (11). The hallmark of this family, which includes KAT5, KAT6A, KAT6B, KAT7, and KAT8 (also known as Tip60, MOZ, MORF, HBO1, and MOF, respectively), is the MYST acetyltransferase domain (herein referred to as the MYST domain) that employs acetyl-CoA to catalyze acetylation of lysine residues within the histone tails of the nucleosome (11). However, despite substantial sequence and structural conservation of this catalytic domain (12, 13), MYST members exhibit distinct histone substrate specificities in cells: H2A.ZK7 by KAT5 (14); H3K23 by KAT6A and KAT6B (6, 15); H3K14, H4K5, H4K8, and H4K12 by KAT7 (16, 17, 18, 19, 20); and H4K5, H4K8, and H4K16 by KAT8 (21, 22, 23, 24, 25). This individualized substrate specificity presumably arises from the distinct multiprotein complexes formed by each MYST family enzyme in cells (Table S1 and references therein), but the mechanisms underlying this modulation are not fully understood. Given the frequent perturbation of MYST family acetyltransferases in cancer (5, 26) and the recent interest in their therapeutic targeting (4, 5), delineating these mechanisms is critical for our broad understanding of transcriptional regulation and the pharmacology of drug candidates.

KAT6A/B forms a 4-protein complex (4-plex) with BRPF1 and two small accessory proteins, ING4/5 and MEAF6 (Fig. 1) (8, 9). Cellular data strongly suggest that this heterotetrameric complex directs H3K23 acetylation (6, 15) and *in vitro* studies with the recombinant KAT6A and KAT6B 4-plexes provide evidence for robust acetyltransferase activity towards this lysine residue (6, 15, 27). However, *in vitro* data for monomeric forms of KAT6A or KAT6B that retain the MYST domain implicate H3K14 and H3K9 as substrates (28, 29, 30). In cell cultures, when KAT6A expression is disrupted, reduced acetylation of H3K9 (31, 32, 33, 34, 35, 36), H3K14 (6, 34, 35), H3K23 (6, 15, 35), and H3K27 (33) has been observed. Whether each of these lysine residues are *bona fide* substrates for KAT6A/B alone or as part of the 4-plex is not clear.

**Figure 1:**
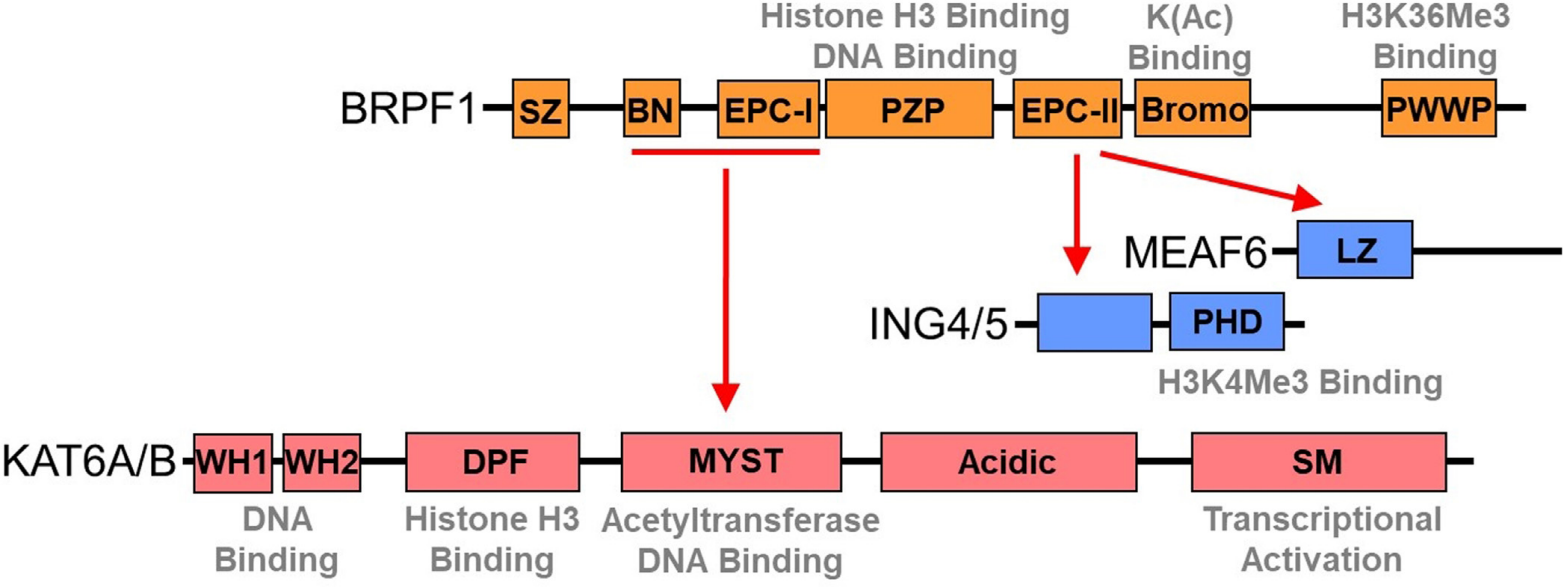
**Domain representation of KAT6A/B complexed with BRPF1 (bromodomain and PHD finger-containing protein 1), ING4/5 (inhibitor of growth 4 or 5), and MEAF6 (mammalian Esa1-associated factor 6), adapted from** (15)**.** The *redarrows* correspond to regions required for association between proteins (29). The *gray* text refers to the reported functional roles of a given domain within each protein (see Table S2 and references therein). Regions within BRPF1 include Sfp1-like zinc finger motif (SZ), BRPF-specific N-terminal region (BN), Enhancer of Polycomb-like motifs I and II (EPC-I and EPC-II, respectively), a PHD-zinc knuckle-PHD (PZP) domain, a bromodomain (Bromo), and a Pro-Trp-Trp-Pro-containing domain (PWWP). Regions within KAT6A/B include winged helix domains 1 and 2 (WH1 and WH2, respectively), a double PHD finger (DPF), the MYST acetyltransferase domain, an acidic domain, and a serine/methionine-rich domain (SM). ING4/5 contains a PHD finger while MEAF6 includes a leucine zipper (LZ) motif.

Here we address the ambiguity surrounding KAT6A/B substrate selection by determining the substrate preference of the KAT6A MYST domain, full-length KAT6A (herein referred to as KAT6A^FL^), and the KAT6A^FL^ 4-plex. Our results demonstrate that uncomplexed forms of KAT6A preferentially modify H3K14 over all other H3 lysine residues, in contrast to the KAT6A^FL^ 4-plex which favors acetylation of H3K23. We provide direct evidence that this change in substrate specificity is mediated by the scaffold protein BRPF1, which alters the recognition of H3K23 by KAT6A^FL^. This approach illustrates how investigating MYST enzymes in their free and complex forms can validate potential cellular substrates, which is of value in drug development. More broadly, this type of interrogation can serve as a starting point to uncover and characterize strategies employed by Nature to modulate the functional properties of these enzymes.

## Results

### Preferential acetylation of H3K14 by the KAT6A MYST domain

**Figure 2:**
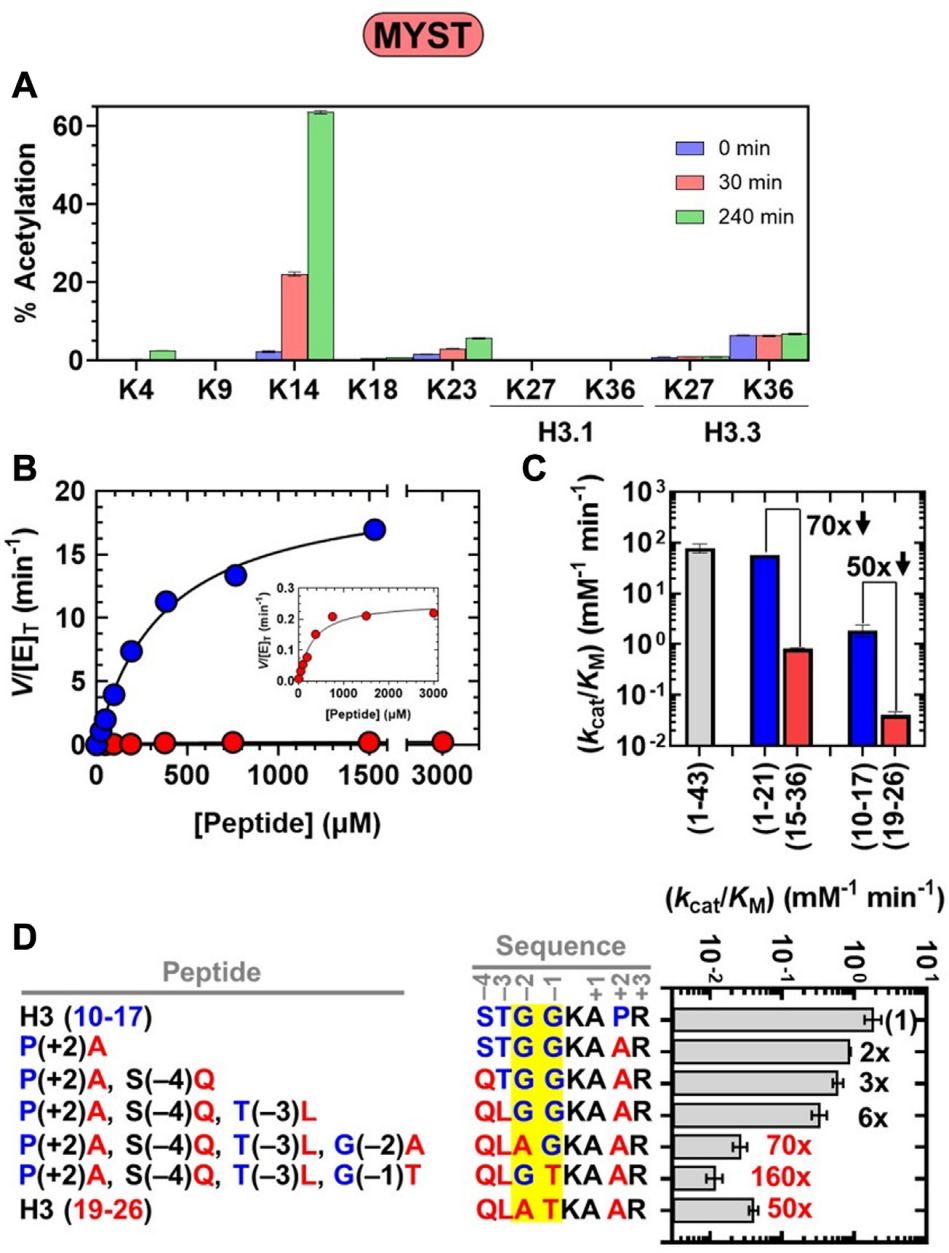
**Substrate preference of the KAT6A MYST domain**. *A*, histone proteomics study monitoring percent acetylation of lysine residues within the histone H3 tail following incubation of HeLa oligonucleosomes with the MYST domain. Values correspond to the average and standard deviation of three technical replicates. *B*, steady-state enzyme assays monitoring the rate of CoA formation (*V*/[E]_T_) at various [peptide] for H3 (1-21) (), and H3.3 (15-36) () with the KAT6A MYST domain. The inset shows the same data with the y-axis adjusted to view H3.3 (15-36). The lines are fits of the data to Equation 2 in Experimental procedures. *C*, *k*_cat_/*K*_M_ for MYST-catalyzed acetylation of H3 peptides. Values are from the data reported in Table 2. Numbers in bold refer to the fold-change in *k*_cat_/*K*_M_ for H3.3 (15-36) relative to H3 (1-21) and H3 (19-26) relative to H3 (10-17). The downward *arrow* denotes a decrease in activity for peptides containing K23 compared to those containing K14. *D*, *k*_cat_/*K*_M_ for MYST-catalyzed acetylation of H3 (10-17), H3 (19-26), and chimeric peptides. Values are from the data reported in Table S4. Positions −4 to +3 are numbered with respect to the lysine substrate. Numbers in bold refer to the fold-decrease in *k*_cat_/*K*_M_ relative to H3 (10-17). Values in *red* are meant to highlight the sensitivity of acetyltransferase activity towards changes at the −1 and −2 positions. Amino acid residues at the −1 and −2 positions are shaded in *yellow*.

To interrogate the intrinsic substrate specificity of the KAT6A MYST domain (residues 501–784), we characterized the acetyltransferase activity of this catalytic module toward oligonucleosomes purified from HeLa cells. KAT6A MYST domain was purified to homogeneity (Fig. S1*A*) and subsequently incubated with HeLa nucleosomes in the presence of acetyl-CoA. Sites of lysine acetylation within the N-terminal tails of histone proteins were quantified by mass spectrometry (MS) at the Northwestern Proteomics Core Facility, using a bottom-up approach (37).^2^ Within histone H3, K14 was preferentially modified over all other interrogated lysine residues, exhibiting a 20% and 61% increase in acetylation after 30 and 240 min, respectively (Fig. 2*A*). In addition, we observed significant acetylation of lysine residues within histone H4 (K5, K8, K12, and K16) and H2A (K5 and K9) (Fig. S2*A*). In contrast, we observed only a 4% increase in acetylation of H3K23, a 2.5% increase in acetylation of H3K4, and negligible (<1%) acetylation of all other interrogated lysine residues within H3 following a 240-min incubation (Fig. 2*A*).

Given that H3K23 is a known substrate of the KAT6A/B 4-plex (6, 15, 27), we sought to investigate the determinants that confer greater MYST-catalyzed acetylation of H3K14 relative to H3K23. Since both lysine residues are within the histone H3 tail, we asked if the H3 tail sequence was sufficient to observe the predominant acetylation of H3K14. Using the synthetic peptide H3.3 (1-43)^,^^3^ (Table 1) as substrate, MS analysis (Fig. S3) showed a greater increase in acetylation of H3K14 (61%) than H3K23 (7%), with negligible (<1%) acetylation of all other interrogated lysine residues. Thus, H3K14 is preferentially modified by the MYST domain regardless of whether the substrate is a peptide or nucleosome.

**Table 1:** H3 Peptides used in this work

Peptide	Sequence^a^
H3.3 (1-43)	ART KQT ARK STG G**K**_**14**_A PRK QLA T**K**_**23**_A ARK SAP STG GVK KPH RYR P
H3 (1-21)	ART KQT ARK STG G**K**_**14**_A PRK QLA
H3 (10-17)	STG G**K**_**14**_A PR
H3.3 (15-36)	A PRK QLA T**K**_**23**_A ARK SAP STG GVK
H3 (19-26)	QLA T**K**_**23**_A AR

a Lysine residues in bold correspond to K14 and K23.

We next sought to quantify the greater enzymatic activity towards H3K14 compared to H3K23 through steady-state assays with the truncated H3 peptides, H3 (1-21) and H3.3 (15-36) (Table 1). Acetyltransferase assays with these shortened peptides allow us to track K14 and K23 acetylation in two separate peptides, facilitating the acquisition of steady-state parameters (*k*_cat_ and *k*_cat_/*K*_M_) without requiring direct methods to interrogate the site(s) of acetylation (38). In addition, since enzymatic acetylation of H3 lysine residues apart from H3K14 and H3K23 is minimal (Figs. 2*A* and S3), the measured acetyltransferase activity towards H3 (1-21) and H3.3 (15-36) can be used as a proxy to evaluate acetylation at K14 and K23, respectively.

Acetylation of the H3 peptides was monitored through quantitation of acetyl-CoA and CoA *via* MS. The initial velocities (*V*) were then divided by the active enzyme concentration (E_T_) of the MYST domain, as determined from tight-binding inhibition by PF-9363 (Fig. S4*A* and Table S3). The steady-state kinetic data for H3 (1-21) and H3.3 (15-36) are shown in Figure 2*B*; for comparison, we also measured the acetyltransferase activity towards H3.3 (1-43) (Fig. S5*A*). Steady-state parameters for the H3 peptides are summarized in Table 2 and values of *k*_cat_/*K*_M_ are presented in Figure 2*C*.

**Table 2:** Steady-state parameters for KAT6A MYST domain and KAT6A^FL^^a^^,^^b^

Peptide	KAT6A MYST domain			KAT6A^FL^		
	(*k*_cat_/*K*_M_) (mM^−1^ min^-1^)	*k*_cat_ (min^−1^)	*K*_M_ (μM)	(*k*_cat_/*K*_M_) (mM^−1^ min^−1^)	*k*_cat_ (min^−1^)	*K*_M_ (μM)
H3.3 (1-43)	79 ± 16	9.8 ± 2	130 ± 2	3370 ± 960	10 ± 2	3.2 ± 2
H3 (1-21)	57 ± 0.1 (1)	22 ± 0.7	380 ± 20	1460 ± 740 (1)	21 ± 3	16 ± 6
H3.3 (15-36)	0.81 ± 0.04 (70)	0.30 ± 0.05	360 ± 80	11.2 ± 1 (130)	0.34 ± 0.04	30 ± 0.1
H3 (10-17)^c^	1.9 ± 0.5 (1)	≥3.5	≥1000	0.59 ± 0.3 (1)	≥1.6	≥1000
H3 (19-26)^c^	0.041 ± 0.006 (50)	≥0.088	≥1000	0.016 ± 0.002 (40)	≥0.075	≥1000

a 50 mM HEPES (pH 7.5), 5 mM NaCl, 0.1 mM EDTA, 0.002% Tween-20, and 20 μM AcCoA at 25 °C. Each value represents the average and standard deviation of 2 measurements.

b Values in parentheses refer to the fold-change in (*k*_cat_/*K*_M_) for H3.3 (15-36) *versus* H3 (1-21) or H3 (19-26) *versus* H3 (10-17) for each form of KAT6A.

c Lower limits are provided for *k*_cat_ and *K*_M_ due to the exceedingly high [peptide] needed to saturate *V*/[E]_T_.

The KAT6A MYST domain catalyzes acetylation of the H3 (1-21) and H3.3 (1-43) peptides with *k*_cat_/K_M_ values of 57 and 79 mM^−1^ min^−1^, respectively (Fig. 2*C*). As H3K14 is the primary acetylation product within H3.3 (1-43) (Fig. S3), the observed activity with H3 (1-21) is consistent with a model in which H3K14 is acetylated with this peptide. In contrast, for the H3.3 (15-36) peptide, we observed 70-fold less acetylation compared to H3 (1-21), with *k*_cat_/K_M_ = 0.81 mM^−1^ min^−1^ (Fig. 2*C*). The lower specificity constant measured for H3.3 (15-36), along with the minor H3K23 acetylation product detected for H3.3 (1-43) (Fig. S3) is consistent with a model in which H3K23 is not the preferred substrate of this form of KAT6A. Taken together, the above data show that the MYST domain preferentially modifies H3K14 over all other lysine residues, including H3K23, within the histone H3 tail.

### Residues proximal to H3K14 guide the substrate preference of the KAT6A MYST domain

Previous studies with the catalytic domain of KAT5 and the NuA4 complex, which includes the yeast MYST-family acetyltransferase Esa1, provided evidence that lysine residues with small amino acids such as glycine or alanine at the −1 position are preferentially acetylated by this enzyme (39, 40). Indeed, H3K14, the preferred substrate of the KAT6A MYST domain, follows glycine (G13) (Table 1). Moreover, from acetyltransferase assays with HeLa nucleosomes (Fig. S2*A*), we observe that lysine residues that exhibit substantial acetylation follow either glycine (H4K5, H4K8, H4K12, H2AK5, and H2AK9) or alanine (H4K16) (41). In contrast, H3K23, for which we detected minor acetylation product, follows threonine (T22) while H3K9 and H3K27, which exhibit negligible (<1%) acetylation, follow arginine (Table 1). To evaluate this model and further clarify the sequence determinants that mediate predominant H3K14 acetylation, we asked whether this preference is retained when using H3 (10-17) and H3 (19-26) (Table 1). These peptides retain only one lysine residue (H3K14 or H3K23) and the short (8 amino acids) length allows us to generate a series of chimeric peptides of these substrates to investigate the sequence-determinants that promote acetylation.

Michaelis-Menten plots for H3 (10-17) and H3 (19-26) are shown in Fig. S6*A* and steady-state parameters are reported in Table 2. Values of *k*_cat_/K_M_ for H3 (10-17) and H3 (19-26) are 20- to 30-fold lower than H3 (1-21) and H3.3 (15-36), respectively (Fig. 2*C*). This reduction in activity indicates that residues beyond H3 (10-17) and H3 (19-26) facilitate acetylation of K14 and K23, respectively. Nevertheless, H3 (10-17) was preferentially modified over H3 (19-26) by 50-fold, which is similar to the 70-fold preference for H3 (1-21) over H3.3 (15-36) (Fig. 2*C*). Thus, the sequence of these short peptides is sufficient to favor the acetylation of H3K14 over H3K23.

We subsequently assayed the enzymatic activity of the MYST domain towards a series of chimeric peptides of H3 (10-17) and H3 (19-26) (Fig. S7*A* and Table S4). Figure 2*D* shows *k*_cat_/*K*_M_ for these peptides and compares the effect of these changes relative to H3 (10-17).

Altering the identity of residues at the +2, −4, and −3 positions (P(+2)A, S(–4)Q, and T(–3)L, respectively) results in a modest (2-6-fold) decrease in MYST-catalyzed acetylation. However, subsequent changes that remove the glycine pair at the −1 and −2 positions have the largest effect on activity, decreasing *k*_cat_/*K*_M_ by approximately an order of magnitude. Compared to H3 (10-17), chimeras containing the G(–2)A and G(–1)T substitutions are 70- and 160-fold less efficient substrates, respectively; these effects are similar to what we observe with H3 (19-26). Conversely, the acetyltransferase activity towards H3 (19-26) is increased by approximately 10-fold when A21 and T22 are replaced with glycine; this chimera is only 6-fold less active than H3 (10-17). These observations indicate that changes in the sequence identity of residues at −1 and −2 have the largest effect on MYST-catalyzed acetylation, with glycine at both positions facilitating acetylation of the adjacent lysine residue.

### Structure of the KAT6A MYST domain with a H3K14-CoA bisubstrate inhibitor

**Figure 3:**
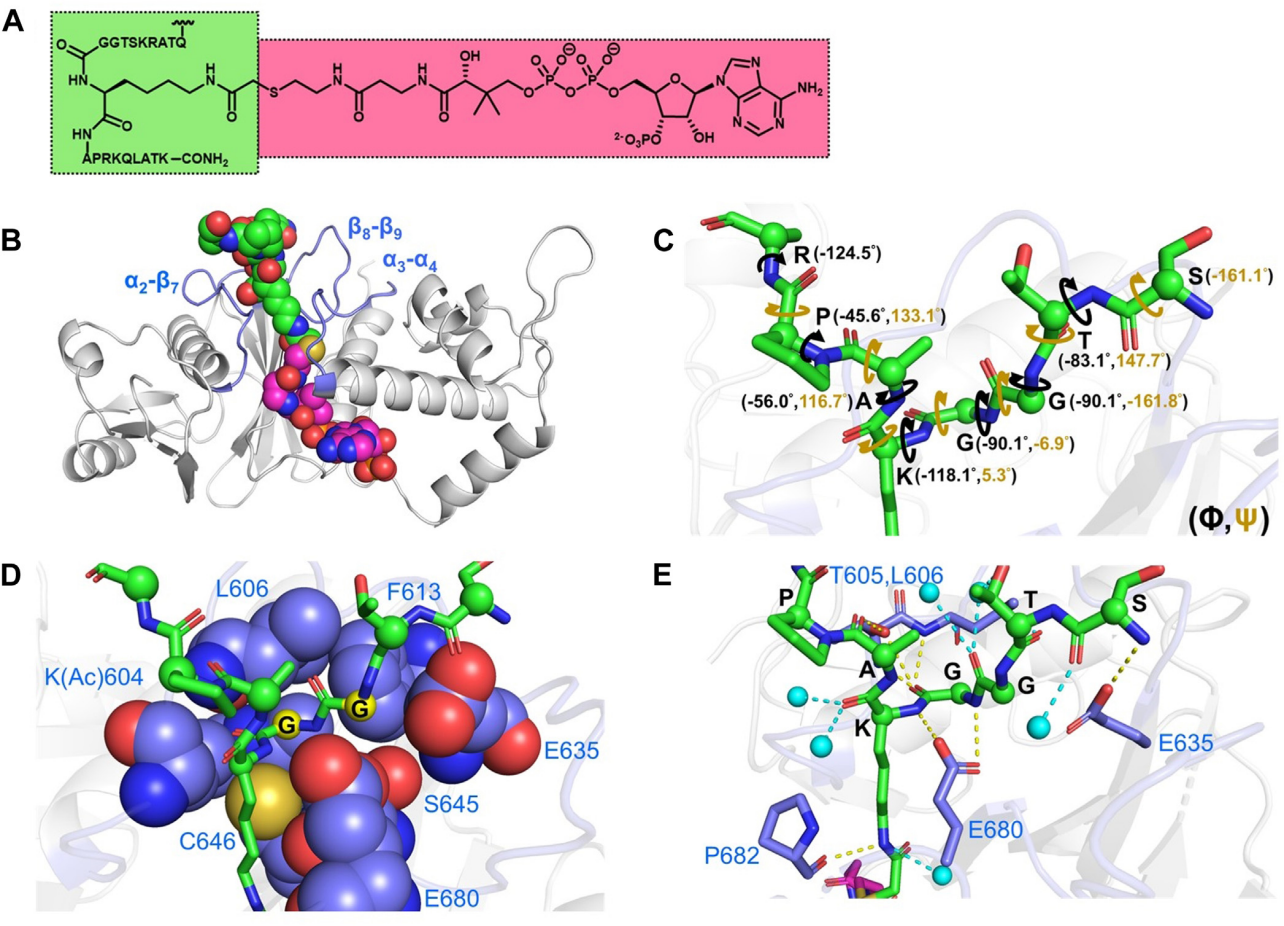
**Structure of the KAT6A MYST domain with the H3K14-CoA bisubstrate inhibitor**. *A*, structure of the H3K14-CoA bisubstrate inhibitor. The peptide and CoA portions of the inhibitor are shaded in *green* and *magenta*, respectively. The N-terminus of the bisubstrate inhibitor includes a 6-aminohexanoic acid linker that is neither shown nor resolved in the structure. *B*, view of the bisubstrate inhibitor (shown as spheres) bound to the KAT6A MYST domain. The peptide portion (H3 (10-17)) is colored *green* while the CoA portion is colored *magenta*. Loops flanking the peptide portion of the bisubstrate inhibitor (α_2_-β_7_, β_8_-β_9_, and α_3_-β_4_) are colored *blue*. *C*, dihedral angles for peptide backbone of bisubstrate inhibitor. Numbers in *black* and *brown* refer to ϕ and ψ angles, respectively. The α-carbon atom of each amino acid is represented as a sphere. *D*, residues proximal to G12 and G13 of the bisubstrate inhibitor. The α-carbon atoms of G12 and G13 are colored *yellow* and residues near this glycine pair are colored *blue* and shown as spheres. *E*, putative hydrogen bond interactions made with the peptide portion of the bisubstrate inhibitor. Hydrogen bond interactions are represented as *dashed lines*. Water molecules and water-mediated hydrogen bonds are colored in *cyan*. Hydrogen bonds between the enzyme and the peptide are colored in *yellow*.

Several crystal structures of the catalytic domain of MYST-family acetyltransferases have been reported (13, 40, 42, 43), but structures with bound histone substrates are limited (13, 40) and not available for KAT6A/B. To develop a physical model for molecular recognition of H3K14 by the MYST domain, we sought to obtain an x-ray structure of this module with bound H3 (1-21). While our efforts to obtain crystallographic data for this complex were unsuccessful, we were able to solve a crystal structure of the MYST domain bound to a H3K14-CoA bisubstrate inhibitor (Fig. 3*A*) (44). Such peptide-CoA conjugates have been utilized to interrogate the binding sites of acetyltransferases (45) so we evaluated how H3K14-CoA, which consists of H3 (5-23) with the ε-amino group of K14 covalently attached to CoA through a non-native acetyl bridge, is bound to the KAT6A MYST domain.

The structure of the KAT6A MYST domain with H3K14-CoA was solved at 1.72 Å resolution (Table S5), with interpretable electron density observed for residues 10 to 17 of the H3 peptide, the CoA moiety, and the acetyl bridge between the H3K14 ε-amino group and CoA (Fig. 3*B*). Comparing this structure with prior crystallographic models of the KAT6A MYST domain bound to acetyl-CoA and NuA4 complexed with a Htz1 peptide (Fig. S8) indicates that the CoA and peptide portions of the H3K14-CoA bisubstrate inhibitor largely overlap with acetyl-CoA and the Htz1 peptide, respectively. This observation implies that the bisubstrate inhibitor occupies both the acetyl-CoA and peptide binding sites of the KAT6A MYST domain, as expected.

The structure shows the peptide portion of the bisubstrate inhibitor surrounded by three loops within the MYST domain: α_2_-β_7_, β_8_-β_9_, and α_3_-α_4_ (Fig. 3*B*). Within this crevice, the bound peptide assumes a β-turn, resembling a type I conformation based on dihedral angles (ϕ, ψ) of G13 (−90.1°, −6.9°) and K14 (−118.1°, 5.3°) (Fig. 3*C*) (46, 47). In addition, we note that the α-carbon atoms G12 and G13 are in proximity (3.3–5.0 Å) to several residues within the α_2_-β_7_ (autoacetylated K604, L606, and F613), β_8_-β_9_ (E635, S645, and C646), and α_3_-α_4_ (E680) (Fig. 3*D*) loops. Steric restrictions within this environment provide a model for the preferred acetylation of lysine residues adjacent to a GG pair given the small size and conformational flexibility of glycine (48).

The peptide portion of the bisubstrate inhibitor is stabilized by several putative hydrogen bonds, including 8 water-mediated contacts and 8 direct contacts with the KAT6A MYST domain (Fig. 3*E* and Table S6). Notably, the vast majority (13/16) of these presumptive interactions are made with the peptide backbone; among the 8 direct hydrogen bonds within the MYST domain-peptide complex, 7 are made with the carbonyl and/or amide groups of S10, G13, K14, and A15 while the remaining hydrogen bond is made with the ε-amino group of K14 (Table S6). The lack of side-chain interactions is consistent with the above model that size and conformational flexibility, particularly at the −1 and −2 positions of the substrate, are critical determinants of molecular recognition by the MYST domain.

### KAT6A^FL^ retains specificity towards H3K14

Functional data indicate that the KAT6A MYST domain preferentially modifies H3K14 over H3K23 (Fig. 2). However, the activity and specificity of this domain could be influenced by histone recognition modules that are present outside of the MYST domain in KAT6A^FL^ (Fig. 1). For example, structural and biochemical data with the isolated double PHD finger domain of KAT6A^FL^ (*i.e.*, the DPF domain, Fig. 1) indicate that this reader domain binds to histone H3 tail peptides as well as analogs that contain acetyl and other acyl marks on H3K14 (27, 30, 49, 50, 51). These additional contacts may enhance H3 acetylation by KAT6A^FL^ compared to the MYST domain, as suggested from prior functional data (27, 30), and/or potentially alter the H3K14/H3K23 substrate preference of the MYST domain. To test this, we expressed and partially purified (Fig. S1*B*) KAT6A^FL^ and subsequently determined the substrate preference of this protein when presented with HeLa oligonucleosomes and histone H3 peptides.

**Figure 4:**
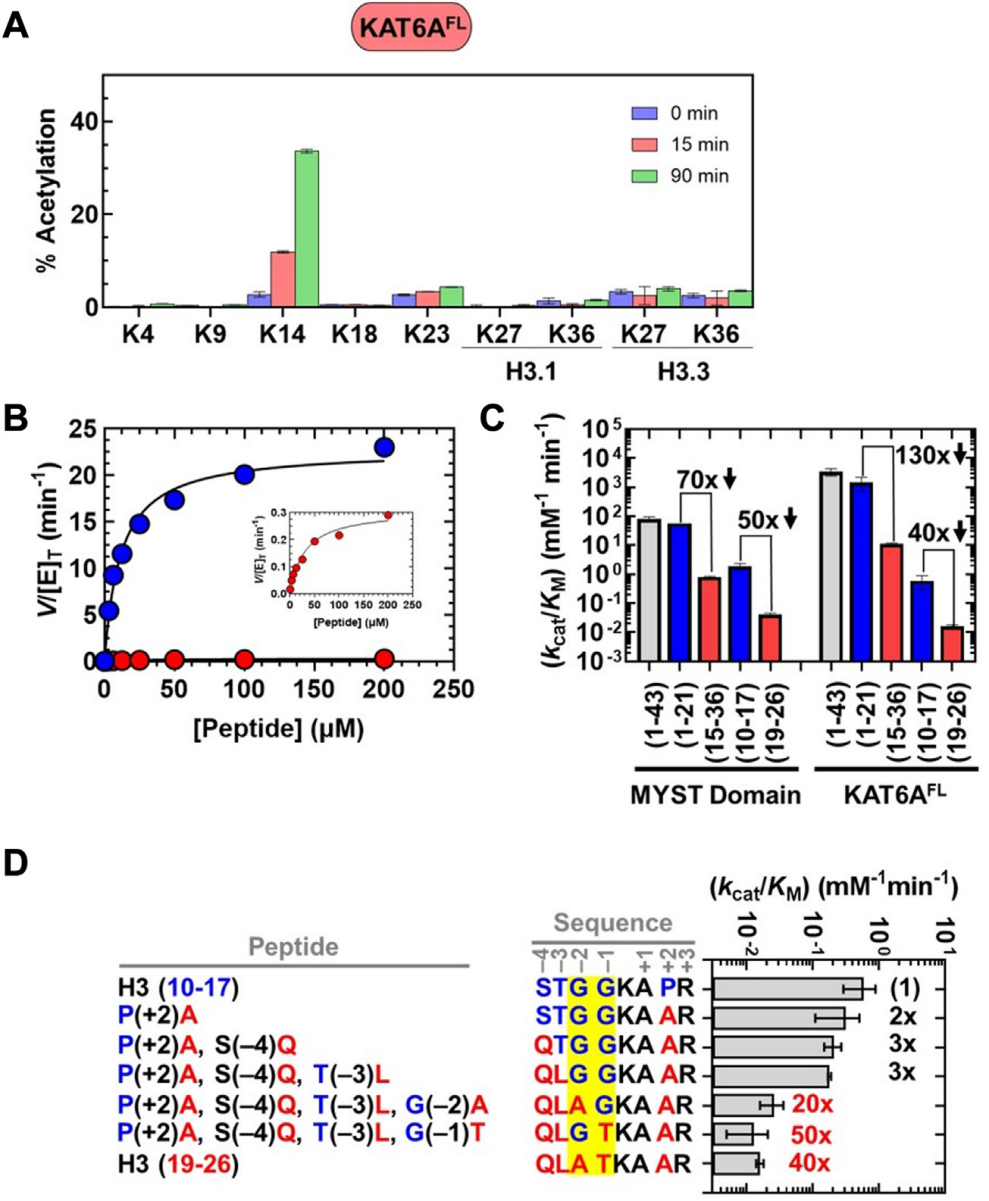
**Substrate preference of KAT6A**^**FL**^. *A*, histone proteomics study monitoring percent acetylation of lysine residues within the histone H3 tail following incubation of HeLa oligonucleosomes with KAT6A^FL^. Values correspond to the average and standard deviation of three technical replicates. *B*, steady-state enzyme assays monitoring the rate of CoA formation (*V*/[E]_T_) at various [peptide] for H3 (1-21) () and H3.3 (15-36) () with KAT6A^FL^. The inset shows the same data with the y-axis adjusted to view H3.3 (15-36). The lines are fits of the data to Equation 2 in Materials and Methods. *C*, *k*_cat_/*K*_M_ for acetylation of H3 peptides by KAT6A^FL^ and, for comparison, the MYST domain. Values are from the data reported in Table 2. Numbers in bold refer to the fold-change in *k*_cat_/*K*_M_ for H3.3 (15-36) relative to H3 (1-21) and H3 (19-26) relative to H3 (10-17) for each form of KAT6A. The downward arrow denotes a decrease in activity for peptides containing K23 compared to those containing K14. *D*, *k*_cat_/*K*_M_ for KAT6A^FL^-catalyzed acetylation of H3 (10-17), H3 (19-26), and chimeric peptides. Values are from the data reported in Table S4. Positions −4 to +3 are numbered with respect to the lysine substrate. Numbers in bold refer to the fold-decrease in *k*_cat_/*K*_M_ relative to H3 (10-17). Values in *red* are meant to highlight the sensitivity of acetyltransferase activity towards changes at the −1 and −2 positions. Amino acid residues at the −1 and −2 positions are shaded in *yellow*.

H3K14 was preferentially acetylated by KAT6A^FL^ over all interrogated histone lysine residues within the histone H3 tail, as evidenced by a 31% increase in acetylation of this lysine after a 90-min incubation (Fig. 4*A*). In contrast, we observed a minor increase in acetylation of H3K23 (1.7%) and negligible (<1%) acetylation for all other lysine residues (Fig. 4*A*). Besides the H3 tail, KAT6A^FL^ acetylated lysine residues within H4 (K5, K8, K12, and K16) and H2A (K5 and K9) (Fig. S2*B*). These results mirror our observations with the KAT6A MYST domain (Figs. 2*A* and S2*A*).

We subsequently determined the acetyltransferase activity of KAT6A^FL^ through steady-state assays with H3 (1-21) and H3.3 (15-36) (Fig. 4*B*) and, for comparison, H3.3 (1-43) (Fig. S5*B*). The active enzyme concentration for KAT6A^FL^ was determined through tight-binding inhibition by PF-9363 (Fig. S4*B* and Table S3), allowing us to directly compare the enzymatic activities of KAT6A^FL^ and the KAT6A MYST domain. Steady-state parameters are summarized in Table 2 and values of *k*_cat_/*K*_M_ for KAT6A^FL^ and the MYST domain (reproduced from Fig. 2*C* to facilitate comparison) are shown in Figure 4*C*.

KAT6A^FL^ acetylates H3 (1-43), H3 (1-21), and H3.3 (15-36) with *k*_cat_/K_M_ values of 3,370, 1,460, and 11.2 mM^−1^ min^−1^, respectively (Fig. 4*C*). These values are 10- to 40-fold greater than corresponding measurements made with the MYST domain (Fig. 4*C*), indicating that the additional interactions made by KAT6A^FL^, such as those involving the DPF domain (27, 30), enhance acetylation of the H3 peptides. Despite this difference, we observe greater KAT6A^FL^ acetyltransferase activity (130-fold) towards H3 (1-21) compared to H3.3 (15-36). This observation is consistent with a model that KAT6A^FL^, like the MYST domain, preferentially acetylates H3K14 over H3K23.

We subsequently probed the local sequence-determinants of H3K14 acetylation by KAT6A^FL^ by measuring steady-state parameters for H3 (10-17) and H3 (19-26) (Fig. S6*B*) as well as the panel of peptide chimeras tested with the KAT6A MYST domain (Fig. S7*B*). As summarized in Table 2 and Figure 4*C*, truncation of H3 (1-21) to H3 (10-17) and H3.3 (15-36) to H3 (19-26) lowers *k*_cat_/K_M_ by 2470- and 700-fold, respectively. This large decrease in activity presumably arises from the ablation of contacts made between KAT6A^FL^ (*e.g.* the DPF domain) and residues distal to H3 (10-17) and H3 (19-26). Nevertheless, *k*_cat_/K_M_ for H3 (10-17) is approximately 40-fold greater than what we observe with H3 (19-26), similar to our observations with the MYST domain (Fig. 4*C*). In addition, KAT6A^FL^ shows a substantial preference for peptides with a glycine pair adjacent to lysine (Fig. 4*D*; and see Fig. S7*B* and Table S4). Chimeras that do not retain a glycine pair are the least reactive substrates for KAT6A^FL^, in agreement with our findings with the MYST domain (Fig. 2*D*). Taken together, these results indicate that while KAT6A^FL^ enhances acetylation of longer H3 peptides compared to the MYST domain, both enzymes preferentially modify H3K14 over H3K23.

### The KAT6A^FL^ 4-plex alters the substrate preference of KAT6A^FL^ from H3K14 to H3K23

KAT6A^FL^ forms a tetrameric complex *in vivo* with the scaffold protein BRPF1 and two accessory proteins, ING4/5 and MEAF6 (Fig. 1). With HeLa nucleosomes or H3 peptides as substrate, prior results show that the reassembled KAT6A/B 4-plex modifies H3K23 *in vitro* (6, 15, 27). Given that we observe predominant H3K14 acetylation by the MYST domain and KAT6A^FL^, we sought to evaluate and quantitate the K14/K23 substrate preference with the KAT6A^FL^ 4-plex.

**Figure 5:**
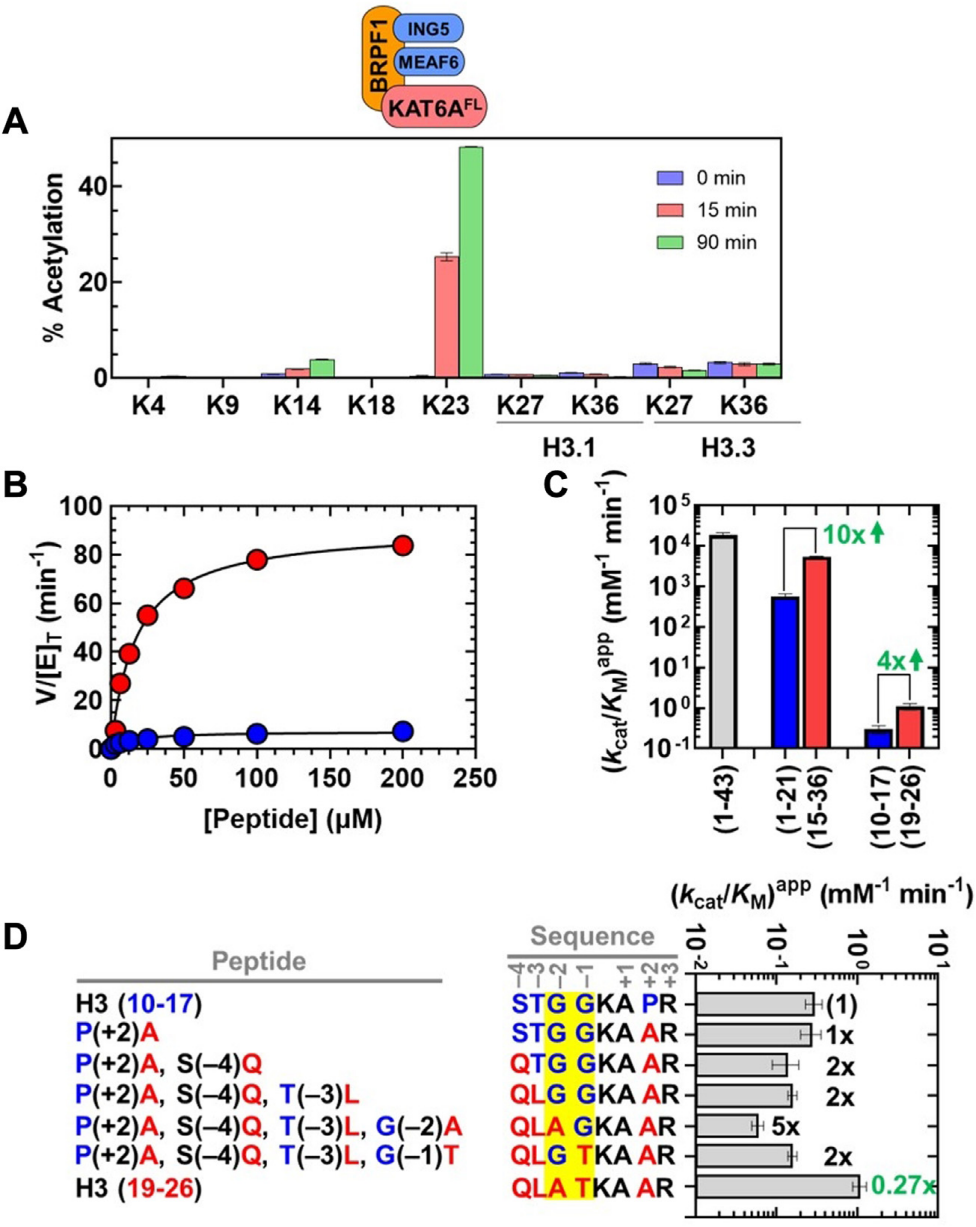
**Substrate preference of the KAT6A**^**FL**^**4-plex.***A*, histone proteomics study monitoring percent acetylation of lysine residues within the histone H3 tail following incubation of HeLa oligonucleosomes with the KAT6A^FL^ 4-plex (data reproduced from (6)). Values correspond to the average and standard deviation of three technical replicates. *B*, steady-state enzyme assays monitoring the rate of CoA formation (*V*/[E]_T_) at various [peptide] for H3 (1-21) (), and H3.3 (15-36) () with the KAT6A^FL^ 4-plex. The lines are fits of the data to Equation 2 in Materials and Methods. *C*, (*k*_cat_/*K*_M_)^app^ for acetylation of H3 peptides by the KAT6A^FL^ 4-plex. Values are from the data reported in Table 3. Numbers in *green* and bold refer to the fold-change in *k*_cat_/*K*_M_ for H3.3 (15-36) relative to H3 (1-21) and H3 (19-26) relative to H3 (10-17). The *green* upward arrow denotes an increase in activity for peptides containing K23 compared to those containing K14. *D*, *k*_cat_/*K*_M_ for acetylation of H3 (10-17), H3 (19-26), and chimeric peptides by the KAT6A^FL^ 4-plex. Values are from the data reported in Table S4. Positions −4 to +3 are numbered with respect to the lysine substrate. Numbers in bold refer to the fold-decrease in *k*_cat_/*K*_M_ relative to H3 (10-17). The value in *green* denotes the 4-fold (1/0.27) greater acetyltransferase activity towards H3 (19-26) compared to H3 (10-17). Amino acid residues at the −1 and −2 positions are shaded in *yellow*.

We first reproduce data from our prior investigation (6) showing the extent of lysine acetylation on histone H3 following incubation of HeLa nucleosomes with the KAT6A^FL^ 4-plex (Fig. 5*A*). H3K23 was the preferred substrate of the KAT6A^FL^ 4-plex, with a 48% increase in acetylation of this residue following a 90-min incubation. In contrast, we only observed a 4% increase in acetylation of H3K14 and a <1% change in acetylation for all other H3 lysine residues after 90 min. These results noticeably differ from our findings with the MYST domain (Fig. 2*A*) and KAT6A^FL^ (Fig. 4*A*). In addition, we detected little acetyltransferase activity (≤4% increase over 90 min) towards interrogated lysine substrates within the H4 and H2A tails (Fig. S2*C*), in contrast to the substantial modification we observed for these residues with the MYST domain and KAT6A^FL^ (Fig. S2, *A* and *B*, respectively). The absence of significant acetylation at H4 and H2A is consistent with prior observations that the KAT6A^FL^ 4-plex stimulates acetylation of histone H3 over H4 and H2A (29, 52).

To quantify the preferred acetylation of H3K23 over H3K14 by the KAT6A^FL^ 4-plex, we expressed and partially purified (Fig. S1*C*) the KAT6A^FL^ 4-plex and performed steady-state assays with this form of KAT6A with H3 (1-21) and H3.3 (15-36) (Fig. 5*B*) as well as H3.3 (1-43) (Fig. S5*C*). Initial rates were normalized with respect to the active enzyme concentration (Fig. S4*C* and Table S3) for the KAT6A^FL^ 4-plex and values of ${\left(k_{\text{cat}}/K_{M}\right)}^{\text{app}}$ and $k_{\text{cat}}^{\text{app}}$ are reported in Table 3. The superscript “app” included in these terms is meant to denote that they are apparent values as reactions with the KAT6A^FL^ 4-protein complex may include the uncomplexed form of KAT6A^FL^.

**Table 3:** Steady-state parameters for KAT6A^FL^ and the MYST domain in the presence of BRPF1^a^^,^^b^^,^^c^

	KAT6A^FL^ 4-plex			KAT6A^FL^ 2-plex			MYST 2-plex		
Peptide	(*k*_cat_/*K*_M_)^app^ (mM^−1^ min^−1^)	$k_{\mathrm{cat}}^{\mathrm{app}}$ (min^−1^)	*K*_M_ (μM)	(*k*_cat_/*K*_M_)^app^ (mM^−1^ min^−1^)	$k_{\mathrm{cat}}^{\mathrm{app}}$ (min^−1^)	*K*_M_ (μM)	(*k*_cat_/*K*_M_)^app^ (mM^−1^ min^−1^)	$k_{\mathrm{cat}}^{\mathrm{app}}$ (min^−1^)	*K*_M_ (μM)
H3.3 (1-43)	18,200 ± 2800	90 ± 4	5.0 ± 1	21,800 ± 1800	130 ± 10	5.9 ± 1	30,600 ± 2600	47 ± 0.01	1.5 ± 0.1
H3 (1-21)	570 ± 90 (1)	7.6 ± 0.6	13 ± 0.9	620 ± 30 (1)	7.0 ± 0.6	11 ± 1	900 ± 200 (1)	12 ± 0.5	14 ± 3
H3.3 (15-36)	5420 ± 200 (0.1)	89 ± 4	16 ± 1	6190 ± 700 (0.1)	84 ± 10	13 ± 0.2	820 ± 300 (1)	28 ± 2	36 ± 9
H3 (10-17)^d^	0.30 ± 0.07 (1)	≥0.40	≥1000	0.39 ± 0.06 (1)	≥0.72	≥1000	0.67 ± 0.06 (1)	≥1.4	≥1000
H3 (19-26)^d^	1.1 ± 0.2 (0.27)	≥2.0	≥1000	1.4 ± 0.2 (0.28)	≥2.2	≥1000	0.18 ± 0.07 (4)	≥0.46	≥1000

a 50 mM HEPES (pH 7.5), 5 mM NaCl, 0.1 mM EDTA, 0.002% Tween-20, and 20 μM AcCoA at 25 °C. Each value represents the average and standard deviation of 2 measurements.

b Values in parentheses refer to the fold-change in (*k*_cat_/*K*_M_)_^app^_ for H3.3 (15-36) *versus* H3 (1-21) or H3 (19-26) *versus* H3 (10-17) for each form of KAT6A.

c $k_{\text{cat}}^{\text{app}}$ and (*k*_cat_/*K*_M_)^app^ include the superscript *“*app” to denote that these values are apparent because the uncomplexed form of KAT6A^FL^ (or the MYST domain) may be present in reactions.

d Lower limits are provided for $k_{\text{cat}}^{\text{app}}$ and *K*_M_ due to the exceedingly high [peptide] needed to saturate *V*/[E]_T_.

The KAT6A^FL^ 4-plex catalyzes acetylation of H3.3 (1-43) and H3.3 (15-36) with ${\left(k_{\text{cat}}/K_{M}\right)}^{\text{app}}$ values of 18,200 and 5420 mM^−1^ min^−1^, respectively (Fig. 5*C*). These values are 32- and 10-fold greater than what we measured for H3 (1-21) (570 mM^−1^ min^−1^), respectively (Fig. 5*C*). The increased activity measured for H3.3 (15-36) is consistent with a model in which H3K23 is preferred over H3K14 by the KAT6A^FL^ 4-plex and supports the use of shorter H3 peptides to investigate H3K23 acetylation by this form of KAT6A.

Given the observed sensitivity of MYST- and KAT6A^FL^-catalyzed acetylation of H3K14 to changes at the −1 and −2 positions (Figs. 2*D* and 4*D*, respectively), we wanted to know whether the observed stimulation of H3K23 acetylation by the KAT6A^FL^ 4-plex is guided by sequence-specific recognition of residues adjacent to H3K23. Alternatively, interactions made between the 4-plex and more distal residues within the H3 tail may facilitate the acetylation of K23. These models are not mutually exclusive, and both may contribute to the activity and substrate specificity of the KAT6A^FL^ 4-plex.

To evaluate the above models, we compared the acetyltransferase activities of the truncated H3 peptides, H3 (10-17) and H3 (19-26) (see Fig. S6*C* and Table 3). As summarized in Figure 5*C*, truncation of H3 (1-21) to H3 (10-17) and H3.3 (15-36) to H3 (19-26) lowers ${\left(k_{\text{cat}}/K_{M}\right)}^{\text{app}}$ by 1900- and 4930-fold, respectively. The reduced acetyltransferase activity towards these peptides highlights the importance of interactions made between the KAT6A^FL^ 4-plex and H3 residues distal to H3 (10-17) and H3 (19-26). Despite this reduced activity, we observe a 4-fold preference for acetylation of H3 (19-26) over H3 (10-17). This preference is in stark contrast to our observations with KAT6A^FL^ and the MYST domain, where acetylation of H3 (10-17) is strongly preferred (Fig. 4*C*).

To determine the local sequence-dependence of this preference, we monitored the acetyltransferase activities of the KAT6A^FL^ 4-plex with chimeras of H3 (10-17) and H3 (19-26). As summarized in Figure 5*D* (and see Fig. S7*C* and Table S4), the preferred acetylation of H3 (19-26) over H3 (10-17) was lost when A21 and/or T22 were replaced with glycine (Fig. 5*D*); chimeras containing these substitutions lowered (*k*_cat_/K_M_) by approximately 10- to 20-fold. These observations contrast with what we observe with the KAT6A MYST domain (Fig. 2*D*) and KAT6A^FL^ (Fig. 4*D*), where lysine acetylation is facilitated by an adjacent glycine pair. Thus, the KAT6A^FL^ 4-plex shifts the substrate preference of KAT6A^FL^ from H3K14 to H3K23 through changes in molecular recognition of the −1 and −2 residues.

### BRPF1 modulates the substrate preference of KAT6A^FL^ and the MYST domain

Acetyltransferase assays with HeLa nucleosomes and histone peptides indicate that the KAT6A^FL^ 4-plex shifts the substrate preference of KAT6A^FL^ from H3K14 to H3K23 (Fig. 5). Prior cellular and *in vitro* data (6, 15, 52) strongly suggest that the scaffold protein, BRPF1, promotes acetylation of this residue by KAT6A^FL^. To directly demonstrate this, we expressed and partially purified (Fig. S1*D*) the recombinant KAT6A^FL^-BRPF1 2-protein complex (KAT6A^FL^ 2-plex) to test whether BRPF1 could shift the substrate preference of KAT6A^FL^ from H3K14 to H3K23 using HeLa nucleosomes and histone peptides as substrates.

**Figure 6:**
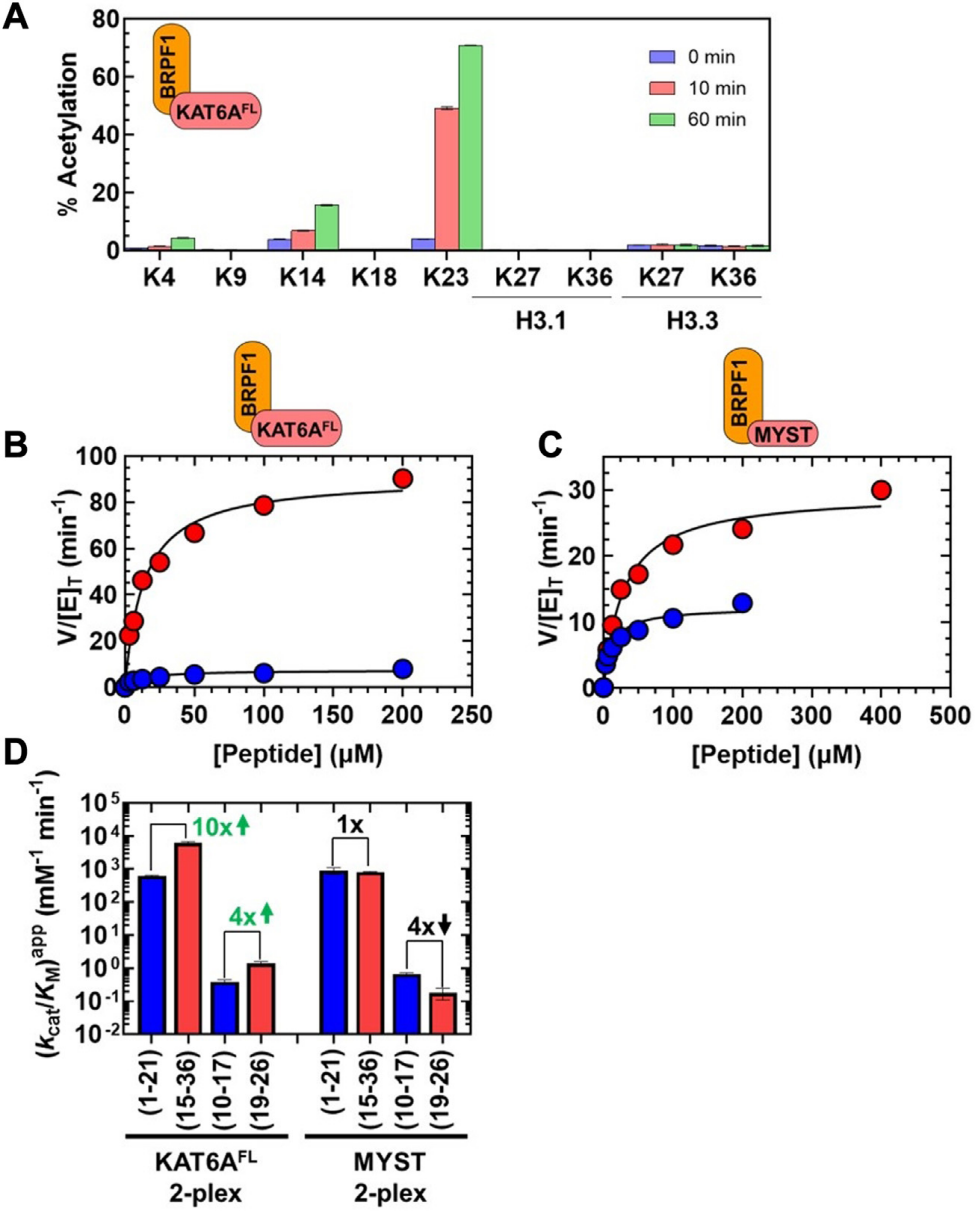
**BRPF1 Promotes H3K23 Acetylation by KAT6A**. *A*, Histone proteomics study monitoring percent acetylation of lysine residues within the histone H3 tail following incubation of HeLa oligonucleosomes with the KAT6A^FL^ 2-plex. Values correspond to the average and standard deviation of three technical replicates. *B* and *C*, steady-state enzyme assays monitoring the rate of CoA formation (*V*/[E]_T_) at various [peptide] for H3 (1-21) (), and H3.3 (15-36) () by the KAT6A^FL^ 2-plex (*B*) and MYST 2-plex (*C*). The lines fit the data to Equation 2 in Experimental procedures. *D*, (*k*_cat_/*K*_M_)^app^ for acetylation of H3 peptides by the KAT6A^FL^ and MYST 2-plexes. Values are from the data reported in Table 3. Numbers in bold refer to the fold-change in *k*_cat_/*K*_M_ for H3.3 (15-36) relative to H3 (1-21) and H3 (19-26) relative to H3 (10-17) for each form of KAT6A. The *green* upward arrow denotes an increase in activity for K23 peptides compared to K14 peptides, while the *black* downward arrow indicates a decrease in activity for the K23 peptide compared to the K14 peptide.

Figure 6*A* shows the extent of H3 lysine acetylation following incubation of HeLa oligonucleosomes with the KAT6A^FL^ 2-plex. Like the KAT6A^FL^ 4-plex, we observe preferential acetylation of H3K23, with a 67% increase in acetylation of this residue following a 60-min incubation. In comparison, we only observe a 12% increase in acetylation of H3K14, a 4% increase in acetylation for H3K4, and negligible (<1%) acetylation for all other H3 lysine residues at 60 min. In addition, acetylation of H4 and H2A lysine residues (Fig. S2*D*) by the KAT6A^FL^ 2-plex was lower compared to KAT6A^FL^ (Fig. S2*B*) and the MYST domain (Fig. S2*A*). This attenuation is similar to what we observed with the KAT6A^FL^ 4-plex (Fig. S2*C*).

We quantified the substrate preference of the KAT6A^FL^ 2-plex towards H3K14 and H3K23, through steady-state assays with H3 (1-21) and H3.3 (15-36), respectively (Fig. 6*B*). Steady state data were normalized with respect to the active enzyme concentration (Fig. S4*D* and Table S3), and values of ${\left(k_{\text{cat}}/K_{M}\right)}^{\text{app}}$ and $k_{\text{cat}}^{\text{app}}$ are reported in Table 3. Like the KAT6A^FL^ 4-plex (Fig. 5*B*), the KAT6A^FL^ 2-plex exhibits approximately 10-fold greater acetyltransferase activity towards H3.3 (15-36) than H3 (1-21) (Fig. 6*B*), with ${\left(k_{\text{cat}}/K_{M}\right)}^{\text{app}}$ = 6190 and 620 mM^−1^ min^−1^, respectively (Fig. 6*D*).

To further evaluate the properties of the KAT6A^FL^ 2-plex, we monitored the acetylation of the H3 (10-17) and H3 (19-26) peptides (Fig. S6*D*). While acetylation of these peptides is approximately 10^3^-fold less efficient compared to H3 (1-21) and H3.3 (15-36), we observe that the KAT6A^FL^ 2-plex prefers H3 (19-26) over H3 (10-17) by approximately 4-fold (${\left(k_{\text{cat}}/K_{M}\right)}^{\text{app}}$ = 1.4 and 0.39 mM^−1^ min^−1^) (Fig. 6*D*). These effects are nearly identical to what we observed with the KAT6A^FL^ 4-plex (Fig. 5*C*) and thus indicate that BRPF1 alone is sufficient to change the substrate preference of KAT6A^FL^ from H3K14 to H3K23.

Finally, we examined the substrate preference of the KAT6A MYST domain when bound to BRPF1. Previous studies have demonstrated that the MYST domain interacts with BRPF1 (29) and that this complex is capable of acetylating H3K23 (15). To quantitate the acetyltransferase activity of this form of KAT6A towards H3K14 and H3K23, we partially purified the MYST-BRPF1 2-protein complex (MYST 2-plex, Fig. S1*E*) and assessed its activity using H3 peptides as substrates (Figs. 6*C* and S6*E*). Initial velocities for the MYST 2-plex, normalized with respect to the active enzyme concentration (Fig. S4*E* and Table S3), are summarized in Table 3.

For acetylation of H3 (1-21) and H3.3 (15-36) by the MYST 2-plex (Fig. 6*C*), we measure ${\left(k_{\text{cat}}/K_{M}\right)}^{\text{app}}$ values of 900 and 820 mM^−1^ min^−1^, respectively (Fig. 6*D*). These values are nearly identical and thus indicate that the MYST 2-plex modifies these peptides with little discrimination (Fig. 6*D*, 1×). Moreover, this selectivity is intermediate between KAT6A^FL^ 2-plex, which prefers H3.3 (15-36) over H3 (1-21) (Fig. 6*D*, 10×), and the KAT6A MYST domain, which prefers H3 (1-21) over H3.3 (15-36) (Fig. 2*C*, 70×). A similar trend is observed for acetylation of H3 (10-17) and H3 (19-26) (Fig. S6*E*): the MYST 2-plex prefers H3 (10-17) over H3 (19-26) (Fig. 6*D*, 4×) but to a lesser extent compared to the MYST domain (Fig. 2*C*, 50×). In contrast, the KAT6A^FL^ 2-plex prefers H3 (19-26) over H3 (10-17) by 4-fold (Fig. 6*D*). These results indicate that BRPF1 promotes H3K23 acetylation by the MYST domain but to a lesser extent compared to KAT6A^FL^ when complexed with BRPF1.^4^

## Discussion

Therapeutic targeting of the MYST family of acetyltransferases is an emerging strategy in the treatment of cancer (4, 5). However, the development of small-molecule inhibitors of these enzymes presents three unique challenges. First, MYST family members share a highly conserved acetyltransferase domain (12, 13) and, therefore, drug candidates that target this module of a given MYST member must do so with selectivity to avoid off-target effects. Second, MYST members form distinct multiprotein complexes in cells (Table S1) and effective inhibitors must stably bind to these enzymes in their complexed forms. Finally, the multiprotein assemblies formed by MYST members guide their substrate specificities in cells (Table S1). For example, KAT7-BRPF(2/3) complexes modify H3K14 while KAT7-JADE(1/2/3) complexes modify H4K5, H4K8, and H4K12 (16, 17, 18, 19, 20, 52, 53). In addition, the KAT8 MSL complex modifies H4K16 whereas the KAT8 NSL complex modifies H4K5 and H4K8 (21, 22, 23, 24, 25). Therefore, understanding the substrate preference of MYST enzymes and their modulation by associated proteins is a critical step towards delineating the pharmacodynamic biomarkers of drug candidates.

The data presented here clarify the substrate preference of the uncomplexed and complexed forms of KAT6A. Within histone H3, our results provide strong evidence that H3K14 is the preferred substrate of KAT6A^FL^ and the KAT6A MYST domain. However, the formation of the KAT6A^FL^ 4-plex shifts this preference from H3K14 to H3K23 and we show that the scaffold protein BRPF1 mediates this change in substrate specificity. In addition, H3K9 and H3K27, which have been implicated as substrates for KAT6A (31, 32, 33, 34, 35, 36), do not undergo detectable (<1%) acetylation in assays with all tested forms of KAT6A. These findings are in agreement with recent cellular data which do not show significant global changes in H3K9 nor H3K27 following knockout of *KAT6A/B* or *BRPF1* (6, 15) and, taken together imply that these lysine residues are neither substrates for KAT6A/B alone nor for the KAT6A/B 4-plex. Modest changes in the cellular levels of acetylated H3K9Ac (31, 32, 33, 34, 35, 36) and H3K27Ac (33) have been observed in other studies and we propose these effects occur through indirect mechanisms following disruption of *KAT6A* and/or *KAT6B* expression. Alternatively, cross-reactivity of the antibodies used to detect acetylated H3K9 and H3K27 *via* immunoblotting (54) may obscure interrogation of these lysine residues.

While KAT6A^FL^ and the KAT6A^FL^ 4-plex acetylate H3K14 and H3K23, respectively, double knockout of *KAT6A* and *KAT6B* in cells results in a large decrease in acetylated H3K23 (6). In contrast, modest changes in acetylated H3K14 are observed (6), and remaining levels of this mark are likely mediated by KAT7-BRPF(2/3) complexes (6, 16, 17, 18, 55). The substantial reduction in H3K23 acetylation following loss of *KAT6A/B* is consistent with our finding that KAT6A^FL^, when complexed with BRPF1, exhibits greater acetyltransferase activity towards H3K23 than H3K14. A preference towards H3K23 over H3K14 was also inferred from *in vitro* studies with the KAT6B 4-plex (27). Since BRPF1 is essential for acetylation of H3K23 (6, 15, 56, 57), these results collectively provide strong evidence that H3K23 is the preferred substrate of KAT6A/B-BRPF1 complexes in cells. Moreover, they support the use of acetylated H3K23 as a cellular biomarker to evaluate the pharmacology of KAT6A/B inhibitors (6).

### Using histone H3 peptides to quantitate changes in the substrate preference of KAT6A

As the KAT6A^FL^ 4-plex alters the substrate preference of KAT6A^FL^ from H3K14 to H3K23, we sought to quantify this change in specificity. Our studies demonstrate that the preference of KAT6A^FL^ and the KAT6A^FL^ 4-plex towards H3K14 and H3K23, respectively, is retained in acetyltransferase assays with peptides that resemble the histone H3 tail. This observation facilitates the use of steady-state kinetic measurements with the H3 (1-21) and H3.3 (15-36) peptides, which track K14 and K23 acetylation, respectively, in two separate peptides. Steady-state experiments afford determination of *k*_cat_, the turnover number which sets a lower limit for any first-order rate constant after substrate binding (*e.g.* the chemical step, a conformational change before or after chemistry, and/or product release), and *k*_cat_/*K*_M_, the specificity constant which corresponds to the apparent-second order rate constant for substrate association followed by conversion to product (58). While the intrinsic rate constants embedded within these steady-state parameters cannot be extracted without additional information, the ratio of *k*_cat_/*K*_M_ for two competing substrates can be used to measure the substrate specificity of KAT6A^FL^ towards peptides that contain K14 or K23 (59).

**Figure 7:**
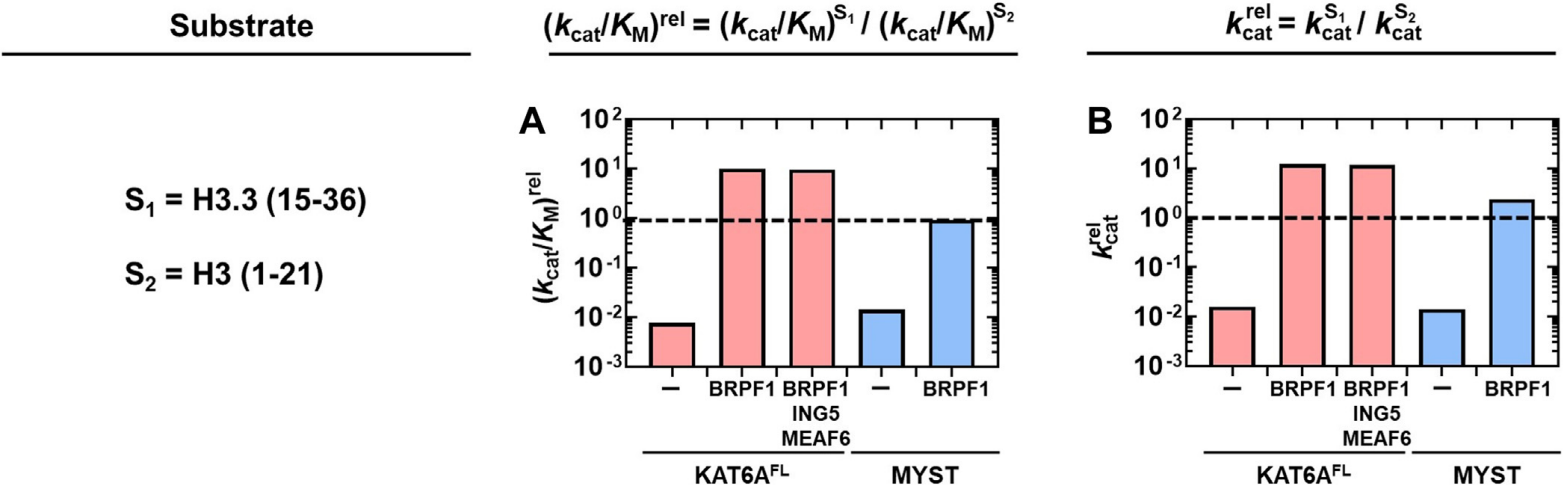
**Changes in steady-state parameters for H3.3 (15-36) relative to that for H3 (1-21) for different forms of KAT6A.***A*, (*k*_cat_/*K*_M_)^rel^ refers to *k*_cat_/*K*_M_ (or (*k*_cat_/*K*_M_)^app^) for H3.3 (15-36) relative to that for H3 (1-21) for each form of KAT6A. *B*, $k_{\text{cat}}^{\text{rel}}$ refers to *k*_cat_ (or $k_{\text{cat}}^{\text{app}}$) for H3.3 (15-36) relative to that for H3 (1-21) for each form of KAT6A. The dashed line corresponds to (*k*_cat_/*K*_M_)^rel^ or $k_{\text{cat}}^{\text{rel}}$ = 1.

Figure 7 summarizes (*k*_cat_/*K*_M_)^rel^, which corresponds to *k*_cat_/*K*_M_ for H3.3 (15-36) relative to H3 (1-21), for the uncomplexed and complexed forms of KAT6A^FL^. With KAT6A^FL^, *k*_cat_/K_M_ for H3.3 (15-36) is 130-fold lower than that for H3 (1-21) (*i.e.*. (*k*_cat_/*K*_M_)^rel^ = 7.7 × 10^-3^). However, formation of the KAT6A^FL^ 4-plex changes this preference so that (*k*_cat_/*K*_M_)^app^ for the H3.3 (15-36) peptide is 10-fold greater than that for H3 (1-21) (*i.e.*. (*k*_cat_/*K*_M_)^rel^ = 10). This approximately 10^3^-fold change in (*k*_cat_/*K*_M_)^rel^ is also observed with the KAT6A^FL^ 2-plex, which indicates that the BRPF1 scaffold protein alters the substrate preference of KAT6A^FL^ towards these peptides by three orders of magnitude.^5^

We also investigated $k_{\text{cat}}^{\text{rel}}$ for each tested form of KAT6A^FL^ (Fig. 7). This comparison indicates that changes in (*k*_cat_/*K*_M_)^rel^ correlate with changes in $k_{\text{cat}}^{\text{rel}}$. For example, KAT6A^FL^ catalyzes acetylation of the H3.3 (15-36) peptide with a *k*_cat_ value that is approximately 60-fold lower than that for H3 (1-21) ($k_{\text{cat}}^{\text{rel}}$ = 0.016). However, with BRPF1 present, $k_{\text{cat}}^{\text{app}}$ for H3.3 (15-36) is approximately 12-fold greater than that for H3 (1-21) ($k_{\text{cat}}^{\text{rel}}$ = 12); this corresponds to a 750-fold (=12/0.016) change in $k_{\text{cat}}^{\text{rel}}$. These results indicate that KAT6A^FL^-BRPF1 complexes alter the intrinsic rate constant for one or more steps following the binding of the H3.3 (15-36) peptide. While our steady-state experiments cannot define reaction steps subsequent to peptide binding, future studies that employ transient state kinetic analysis can dissect these effects and are currently in progress.

BRPF1 can also promote H3K23 acetylation by the KAT6A MYST domain but to a lesser extent compared to KAT6A^FL^ (Fig. 7). For the KAT6A MYST domain, BRPF1 increases (*k*_cat_/*K*_M_)^rel^ by 65-fold ((*k*_cat_/*K*_M_)^rel^ = 0.014 and 0.91 for MYST and the MYST 2-plex, respectively), which is less than the ≈10^3^-fold effect we see with KAT6A^FL^ (Fig. 7*A*). In addition, BRPF1 increases $k_{\text{cat}}^{\text{rel}}$ for the KAT6A MYST by 165-fold ($k_{\text{cat}}^{\text{rel}}$ = 0.014 and 2.3 for MYST and the MYST 2-plex, respectively) while this effect for KAT6A^FL^ is 750-fold (Fig. 7). These differences imply that regions outside of the KAT6A MYST domain cooperate with BRPF1 to facilitate acetylation of H3K23.

KAT6A/B contains the double PHD finger (DPF) domain (Fig. 1), a histone recognition module that binds to the H3 tail as well as H3 tail derivatives that are acylated at H3K14 (27, 30, 49, 50, 51). Isothermal titration calorimetry with the KAT6A DPF domain indicates that this reader domain can bind to unmodified H3 (1-25) and structures of the DPF-domain complexed with K14-acylated H3 (1-25) peptides provide a model for molecular recognition of this peptide (51). While we cannot rule out the possibility that other regions within KAT6A^FL^ interact with the histone H3 tail, the available data for the KAT6A DPF domain provide the simplest explanation for the greater acetyltransferase activity towards H3K23 by KAT6A^FL^ 2-plex compared to the MYST 2-plex. Interactions made between the H3 tail and the DPF domain could help position H3K23 for acetylation by the KAT6A^FL^ when complexed with BRPF1.

Interactions made with the DPF domain can also explain the different acetyltransferase activities of the uncomplexed forms of KAT6A^FL^ and the KAT6A MYST domain. Values of *k*_cat_/*K*_M_ for acetylation of H3.3 (1-43), H3 (1-21), and H3.3 (15-36) are 10- to 40-fold greater for KAT6A^FL^ compared to the MYST domain (Fig. 4*C* and Table 2). In contrast, values of *k*_cat_ are essentially the same for both forms of this enzyme (Table 2). A model for this effect is that the DPF domain stabilizes peptide binding to KAT6A^FL^; such an effect is consistent with the 10- to 40-fold lower values of *K*_M_ for reactions with KAT6A^FL^ compared to the MYST domain (Table 2).

Alternatively, the distinct functional properties of the KAT6A^FL^- and MYST- 2-plexes could arise from differences in complex stability. Deletion mapping studies of KAT6A^FL^ indicate that the MYST domain is necessary and sufficient for binding to BRPF1 (29). However, KAT6A^FL^ is a ≈225 kDa protein composed of DNA and protein recognition modules (Fig. 1), as well as long segments of significant intrinsic disorder (60). It is possible that one or more of these regions enhances the stability of the KAT6A^FL^-BRPF1 complex, thereby reducing the amount of uncomplexed KAT6A^FL^ in acetyltransferase assays. In contrast, reactions with the less stable MYST 2-plex may contain higher levels of uncomplexed MYST domain. Since association with BRPF1 is essential for robust H3K23 acetylation, differences in complex stability could account for the greater acetyltransferase activity of the KAT6A^FL^ 2-plex towards H3K23 compared to the MYST 2-plex.

More broadly, these results underscore how regions outside the catalytic domain can influence the properties of MYST acetyltransferases. While members of the MYST family share a highly conserved catalytic domain, they vary considerably in size (11) as well as in histone recognition domains: KAT6A/B contain the DPF domain (see above), KAT5 and KAT8 contain a chromodomain (61), and KAT7 contains an N-terminal histone binding domain (62). Such variation may guide the acetyltransferase activity of each MYST member toward specific lysine residues through interactions with associated scaffold proteins and/or with the histone tail.

### A glycine pair facilitates H3K14 acetylation by KAT6A

For KAT6A^FL^ and the KAT6A MYST domain, the preferred acetylation of H3K14 over H3K23 is associated with differences in the amino acid sequence of residues proximal to these two lysine substrates (Figs. 2*D* and 4*D*, respectively). Two glycine residues precede H3K14 (G12 and G13) and mutations that alter either one of these residues perturbs acetylation; conversely, alanine and threonine precede H3K23 (A21 and T22) and replacing both residues with glycine stimulates acetylation. Consistent with these results, we also detected acetylation of H4K5, H4K8, H4K12, and H2AK9 in assays with HeLa nucleosomes (Fig. S2, *A* and *B*) and each of these residues are immediately adjacent to a pair of glycine residues.

Glycine is the smallest amino acid and can assume backbone conformational states that are unfavorable for all other amino acids (48). In addition, computational and experimental data suggest that glycine pair motifs confer significant conformational flexibility, especially when next to charged amino acids such as lysine (63). Thus, the simplest interpretation of the above results is that the adjacent glycine pair facilitates access to conformational states that accommodate the neighboring lysine residue into the active site and/or reduce steric interference with neighboring residues.

A crystal structure of the KAT6A MYST domain complexed with a H3K14-CoA bisubstrate inhibitor allows us to consider molecular features that may be relevant to recognition of H3K14 by this enzyme. The MYST domains forms 8 putative hydrogen bonds with the H3 (10-17) moiety and most (7/8) of these interactions are to the peptide backbone; the remaining contact is made with the K14 ϵ-amino group (Fig. 3*C*). The dearth of specific sidechain interactions is consistent with a model in which other factors, such as size and flexibility, guide recognition by the MYST domain. Indeed, the bound peptide is situated within a crevice formed by three loops, α_2_-β_7_, β_8_- β_9_, and α_3_-α_4_, which contain several bulky residues that are in proximity (3.3–4.5 Å to G12 and G13 (Fig. 3*D*). We speculate that the flexibility and small size of glycine facilitates sampling of functional states with minimal steric hindrance from residues within these loops. These functional states may include conformations that permit stable binding and/or acetylation of substrates.

While our data provide strong evidence that a glycine pair facilitates the recognition of adjacent lysine residues by KAT6A, other modes of sequence-specific recognition are possible. For example, in assays where KAT6A^FL^ or the MYST domain were incubated with HeLa nucleosomes (Fig. S2, *A* and *B*), we detected acetylation of H4K16 and H2AK5, which are separated by one residue (alanine and glutamine, respectively) from a glycine pair and another study detected NuA4-catalyzed acetylation of H4K16 within a short peptide (GGAK_16_RHR) (40). Moreover, lysine residues within non-histone proteins (*e.g.* p53, SMAD3) have been implicated as substrates for KAT6A (64, 65) but are not in proximity to a glycine pair. It will thus be of interest to explore the sequence landscape for molecular recognition by MYST enzymes and how this preference is mediated by MYST complexes.

### How does BRPF1 promote H3K23 acetylation by KAT6A?

Our results with H3 (1-21) and H3.3 (15-36) indicate that BRPF1 alters the substrate preference of KAT6A^FL^ towards these peptides by three orders of magnitude (Fig. 7). To learn more about this change in specificity, we asked whether a similar effect is observed with the truncated peptides, H3 (10-17) and H3 (19-26). Compared to H3 (1-21) and H3.3 (15-36), these short peptides are ≈10^3^-fold less efficient substrates for the KAT6A^FL^ 4-plex and 2-plex (Table 3). A portion of this decrease in acetyltransferase activity likely arises from the removal of distal recognition elements between the H3 tail and the KAT6A^FL^-BRPF1 complex.

Histone recognition domains are present in KAT6A^FL^ (*e.g.* the DPF domain, see above) as well as in BRPF1 (Fig. 1 and Table S2), which include the PZP domain, the bromodomain, and the PWWP domain. The BRPF1 PZP domain can bind to unmodified forms of the histone H3 tail while the bromodomain and PWWP domain have been shown to bind to H3 tail peptides that bare acetyllysine and trimethylated K36, respectively (66, 67, 68). It is possible that one or more of these modules interact with the histone H3 tail to stabilize binding to the KAT6A^FL^ 2- and 4-plexes; this model is consistent with the increase in *K*_M_ (≥1000 μM, Table 3) observed for H3 (10-17) and H3 (19-26) compared to their longer counterparts.

Despite the substantial decrease in activity, we observe that the KAT6A^FL^ 2- and 4-plexes favor acetylation of the H3 (19-26) peptide over H3 (10-17) by approximately 4-fold (Figs. 5*C* and 6*D*). This contrasts with uncomplexed KAT6A^FL^, where *k*_cat_/K_M_ for H3 (19-26) is 40-fold lower than that for H3 (10-17) (Fig. 4*C*). Therefore, even with these truncated peptides, KAT6A^FL^-BRPF1 complexes shift the substrate preference of KAT6A^FL^ by 160-fold (Fig. S9). These results imply changes in molecular recognition of H3 (10-17) and H3 (19-26) by KAT6A^FL^ when bound to BRPF1.

The α_2_-β_7_, β_8_- β_9_, and α_3_-α_4_ loops of the MYST domain flank the peptide binding site (Fig. 3*B*). For KAT6A^FL^ and the MYST domain, these loops are presumably arranged to favor the recognition of K14 (which follows G12 and G13) over K23 (which follows A21 and T22). In the presence of BRPF1, it is possible that the α_2_-β_7_, β_8_- β_9_, and/or α_3_-α_4_ loops undergo a rearrangement to accommodate K23 of H3 (19-26) while minimizing steric hindrance with A21 and T22. This model is supported by prior evidence for conformational changes in MYST acetyltransferases. For example, structural studies of the Esa1 MYST domain show that α_2_-β_7_ is repositioned following the formation of the NuA4 complex, with this change proposed to alter substrate recognition (40). In addition, functional and structural analyses of K274 mutations within α_2_-β_7_ of the KAT8 MYST domain link alterations in the conformation of this loop with changes in acetyltransferase activity (13, 43, 69).

The preferred acetyltransferase activity of the KAT6A^FL^ 4-plex towards H3 (19-26) is lost when the alanine and/or threonine residues that precede K23 (*i.e.* A21 and T22) are replaced with glycine (Fig. 5*D*). Notably, we observe a substantial (approximately 20-fold) decrease in activity when T22 is replaced with glycine. It is possible that the KAT6A^FL^ 2-plex forms a hydrogen bond with the sidechain hydroxyl group of T22 and that this interaction facilitates the accommodation of the adjacent K23 residue into the active site. Evaluating these proposed modes of H3 tail recognition by KAT6A^FL^ 2-plex would be aided by high-resolution crystal and/or cryo-EM structures of this complex, which are currently unavailable.

KAT6A^FL^ and BRPF1 are large proteins with substantial unstructured regions (60), properties that complicate the acquisition of structural data for KAT6A^FL^ alone and the KAT6A^FL^ 2-plex. One approach to address this challenge would be to generate fragments of KAT6A^FL^ and BRPF1, as early studies have done to investigate their binding interface (29). The reduced size of these fragments can improve protein yield and purity, facilitate structural studies, and enable the use of transient kinetic methods for deeper analysis. Efforts to generate these fragments and characterize them using a combination of biochemical and structural techniques are in progress.

### Modulating the substrate preference of MYST acetyltransferases

The substrate preference of KAT6A and its modulation by BRPF1 have implications for understanding the specificity of MYST complexes. The KAT6A MYST domain acetylates lysine residues in histones H3 (H3K14), H4 (H4K5, H4K8, H4K12, and H4K16), and H2A (K5 and K9) (Figs. 2*A* and S2*A*). A similar profile has been observed for the KAT5 MYST domain (39). Given that MYST members share a highly conserved catalytic module (12, 13), it is likely that the acetyltransferase domains of the remaining MYSTs (KAT6B, KAT7, and KAT8) exhibit enzymatic activity towards all these substrates. Nevertheless, MYST members modify distinct lysine residues in cells and this specificity, as shown here and in other studies, is directed by the distinct complexes formed by each enzyme (Table S1 and references therein).

MYST complexes can mediate histone tail selection; for example, KAT7-BRPF(2/3) complexes modify histone H3 (H3K14) whereas KAT7-JADE(1/2/3) complexes modify histone H4 (H4K5, H4K8, and H4K12) (Table S1). Our studies also highlight the ability of MYST complexes to distinguish between lysine residues within the same histone tail, as shown by the change in substrate preference of KAT6A^FL^ from H3K14 to H3K23 when bound to BRPF1. This observation underscores nature’s ability to confer MYST complexes with sufficient specificity to discriminate between lysine residues separated by eight amino acids.

BRPF1 can promote H3K23 acetylation by KAT6A but this property is not shared by the two paralogs of this scaffold protein, BRPF2 and BRPF3, which bind to KAT7. Although these three proteins are highly conserved, KAT7-BRPF2 and KAT7-BRPF3 complexes acetylate H3K14 (17, 18) whereas the noncanonical KAT7-BRPF1 complex acetylates H3K23 (18, 52). It will be of interest to understand how complex formation with these closely related scaffold proteins give rise to these distinct histone products. Identifying and defining these mechanisms will facilitate our understanding of transcriptional regulation by MYST members and their therapeutic targeting.

## Experimental procedures

### Materials

Acetyl-CoA (≥93%) was purchased from Sigma-Aldrich. Histone H3 peptides (90–98%) were purchased from Biosynth, CPC Scientific, or Genscript with amino acid analysis provided to determine the concentration of the peptides. All other reagents, unless otherwise states, were obtained from Sigma-Aldrich or Fisher Scientific.

### Preparation of HeLa oligonucleosomes

HeLa oligonucleosomes were prepared according to reported procedures (70, 71). A 10 L culture of HeLa S3 cells (Kemp Biotechnologies) was grown to a density of 4e^6^/ml in S3 medium and cell pellets were subsequently harvested by centrifugation and frozen at −80 °C. Nuclei were obtained by hypotonic lysis in 160 ml of lysis buffer (20 mM HEPES pH 7.5, 0.25 M Sucrose, 3 mM MgCl_2_, 0.5% Nonidet P-40, 0.5 mM TCEP, and 0.2 mM PMSF) followed by centrifugation (3000 g, 15 min). This procedure was repeated twice followed by two wash/spin steps, the first with buffer B (160 ml, 20 mM HEPES pH 7.5, 3 mM MgCl_2_, 0.5 mM EDTA, 0.5 mM TCEP, and 0.2 mM PMSF) and the second with medium-salt buffer (160 ml, 20 mM HEPES pH 7.5, 0.4 M NaCl, 1 mM EDTA, 5% v/v Glycerol, 0.5 mM TCEP, 0.2 mM PMSF).

The nuclei pellet was resuspended in 60 ml of high-salt buffer (20 mM HEPES pH 7.5, 0.65 M NaCl, 1 mM EDTA, 0.34 M Sucrose, 0.5 mM TCEP, 0.2 mM PMSF), sheared *via* dounce homogenization, and dialyzed overnight against low-salt buffer (20 mM HEPES pH 7.5, 50 mM NaCl, 1 mM EDTA, 0.5 mM TCEP, 0.2 mM PMSF). CaCl_2_ was added to a final concentration of 3 mM and the mixture was preheated at 37 °C for 5 min. The nuclei were then digested with Monococcal nuclease (Worthington Biochemical) at 50 U/ml for 12.5 min. The reaction was quenched by adding EDTA (6 mM) and placing the mixture on ice. NaCl was subsequently added dropwise to a final concentration of 0.65 M.

Nucleosomes were purified through a sucrose gradient (5–35% over 34 ml) in sucrose gradient buffer (20 mM HEPES pH 7.5, 1 mM EDTA, 0.65 M NaCl, 0.2 mM PMSF, 0.5 mM TCEP) using AKTA pure (Cytiva) in a 38.5 ml tube (Beckman Coulter). 4 ml of digested nuclei were loaded on sucrose gradients which were centrifuged in a SW28 rotor at 26,000 RPM for 16 h at 4 °C using an Optima LE-80K centrifuge (Beckman Coulter). The SW28 rotor holds 6 buckets, and 5 runs were performed to purify all nucleosomes. 2 ml fractions were collected from the top of each tube and were subsequently evaluated for DNA and histone proteins by agarose and polyacrylamide gel electrophoresis, respectively. Fractions enriched with nucleosomes were pooled and dialyzed against 16 L of low-salt buffer. Nucleosomes were concentrated in a stirred cell (Amicon) to a final concentration of 2 mg/ml (*via* Bradford assay) before 10% glycerol was added. Nucleosomes were aliquoted and flash frozen in liquid N_2_ and stored at −80 °C until further use.

### Protein expression and purification

The KAT6A MYST domain (KAT6A 501–784), KAT6A^FL^, and the KAT6A^FL^ 4-plex were expressed and purified as previously described (6). In brief, human *KAT6A* (NCBI Reference Sequence: NP_006757.2) or human *BRPF1* (NCBI Reference Sequence: NP_004625.2) were synthesized *de novo* and subcloned into a modified pFastBac1 (Invitrogen) vector. For expression of BRPF1-ING5-MEAF6, *BRPF1* (NCBI Reference Sequence: NP_004625.2), *ING5* (NCBI Reference Sequence: NP_115705.2), and *MEAF6* (NCBI Reference Sequence: NP_001257804.1) were subcloned into a modified pFastBacDual vector (Invitrogen).

To facilitate recombinant protein expression, two to 16 L culture of Sf21 insect cells at a density of 2e^6^/ml was infected with equal amounts of high-titer stock of recombinant baculovirus generated from transfer vectors that include pHis-MYST domain; Flag-KAT6A^FL^; Flag-KAT6A^FL^ with BRPF1-ING5- MEAF6; Flag-KAT6A^FL^ with BRPF1; and Flag-MYST with BRPF1. Infected cells were grown in shaker flasks for 72 h at 27 °C. Cell pellets were then harvested by centrifugation, and frozen at −80 °C for at least 24 h prior to purification. All purification steps were performed at 4 °C. Frozen cell pellets were resuspended in 3 ml/g of lysis buffer containing 50 mM Tris pH 8.0, 250 mM NaCl, 0.25 mM TCEP, 1/75 ml Roche EDTA-free protease inhibitor tablets, 10 μM leupeptin, and 10 μM E−64 protease inhibitor, then clarified by centrifugation.

Following reported procedures (6), pHis-MYST domain was purified *via* nickel affinity capture, with the N-terminal polyhistidine tag subsequently cleaved by TEV protease (Fig. S10*A*). The sample was then passed through the nickel affinity resin and flow-through fractions containing the protein were collected (Fig. S10*A*). KAT6A^FL^, KAT6A^FL^ 4-plex, KAT6A^FL^ 2-plex, and MYST 2-plex were purified using anti-Flag resin (Pierce) (Fig. S10, *B*–*E*). All protein samples were concentrated and passed over a gel filtration chromatography column (SRT-500, Sepax Technologies) equilibrated in 25 mM HEPES pH 7.5, 250 mM NaCl, and 0.25 mM TCEP. Peak fractions were pooled and concentrated to 1 to 5 mg/ml, as determined by the Bradford assay, and analyzed on analytical reducing SDS-PAGE gels stained with Coomassie blue (Fig. S1). Aliquots of the protein samples were flash-frozen and stored at −80 °C until further use.

While the MYST domain was purified to homogeneity, all other forms of KAT6A were partially purified (Fig. S1). KAT6A^FL^ (≈225 kDa) and BRPF1 (≈137 kDa) are large proteins with significant unstructured regions (60), and efforts to improve the purity of these proteins were hindered by poor expression and/or limited protein solubility. To determine the amount of active enzyme in each form of KAT6A, we employed active site titration *via* tight binding inhibition by PF-9363, as described below.

### General reaction conditions

All reactions were multiple-turnover, with substrates in excess (≥10-fold) of enzyme and, unless otherwise stated, were measured at 25 °C in the presence of 50 mM HEPES (pH 7.5), 5 mM NaCl, 0.1 mM EDTA, and 0.002% Tween-20. Enzyme and peptide were allowed to incubate at 25 °C for 10 min before initiating the reaction by adding AcCoA (20 μM). At various times, eight 2 μl aliquots were collected and transferred to 50 μl of 1% formic acid to quench the reaction. Acetyl-CoA and CoA were subsequently detected using a RapidFire 365 high-throughput SPE chromatography system coupled to a 6495 triple quadrupole mass spectrometer (Agilent Technologies) based on procedures described previously (72, 73). 42 uL of reaction timepoints were injected onto an Agilent C4 cartridge (40 μm, type A2) in 6 mM octylammonium acetate (GLSynthesis) and eluted with 25% acetonitrile, 25% acetone, and 50% 6 mM octylammonium acetate. RapidFire settings were as follows: aspiration time: 600 ms, load time: 4500 ms, elution time: 8000 ms, and re-equilibration time: 500 ms at a flow rate of 0.8 ml/min.

Samples were eluted into an Agilent 6495 triple quadrupole mass spectrometer with an Agilent Jet Stream source with ion funnel technology, set in the positive ion mode. A multiple reaction monitoring (MRM) protocol was optimized employing Q1 *m/z* ratios of 810.1 and 768.05 for acetyl-CoA and CoA, respectively. A second quadruple (Q2) was determined, using house nitrogen as the collision gas (fragmentor voltage = 380 V, collision energy = 30 V) with *m/z* values of 303.1 and 261.0 for acetyl-CoA and CoA, respectively.

Area under the curve (AUC) for acetyl-CoA and CoA were quantitated using RapidFire Integrator software (Agilent). The concentration of CoA was calculated from fraction (CoA) (AUC^CoA^/(AUC^Acetyl-CoA^ + AUC^CoA^)) multiplied by the concentration of acetyl-CoA used in acetyltransferase assays.

### Active site titration

The active enzyme concentration for each form of KAT6A was obtained through tight-binding inhibition (48) by PF-9363, which binds to the KAT6A MYST domain with an inhibition constant (*K*_i_) of ≈0.3 nM (6). Each form of KAT6A was incubated with acetyl-CoA (1–2 μM) and various concentrations of PF-9363 (0–1000 nM) for 15 to 30 min prior to initiating the reaction with H3 (1-21) (5–10 μM). The initial velocity measured at a given concentration of PF-9363 (*V*_i_) was normalized with respect to the initial velocity in the absence of inhibitor (*V*_0_) and fit to Equation 1:

(1) $$V_{i}/V_{0}=1\;-\;\frac{\left({\left[E\right]}_{T}+\left[I\right]+K_{i,\text{app}}\;\right)\;–\sqrt{{\left({\left[E\right]}_{T}+\left[I\right]+K_{i,\text{app}}\right)}^{2}-\;4{\left[E\right]}_{T}\left[I\right]}}{2{\left[E\right]}_{T}}$$

where *K*_i,app_ is the apparent inhibition constant of PF-9363 and [E]_T_ is the active enzyme concentration. The subscript “app” is included in *K*_i,app_ since acetyl-CoA competes with PF-9363 for binding to the MYST domain (6). In control experiments, varying the concentration of each form of KAT6A was shown to shift the inhibition curve, consistent with tight binding by PF-9363 (Fig. S11) (58). In addition, varying the incubation time for PF-9363 binding to each form of KAT6A (15–30 min) did not alter the time-dependence of the inhibition curve, suggesting that the reaction is at equilibrium (data not shown) (58).

### Histone proteomics

Acetyltransferase assays with HeLa nucleosomes were performed at room temperature in the presence of 10 to 25 mM Tris-HCl (pH 8.0), 180 mM NaCl, 0.5 mM EDTA, 0.4 to 1 μM HeLa oligonucleosomes, 10 μM acetyl-CoA, and 25 to 250 nM enzyme. For reactions with the KAT6A MYST domain, we observed inhibition by NaCl (data not shown) and thus performed reactions with HeLa oligonucleosomes in a background of 5 mM NaCl. For acetylation of H3.3 (1-43) by the KAT6A MYST domain, the reaction was performed at room temperature with 50 mM HEPES (pH 7.5), 5 mM NaCl, 0.1 mM EDTA, 50 μM H3.3 (1-43), 50 μM acetyl-CoA, and 600 nM KAT6A MYST domain. Acetylation was followed at various times before being quenched in 1% formic acid for subsequent MS analysis.

Proteomics services were performed by the Northwestern Proteomics Core Facility (https://proteomics.northwestern.edu/). In brief, histones were extracted from quenched samples *via* precipitation with trichloroacetic acid, derivatized with propionic anhydride, and digested with trypsin (37). For reactions with H3.3 (1-43), the same procedure was followed but the histone extraction step was omitted. This workflow generates peptides of known sequence that can be analyzed using selective reaction monitoring (SRM) LC-MS/MS, with three technical replicates per sample (74). AUC values were obtained for unmodified (AUC^unmodified^) and acetylated (AUC^acetylated)^ forms of each interrogated lysine residue and the percent acetylation was calculated from AUC^acetylated^/(AUC^unmodified^ + AUC^acetylated^).

### Steady state kinetic measurements

Reactions were performed under multiple-turnover conditions with excess AcCoA (20 μM) and peptide (1–3000 μM) relative to enzyme (10–100 nM). Reactions were followed for up to 20% product formation and linear fits of the data provided initial velocities (*V*), which were divided by the enzyme concentration ([E]_T_) determined from active site titration. *V*/[E]_T_ was plotted as a function of [peptide] and fit by the Michaelis-Menten equation (Equation 2):

(2) $$V/{\left[E\right]}_{T}=k_{\text{cat}}\left[\text{peptide}\right]/\;\left(K_{M}+\left[\text{peptide}\right]\right)$$

where *k*_cat_ is the maximum rate of turnover and *K*_M_ is the Michaelis-constant. To determine *k*_cat_/*K*_M_, the steady-state data were fit to a modified form of the Michaelis-Menten equation (Equation 3) (58):

(3) $$V/{\left[E\right]}_{T}=\left(k_{\text{cat}}/K_{M}\right)\left[\text{peptide}\right]/\;\left(1+\left(k_{\text{cat}}/K_{M}\right)\left[\text{peptide}\right]/k_{\text{cat}}\right)$$

For H3 (10-17), H3 (19-26), and their chimeric peptides, exceedingly high peptide concentrations were needed to saturate acetyltransferase activity. The steady-state data were instead fit to a linear equation to obtain *k*_cat_/*K*_M_. Finally, for the KAT6A^FL^ 2- and 4-plexes as well as the MYST 2-plex, steady-state parameters include a superscript “app” to denote that they are apparent values since reactions with these forms of KAT6A may include unbound KAT6A^FL^ or MYST.

### Determination of the structure of KAT6A MYST complexed with the H3K14-CoA bisubstrate inhibitor

The H3K14-CoA inhibitor scaffold was synthesized *via* manual peptide synthesis using an orthogonally protected Fmoc-Lys(ivDde)-OH protecting group similar to the previous protocol (44, 75). Briefly, a peptide with the linear sequence QTARKSTGGK(ivDde)APRKQLATK was synthesized on Nova PEG Rink Amide using standard Fmoc chemistry. After the final amino acid was incorporated, Fmoc-Ahx-OH was coupled to the N-terminus and the ivDde group was removed using hydrazine. Bromoacetic anhydride was then coupled to the ε-amine, followed by resin cleavage and HPLC purification. This purified bromoacetamide peptide was then dissolved in 100 mM pH 8 NaHCO_3_ and Coenzyme A trilithium salt was added. The solution was stirred for 30 min at room temperature, diluted with aqueous 0.1% TFA (5 ml), and 1 M HCl was added dropwise to adjust the solution to pH 2 prior to preparative HPLC purification. The purified peptidyl-CoA analogue was quantified using the molar extinction coefficient (ε) for Coenzyme A of 15,000 M^−1^ cm^−1^ at λ_max_ of 259 nm (76).

For crystallization, KAT6A MYST domain at 8 mg/ml was complexed with H3K14-CoA in a 1:10 M ratio. The complex was incubated for 4 h at 4 °C and passed through a 0.45-μm cellulose-acetate filter. Using 96-well MRC-2 sitting-drop plates with solutions dispensed *via* Mosquito liquid handling (TTP Biotech), the complex was set up at a 1:1 ratio (300 nl) with a growth solution consisting of 1.25 M NH_4_SO_4_, 0.28 M NaCl, and 0.1 M HEPES pH 6.4, and allowed to incubate at 21 °C. Crystals began growing within 24 h and reached full size (approximately 0.2 × 0.15 × 0.15 μm) after 4 days. Crystals were dipped into growth solution containing 20% glycerol and subsequently frozen in liquid nitrogen.

X-ray diffraction data were collected at the Advanced Photon Source beamline 17-ID and processed with autoPROC (77) using STARANISO (78) from Global Phasing. Structure solution and refinement were done using BUSTER (79), model building was done in COOT (77, 80) and figures were generated using PYMOL.

## Data availability

RCSB Protein Data Bank Accession code: The coordinates and structure factors for the KAT6A MYST domain complexed with the H3K14-CoA bisubstrate inhibitor have been deposited in the RCSB Protein Data Bank under accession code 9DZN.

## Supporting information

This article contains supporting information (6, 9, 14, 15, 16, 17, 18, 19, 20, 21, 22, 23, 24, 25, 27, 28, 29, 30, 40, 42, 49, 50, 51, 52, 55, 56, 57, 66, 67, 68, 82, 83, 84, 85, 86, 87, 88, 89, 90, 91, 92, 93, 94, 95, 96, 97).

## Conflict of interest

The authors declare that they have no conflicts of interest with the contents of this article.

## Footnotes

1 “KAT6A/B” refers to both KAT6A and KAT6B.

## Footnotes

2 HeLa oligonucleosomes are known to carry preexisting posttranslational modifications at lysine residues within the histone tails (81). To account for this, our experiments consistently include a 0-timepoint, which shows minimal modification at H3K9, H3K14, H3K23, and H3K27. Furthermore, the 0-timepoint serves as a baseline, enabling us to assess changes in the modification state of specific lysine residues relative to this starting point.

3 The three major variants of the H3 protein are H3.1, H3.2, and H3.3, which differ by one amino acid within the N-terminal histone tail; H3.1 and H3.2 contain alanine at residue 31 while H3.3 contains a serine at this position (81). Building on prior work (6) that suggested differences in the substrate preference of KAT6A^FL^ and the KAT6A^FL^ 4-plex using H3.3 (1-43), we sought to further investigate these differences in the current study using the H3.3 (1-43) and H3.3 (15-36) peptides. Additionally, steady-state experiments with H3.1 (15-36) and H3.3 (15-36) demonstrate that replacing serine at position 31 with alanine does not alter the acetyltransferase activity of the KAT6A forms (data not shown), implying that the choice of H3 tail variant (H3.1, H3.2, or H3.3) does not affect substrate preference.

4 We also monitored acetylation of H3.3 (1-43) by the KAT6A^FL^ and MYST 2-plexes (Fig. S5, *D* and *E*, respectively). For both 2-plexes as well as the KAT6A^FL^ 4-plex, (*k*_cat_/*K*_M_)^app^ was greater for H3.3 (1-43) compared to the shorter peptides (Table 3). This enhancement presumably arises from additional interactions made between the longer H3.3 (1-43) peptide and BRPF1 (*e.g.* the PZP domain, (82)).

5 The magnitude of the change in (*k*_cat_/*K*_M_)^rel^ and $k_{\text{cat}}^{\text{rel}}$ could be greater, as reactions involving the KAT6A complexes may include uncomplexed forms of KAT6A^FL^ or the MYST domain, which do not readily modify H3K23.

## References

[1] Zhao S., Allis C.D., Wang G.G. (2021). The language of chromatin modification in human cancers. Nat. Rev. Cancer.

[2] Feehley T., O'Donnell C.W., Mendlein J., Karande M., McCauley T. (2023). Drugging the epigenome in the age of precision medicine. Clin. Epigenetics.

[3] Nepali K., Liou J.P. (2021). Recent developments in epigenetic cancer therapeutics: clinical advancement and emerging trends. J. Biomed. Sci..

[4] Whedon S.D., Cole P.A. (2023). KATs off: biomedical insights from lysine acetyltransferase inhibitors. Curr. Opin. Chem. Biol..

[5] White J., Derheimer F.A., Jensen-Pergakes K., O'Connell S., Sharma S., Spiegel N. (2024). Histone lysine acetyltransferase inhibitors: an emerging class of drugs for cancer therapy. Trends Pharmacol. Sci..

[6] Sharma S., Chung C.Y., Uryu S., Petrovic J., Cao J., Rickard A. (2023). Discovery of a highly potent, selective, orally bioavailable inhibitor of KAT6A/B histone acetyltransferases with efficacy against KAT6A-high ER+ breast cancer. Cell Chem. Biol..

[7] Huan F., Abmayr S.M., Workman J.L. (2016). Regulation of KAT6 acetyltransferases and their roles in cell cycle progression, stem cell maintenance, and human disease. Mol. Cell Biol..

[8] Viita T., Côté J. (2022). The MOZ-BRPF1 acetyltransferase complex in epigenetic crosstalk linked to gene regulation, development, and human diseases. Front. Cell Dev. Biol..

[9] Yang X.J. (2015). MOZ and MORF acetyltransferases: molecular interaction, animal development and human disease. Biochim. Biophys. Acta.

[10] Mukohara T., Park Y.H., Sommerhalder D., Yonemori K., Hamilton E., Kim S.B. (2024). Inhibition of lysine acetyltransferase KAT6 in ER(+)HER2(-) metastatic breast cancer: a phase 1 trial. Nat. Med..

[11] Avvakumov N.C.,J., Kundu T.D.,D. (2007). Chromatin and Disease.

[12] Yan Y., Barlev N.A., Haley R.H., Berger S.L., Marmorstein R. (2000). Crystal structure of yeast Esa1 suggests a unified mechanism for catalysis and substrate binding by histone acetyltransferases. Mol. Cell.

[13] Yuan H., Rossetto D., Mellert H., Dang W., Srinivasan M., Johnson J. (2012). MYST protein acetyltransferase activity requires active site lysine autoacetylation. EMBO J..

[14] Wichmann J., Pitt C., Eccles S., Garnham A.L., Li-Wai-Suen C.S.N., May R. (2022). Loss of TIP60 (KAT5) abolishes H2AZ lysine 7 acetylation and causes p53, INK4A, and ARF-independent cell cycle arrest. Cell Death Dis..

[15] Yan K., Rousseau J., Machol K., Cross L.A., Agre K.E., Gibson C.F. (2020). Deficient histone H3 propionylation by BRPF1-KAT6 complexes in neurodevelopmental disorders and cancer. Sci. Adv..

[16] Kueh A.J., Dixon M.P., Voss A.K., Thomas T. (2011). HBO1 is required for H3K14 acetylation and normal transcriptional activity during embryonic development. Mol. Cell Biol..

[17] Mishima Y., Miyagi S., Saraya A., Negishi M., Endoh M., Endo T.A. (2011). The Hbo1-Brd1/Brpf2 complex is responsible for global acetylation of H3K14 and required for fetal liver erythropoiesis. Blood.

[18] Feng Y.P., Vlassis A., Roques C., Lalonde M.E., González-Aguilera C., Lambert J.P. (2016). BRPF3-HBO1 regulates replication origin activation and histone H3K14 acetylation. Embo J..

[19] Foy R.L., Song I.Y., Chitalia V.C., Cohen H.T., Saksouk N., Cayrou C. (2008). Role of Jade-1 in the histone acetyltransferase (HAT) HBO1 complex. J. Biol. Chem..

[20] Miotto B., Struhl K. (2010). HBO1 histone acetylase activity is essential for DNA replication licensing and inhibited by geminin. Mol. Cell.

[21] Taipale M., Rea S., Richter K., Vilar A., Lichter P., Imhof A. (2005). hMOF histone acetyltransferase is required for histone H4 lysine 16 acetylation in mammalian cells. Mol. Cell Biol..

[22] Smith E.R., Cayrou C., Huang R., Lane W.S., Côté J., Lucchesi J.C. (2005). A human protein complex homologous to the MSL complex is responsible for the majority of histone H4 acetylation at lysine 16. Mol. Cell Biol..

[23] Thomas T., Dixon M.P., Kueh A.J., Voss A.K. (2008). Mof (MYST1 or KAT8) is essential for progression of embryonic development past the blastocyst stage and required for normal chromatin architecture. Mol. Cell Biol..

[24] Zhao X.M., Su J.M., Wang F., Liu D., Ding J., Yang Y. (2013). Crosstalk between NSL histone acetyltransferase and MLL/SET complexes: NSL complex functions in promoting histone H3K4 Di-methylation activity by MLL/SET complexes. PLoS Genet..

[25] Radzisheuskaya A., Shliaha P.V., Grinev V.V., Shlyueva D., Damhofer H., Koche R. (2021). Complex-dependent histone acetyltransferase activity of KAT8 determines its role in transcription and cellular homeostasis. Mol. Cell.

[26] Avvakumov N., Côté J. (2007). The MYST family of histone acetyltransferases and their intimate links to cancer. Oncogene.

[27] Klein B.J., Jang S.M., Lachance C., Mi W., Lyu J., Sakuraba S. (2019). Histone H3K23-specific acetylation by MORF is coupled to H3K14 acylation. Nat. Commun..

[28] Kitabayashi I., Aikawa Y., Nguyen L.A., Yokoyama A., Ohki M. (2001). Activation of AML1-mediated transcription by MOZ and inhibition by the MOZ-CBP fusion protein. EMBO J..

[29] Ullah M., Pelletier N., Xiao L., Zhao S.P., Wang K., Degerny C. (2008). Molecular architecture of quartet MOZ/MORF histone acetyltransferase complexes. Mol. Cell Biol..

[30] Dreveny I., Deeves S.E., Fulton J., Yue B., Messmer M., Bhattacharya A. (2014). The double PHD finger domain of MOZ/MYST3 induces alpha-helical structure of the histone H3 tail to facilitate acetylation and methylation sampling and modification. Nucleic Acids Res..

[31] Voss A.K., Collin C., Dixon M.P., Thomas T. (2009). Moz and retinoic acid coordinately regulate H3K9 acetylation, Hox gene expression, and segment identity. Dev. Cell.

[32] Voss A.K., Vanyai H.K., Collin C., Dixon M.P., McLennan T.J., Sheikh B.N. (2012). MOZ regulates the Tbx1 locus, and Moz mutation partially phenocopies DiGeorge syndrome. Dev. Cell.

[33] Sheikh B.N., Phipson B., El-Saafin F., Vanyai H.K., Downer N.L., Bird M.J. (2015). MOZ (MYST3, KAT6A) inhibits senescence via the INK4A-ARF pathway. Oncogene.

[34] Fei D., Wang Y., Zhai Q., Zhang X., Zhang Y., Wang Y. (2021). KAT6A regulates stemness of aging bone marrow-derived mesenchymal stem cells through Nrf2/ARE signaling pathway. Stem Cell Res. Ther..

[35] Lv D., Jia F., Hou Y., Sang Y., Alvarez A.A., Zhang W. (2017). Histone acetyltransferase KAT6A upregulates PI3K/AKT signaling through TRIM24 binding. Cancer Res..

[36] Yan F., Li J., Milosevic J., Petroni R., Liu S., Shi Z. (2022). KAT6A and ENL form an epigenetic transcriptional control module to drive critical leukemogenic gene-expression programs. Cancer Discov..

[37] Garcia B.A., Mollah S., Ueberheide B.M., Busby S.A., Muratore T.L., Shabanowitz J. (2007). Chemical derivatization of histones for facilitated analysis by mass spectrometry. Nat. Protoc..

[38] Kuo Y.M., Henry R.A., Andrews A.J. (2014). A quantitative multiplexed mass spectrometry assay for studying the kinetic of residue-specific histone acetylation. Methods.

[39] Kimura A., Horikoshi M. (1998). Tip60 acetylates six lysines of a specific class in core histones. Genes Cells.

[40] Xu P., Li C., Chen Z., Jiang S., Fan S., Wang J. (2016). The NuA4 core complex acetylates nucleosomal histone H4 through a double recognition mechanism. Mol. Cell.

[41] Huang H., Sabari B.R., Garcia B.A., Allis C.D., Zhao Y.M. (2014). SnapShot: histone modifications. Cell.

[42] Holbert M.A., Sikorski T., Carten J., Snowflack D., Hodawadekar S., Marmorstein R. (2007). The human monocytic leukemia zinc finger histone acetyltransferase domain contains DNA-binding activity implicated in chromatin targeting. J. Biol. Chem..

[43] McCullough C.E., Song S., Shin M.H., Johnson F.B., Marmorstein R. (2016). Structural and functional role of acetyltransferase hMOF K274 autoacetylation. J. Biol. Chem..

[44] Montgomery D.C., Sorum A.W., Meier J.L. (2014). Chemoproteomic profiling of lysine acetyltransferases highlights an expanded landscape of catalytic acetylation. J. Am. Chem. Soc..

[45] Marmorstein R., Zhou M.M. (2014). Writers and readers of histone acetylation: structure, mechanism, and inhibition. Cold Spring Harb. Perspect. Biol..

[46] Wilmot C.M., Thornton J.M. (1988). Analysis and prediction of the different types of beta-turn in proteins. J. Mol. Biol..

[47] de Brevern A.G. (2016). Extension of the classical classification of β-turns. Sci. Rep..

[48] Copeland R.A. (2000).

[49] Ali M., Yan K.Z., Lalonde M.E., Degerny C., Rothbart S.B., Strahl B.D. (2012). Tandem PHD fingers of MORF/MOZ acetyltransferases display selectivity for acetylated histone H3 and are required for the association with chromatin. J. Mol. Biol..

[50] Qiu Y., Liu L., Zhao C., Han C., Li F., Zhang J. (2012). Combinatorial readout of unmodified H3R2 and acetylated H3K14 by the tandem PHD finger of MOZ reveals a regulatory mechanism for HOXA9 transcription. Genes Dev..

[51] Xiong X., Panchenko T., Yang S., Zhao S., Yan P., Zhang W. (2016). Selective recognition of histone crotonylation by double PHD fingers of MOZ and DPF2. Nat. Chem. Biol..

[52] Lalonde M.E., Avvakumov N., Glass K.C., Joncas F.H., Saksouk N., Holliday M. (2013). Exchange of associated factors directs a switch in HBO1 acetyltransferase histone tail specificity. Genes Dev..

[53] Panchenko M.V. (2016). Structure, function and regulation of jade family PHD finger 1 (JADE1). Gene.

[54] Rothbart S.B., Dickson B.M., Raab J.R., Grzybowski A.T., Krajewski K., Guo A.H. (2015). An interactive database for the assessment of histone antibody specificity. Mol. Cell.

[55] Yan K.Z., You L.Y., Degerny C., Ghorbani M., Liu X., Chen L.L. (2016). The chromatin regulator BRPF3 preferentially activates the HBO1 acetyltransferase but is dispensable for mouse development and survival. J. Biol. Chem..

[56] You L.Y., Li L., Zou J.F., Yan K.Z., Belle J., Nijnik A. (2016). BRPF1 is essential for development of fetal hematopoietic stem cells. J. Clin. Invest..

[57] Yan K.Z., Rousseau J., Littlejohn R.O., Kiss C., Lehman A., Rosenfeld J.A. (2017). Mutations in the chromatin regulator gene *BRPF1* cause syndromic intellectual disability and deficient histone acetylation. Am. J. Hum. Genet..

[58] Johnson K.A. (2019).

[59] Fersht A. (2017).

[60] The UniProt Consortium (2024). UniProt: the universal protein knowledgebase in 2025. Nucleic Acids Res..

[61] Sun X., P S., Mills A.A., Binda O. (2024). Chromatin Readers in Health and Disease.

[62] Han J., Lachance C., Ricketts M.D., McCullough C.E., Gerace M., Black B.E. (2018). The scaffolding protein JADE1 physically links the acetyltransferase subunit HBO1 with its histone H3-H4 substrate. J. Biol. Chem..

[63] An B., Chang S.W., Hoop C., Baum J., Buehler M.J., Kaplan D.L. (2017). Structural insights into the Glycine pair motifs in type III collagen. ACS Biomater. Sci. Eng..

[64] Rokudai S., Laptenko O., Arnal S.M., Taya Y., Kitabayashi I., Prives C. (2013). MOZ increases p53 acetylation and premature senescence through its complex formation with PML. Proc. Natl. Acad. Sci. U. S. A..

[65] Yu B., Luo F., Sun B., Liu W., Shi Q., Cheng S.Y. (2021). KAT6A acetylation of SMAD3 regulates myeloid-derived suppressor cell recruitment, metastasis, and immunotherapy in triple-negative breast cancer. Adv. Sci. (Weinh).

[66] Vezzoli A., Bonadies N., Allen M.D., Freund S.M., Santiveri C.M., Kvinlaug B.T. (2010). Molecular basis of histone H3K36me3 recognition by the PWWP domain of BRPF1. Nat. Struct. Mol. Biol..

[67] Poplawski A., Hu K.F., Lee W., Natesan S., Peng D.N., Carlson S. (2014). Molecular insights into the recognition of N-terminal histone modifications by the BRPF1 bromodomain. J. Mol. Biol..

[68] Klein B.J., Cox K.L., Jang S.M., Côté J., Poirier M.G., Kutateladze T.G. (2020). Molecular basis for the PZP domain of BRPF1 association with chromatin. Structure.

[69] Yang C., Wu J., Sinha S.H., Neveu J.M., Zheng Y.G. (2012). Autoacetylation of the MYST lysine acetyltransferase MOF protein. J. Biol. Chem..

[70] Fang J., Wang H., Zhang Y. (2004). Purification of histone methyltransferases from HeLa cells. Methods Enzymol..

[71] Schnitzler G.R. (2001). Isolation of histones and nucleosome cores from mammalian cells. Curr. Protoc. Mol. Biol. Chapter.

[72] Bingham P.J., Maegley K., Krivacic C.T. (2014). RapidFire MS/MS enables both rapid evaluation of multiple histone methyltransferases and label-free high throughput screening of targeted compound libraries. Cancer Res..

[73] Rye P.T., Frick L.E., Ozbal C.C., Lamarr W.A. (2011). Advances in label-free screening approaches for studying histone acetyltransferases. J. Biomol. Screen..

[74] Camarillo J.M., Swaminathan S., Abshiru N.A., Sikora J.W., Thomas P.M., Kelleher N.L. (2019). Coupling fluorescence-activated cell sorting and targeted analysis of histone modification profiles in primary human leukocytes. J. Am. Soc. Mass Spectrom..

[75] Jing Y., Montano J.L., Levy M., Lopez J.E., Kung P.P., Richardson P. (2021). Harnessing ionic selectivity in acetyltransferase chemoproteomic probes. ACS Chem. Biol..

[76] Killenberg P.G., Dukes D.F. (1976). Coenzyme A derivatives of bile acids-chemical synthesis, purification, and utilization in enzymic preparation of taurine conjugates. J. Lipid Res..

[77] Emsley P., Cowtan K. (2004). model-building tools for molecular graphics. Acta Crystallogr. D.

[78] Tickle I.J., Flensburg C., Keller P., Paciorek W., Sharff A., Vonrhein C. (2018).

[79] Bricogne G., Blanc E., Brandl M., Flensburg C., Keller P., Paciorek W. (2011).

[80] Emsley P., Lohkamp B., Scott W.G., Cowtan K. (2010). Features and development of Coot. Acta Crystallogr. Sect. D Biol. Crystallogr..

[81] Thomas C.E., Kelleher N.L., Mizzen C.A. (2006). Mass spectrometric characterization of human histone H3: a bird's eye view. J. Proteome Res..

[82] Zheng S.P., Bi Y.C., Chen H.N., Gong B., Jia S.J., Li H.T. (2021). Molecular basis for bipartite recognition of histone H3 by the PZP domain of PHF14. Nucleic Acids Res..

[83] Jacquet K., Fradet-Turcotte A., Avvakumov N., Lambert J.P., Roques C., Pandita R.K. (2016). The TIP60 complex regulates bivalent chromatin recognition by 53BP1 through direct H4K20me binding and H2AK15 acetylation. Mol. Cell.

[84] Janas J.A., Zhang L.C., Luu J.H., Demeter J., Meng L.J., Marro S.G., Mall M. (2022). Tip60-mediated H2A.Z acetylation promotes neuronal fate specification and bivalent gene activation. Mol. Cell.

[85] Doyon Y., Selleck W., Lane W.S., Tan S., Cöté J. (2004). Structural and functional conservation of the NuA4 histone acetyltransferase complex from yeast to humans. Mol. Cell Biol..

[86] Yang Z.L., Mameri A., Cattoglio C., Lachance C., Ariza A.J.F., Luo J. (2024). Structural insights into the human NuA4/TIP60 acetyltransferase and chromatin remodeling complex. Science.

[87] Li C., Smirnova E., Schnitzler C., Crucifix C., Concordet J.P., Brion A. (2024). Structure of human TIP60-C histone exchange and acetyltransferase complex. Nature.

[88] Gaurav N., Kanai A., Lachance C., Cox K.L., Liu J.Y., Grzybowski A.T. (2024). Guiding the HBO1 complex function through the JADE subunit. Nat. Struct. Mol. Biol..

[89] Cai Y., Jin J., Swanson S.K., Cole M.D., Choi S.H., Florens L. (2010). Subunit composition and substrate specificity of a MOF-containing histone acetyltransferase distinct from the male-specific lethal (MSL) complex. J. Biol. Chem..

[90] Weber L.M., Jia Y.L., Stielow B., Gisselbrecht S.S., Cao Y.H., Ren Y.P. (2023). The histone acetyltransferase KAT6A is recruited to unmethylated CpG islands via a DNA binding winged helix domain. Nucleic Acids Res..

[91] Becht D.C., Klein B.J., Kanai A., Jang S.M., Cox K.L., Zhou B.R. (2023). MORF and MOZ acetyltransferases target unmethylated CpG islands through the winged helix domain. Nat. Commun..

[92] Klein B.J., Simithy J., Wang X., Ahn J., Andrews F.H., Zhang Y. (2017). Recognition of Histone H3K14 Acylation by MORF. Structure.

[93] Champagne N., Bertos N.R., Pelletier N., Wang A.H., Vezmar M., Yang Y. (1999). Identification of a human histone acetyltransferase related to monocytic leukemia zinc finger protein. J. Biol. Chem..

[94] Champagne N., Pelletier N., Yang X.J. (2001). The monocytic leukemia zinc finger protein MOZ is a histone acetyltransferase. Oncogene.

[95] Pelletier N., Champagne N., Stifani S., Yang X.J. (2002). MOZ and MORF histone acetyltransferases interact with the Runt-domain transcription factor Runx2. Oncogene.

[96] Champagne K.S., Saksouk N., Peña P.V., Johnson K., Ullah M., Yang X.J. (2008). The crystal structure of the ING5 PHD finger in complex with an H3K4me3 histone peptide. Proteins.

[97] Weiss M.S. (2001). Global indicators of X-ray data quality. J. Appl. Crystallogr..

