# Supplementary material for: Modulation of the substrate preference of a MYST acetyltransferase by a scaffold protein
Source: J Biol Chem. 2025 Feb 3;301(3):108262. doi: 10.1016/j.jbc.2025.108262 (PMC11946513; doi:10.1016/j.jbc.2025.108262)
Supplement: Supporting information [file mmc1.docx]

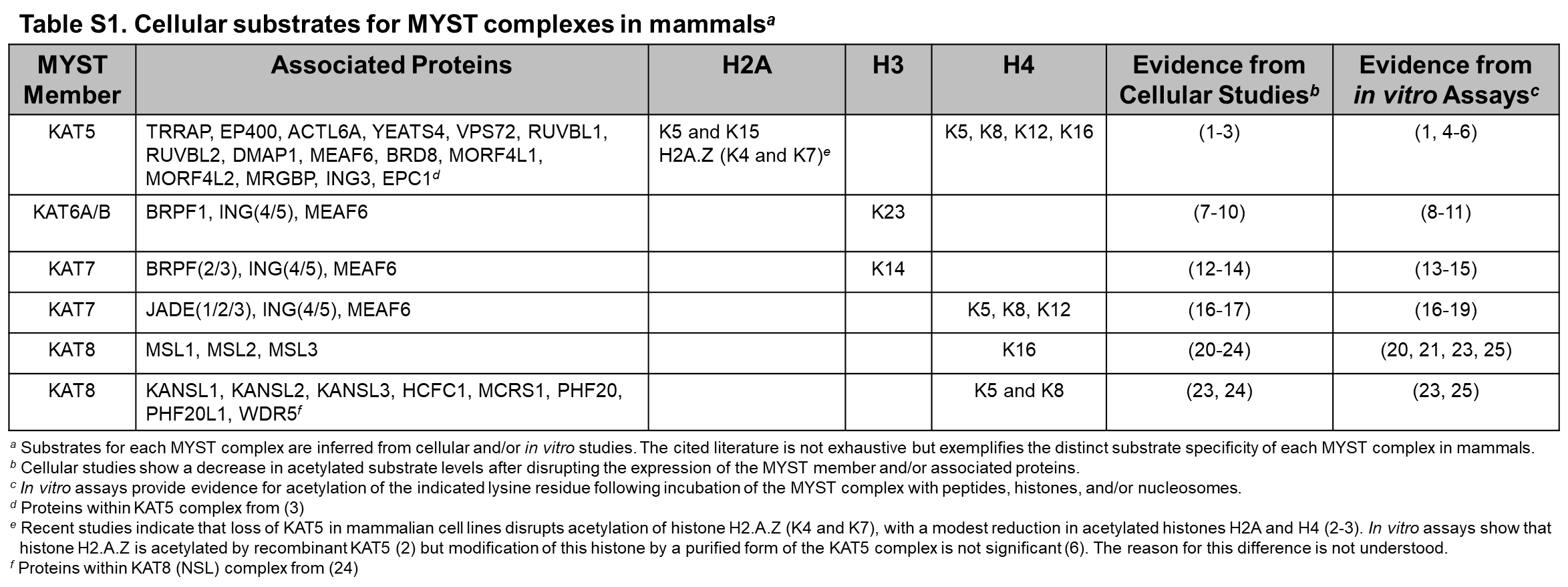


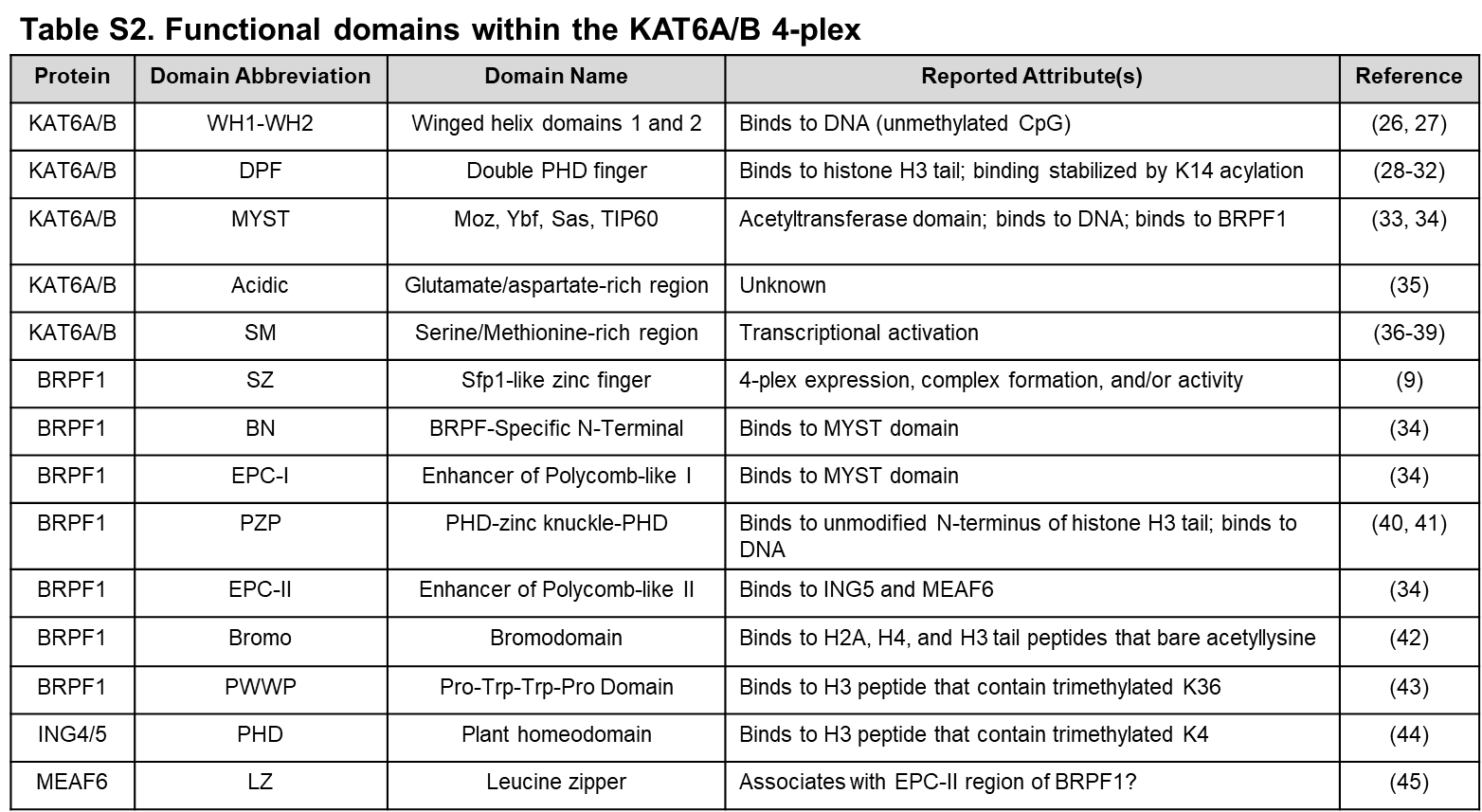


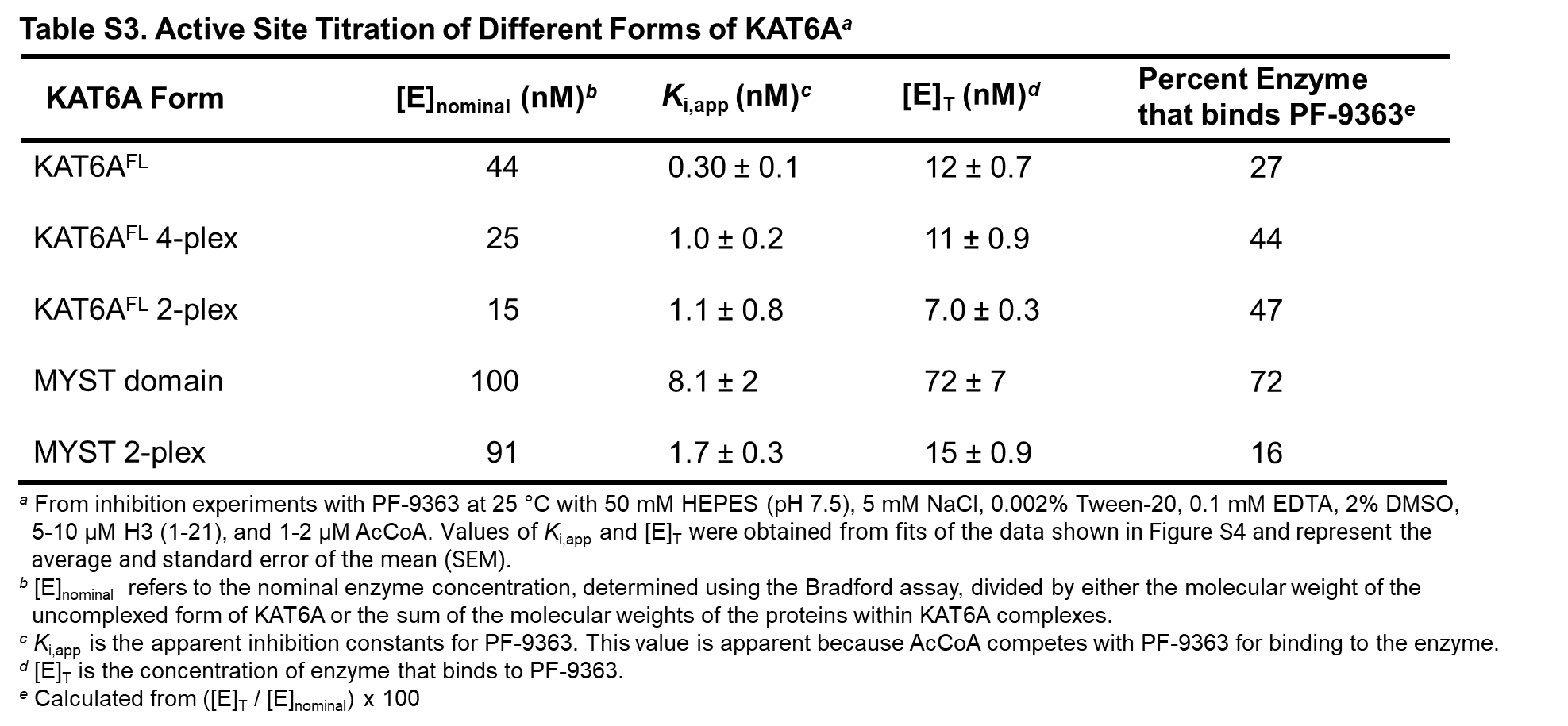


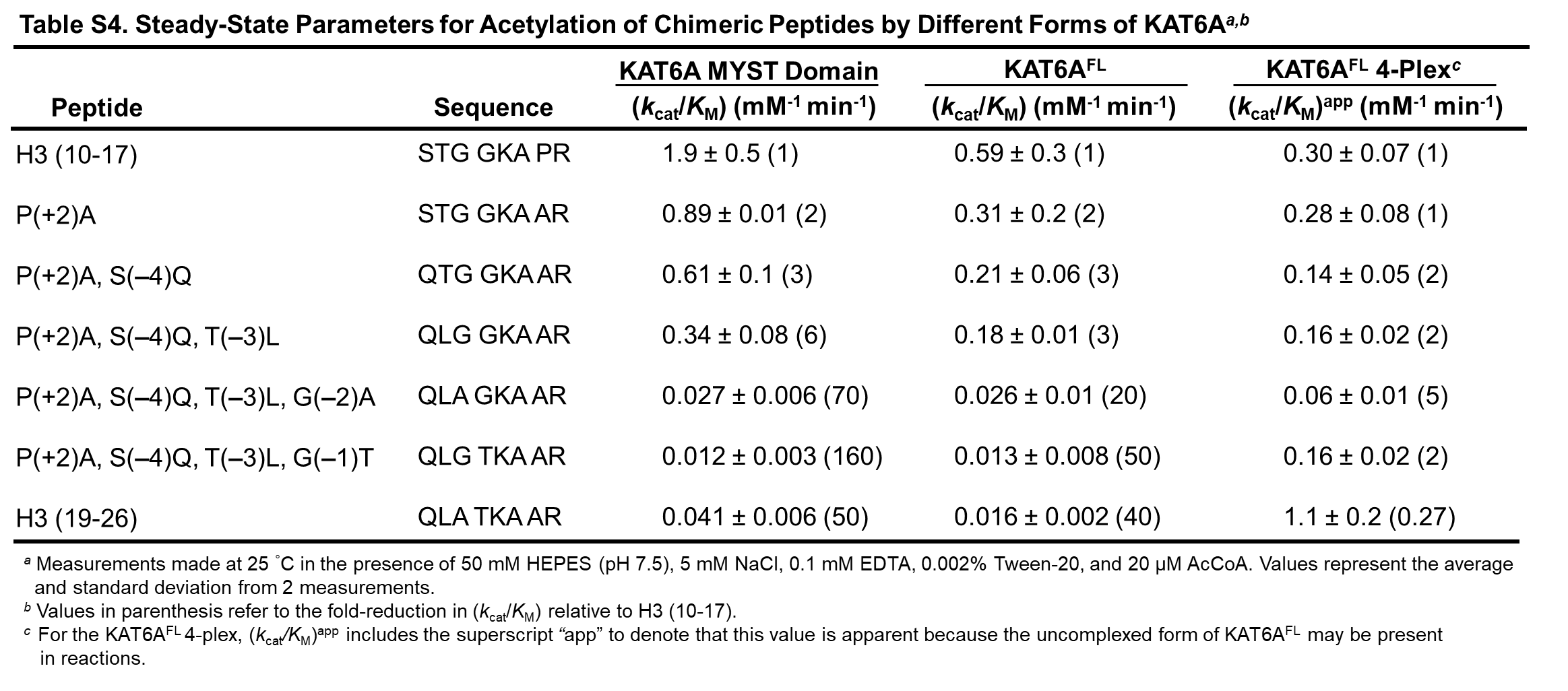


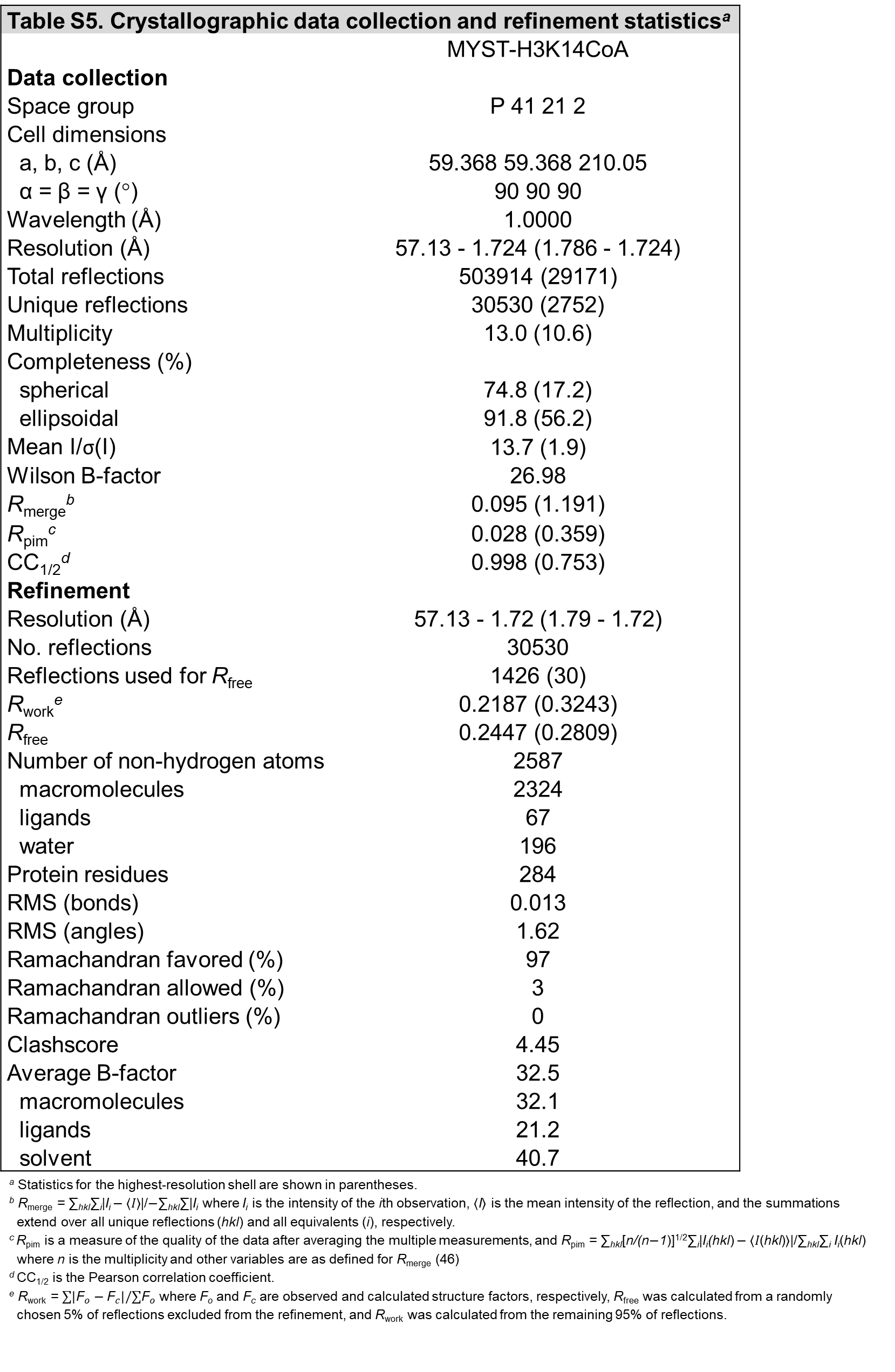


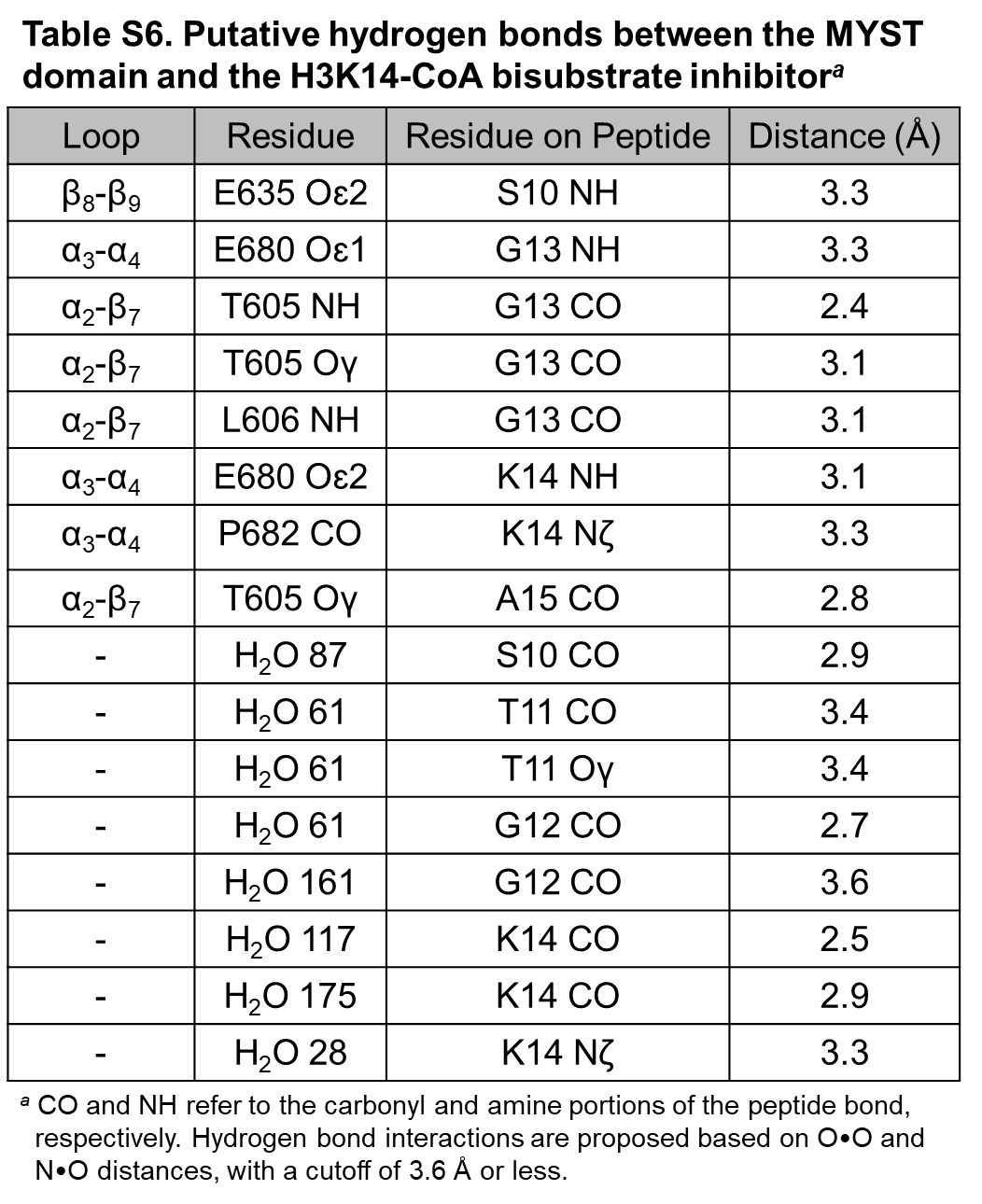


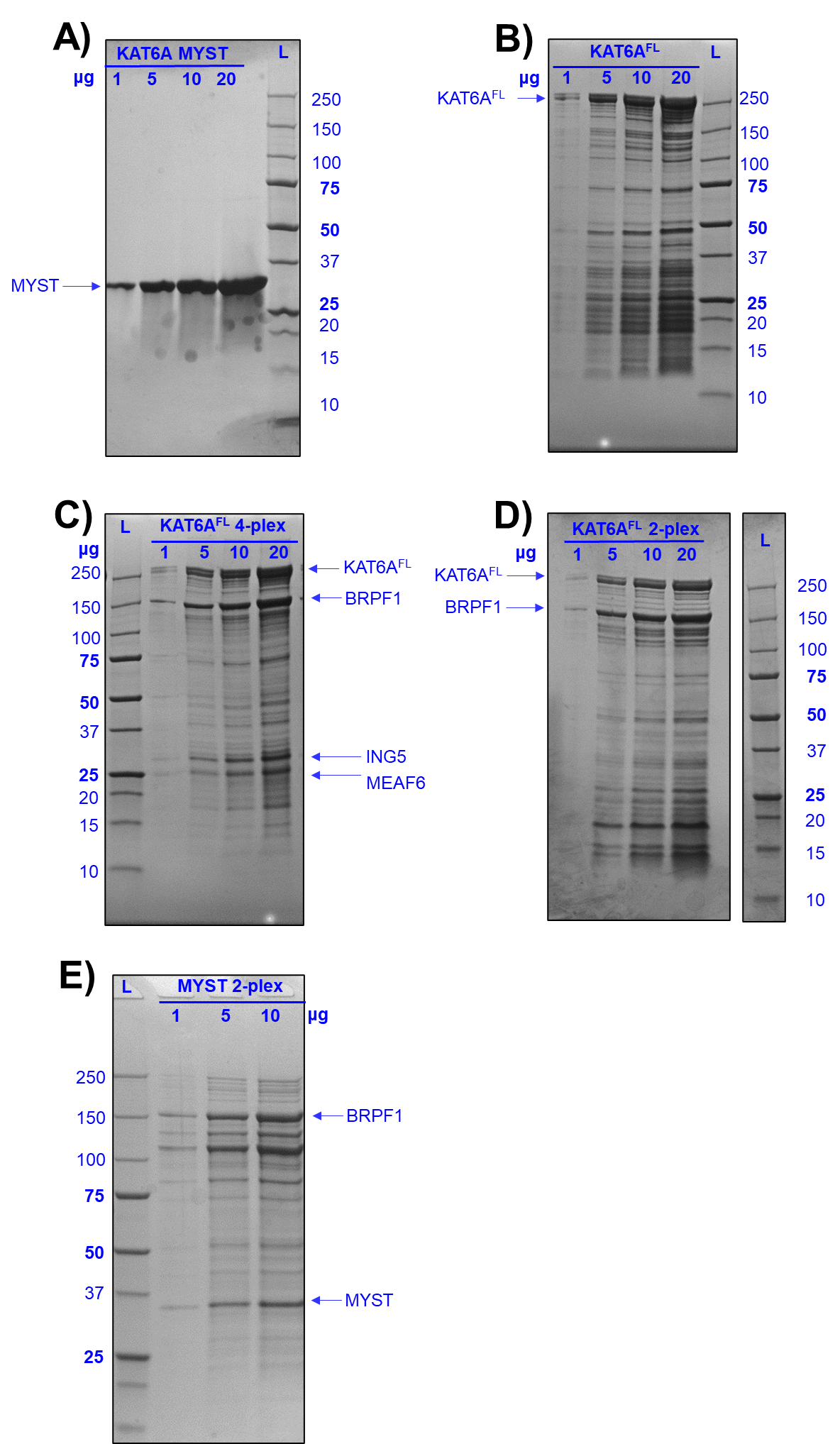


**Figure S1.** Analytical reducing SDS-PAGE gel of the KAT6A MYST domain (A), KAT6A^FL^ (B), the KAT6A^FL^ 4-plex (C), the KAT6A^FL^ 2-plex (D), and the MYST 2-plex (E). For each form of KAT6A, the SDS-PAGE gel includes a molecular weight protein ladder (L) as well different amounts of each form of KAT6A following nickel affinity capture (or FLAG-tag purification),
gel filtration chromatography, and protein concentration. In panel D, intervening lanes between the protein sample and the ladder were removed for clarity.


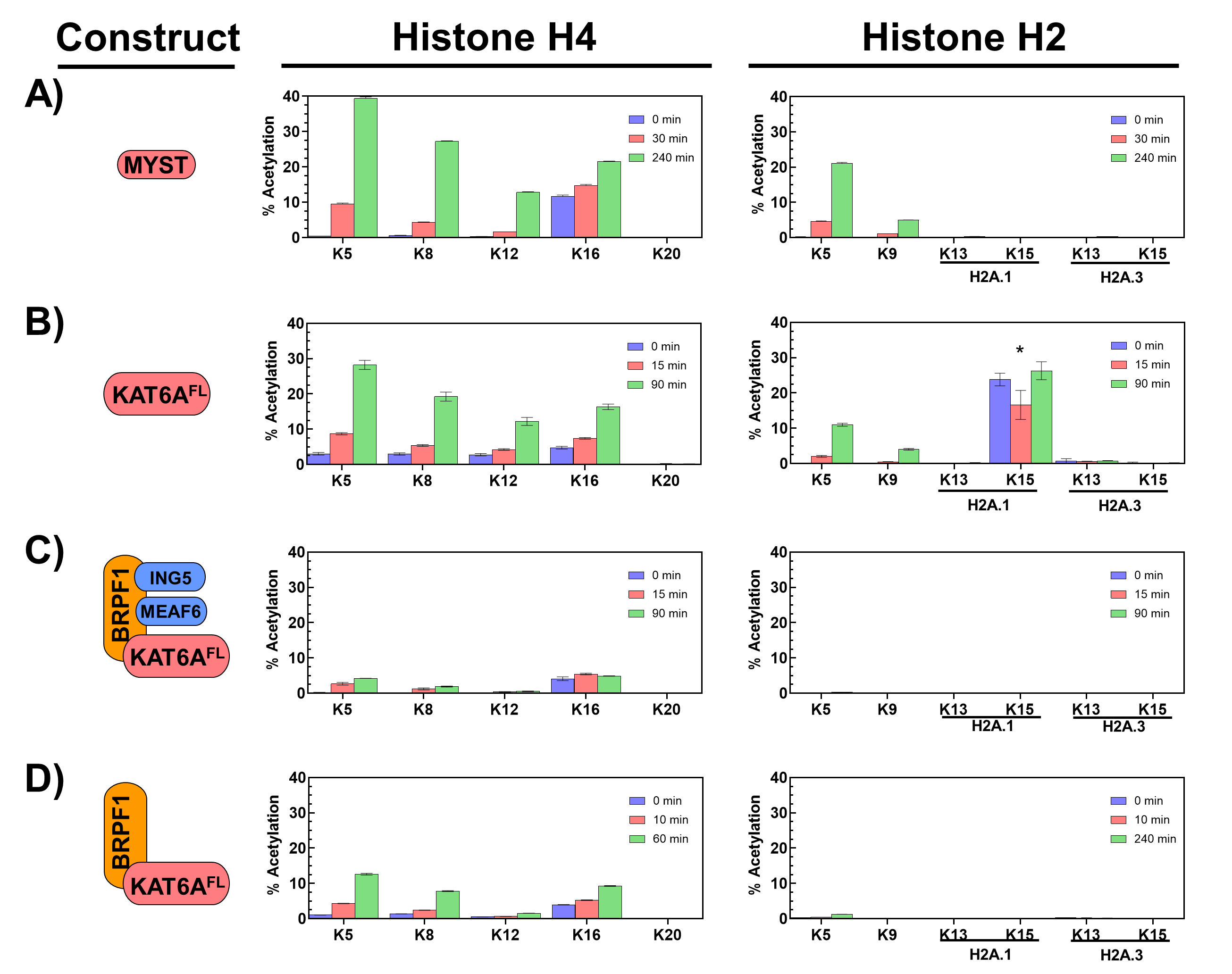


**Figure S2.** Acetylation of lysine residues within the N-terminal tails of histones H4 and H2A following incubation of HeLa oligonucleosomes with the KAT6A MYST domain (A), KAT6A^FL^ (B), the KAT6A^FL^ 4-plex (C), and the KAT6A^FL^ 2-plex (D). Reactions were allowed to proceed at various times before being quenched in formic acid for subsequent MS analysis, as described in Materials and Methods. Data for the KAT6A^FL^ 4-plex reproduced from (10). Values correspond to the average and standard deviation of 3 technical replicates. For modification of H2A.1K15 by KAT6A^FL^ (denoted by an asterisk), we observe elevated acetylation at this residue. However, the % acetylation at 0, 15, and 90 minutes is similar, indicating that enzyme-catalyzed acetylation at this residue is minimal.


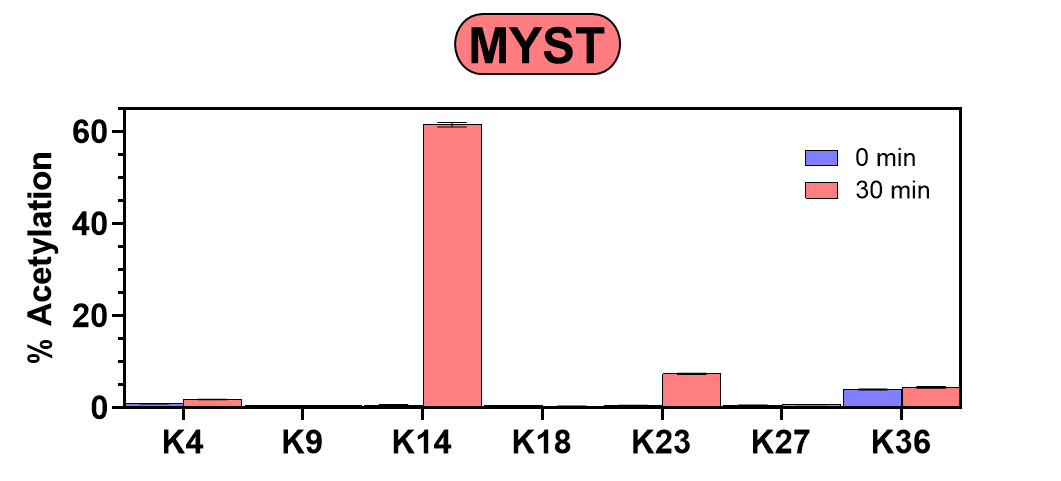


**Figure S3.** Acetylation of lysine residues within H3.3 (1-43) following incubation with the KAT6A MYST domain. The reaction was performed at room temperature with 50 mM HEPES (pH 7.5), 5 mM NaCl, 0.1 mM EDTA, 50 μM H3.3 (1-43), 50 μM acetyl-CoA, and 600 nM KAT6A MYST domain. Acetylation was followed for 30 minutes before being quenched in formic acid for subsequent MS analysis, as described in Materials and Methods. Values correspond to the average and standard deviation of 3 technical replicates.


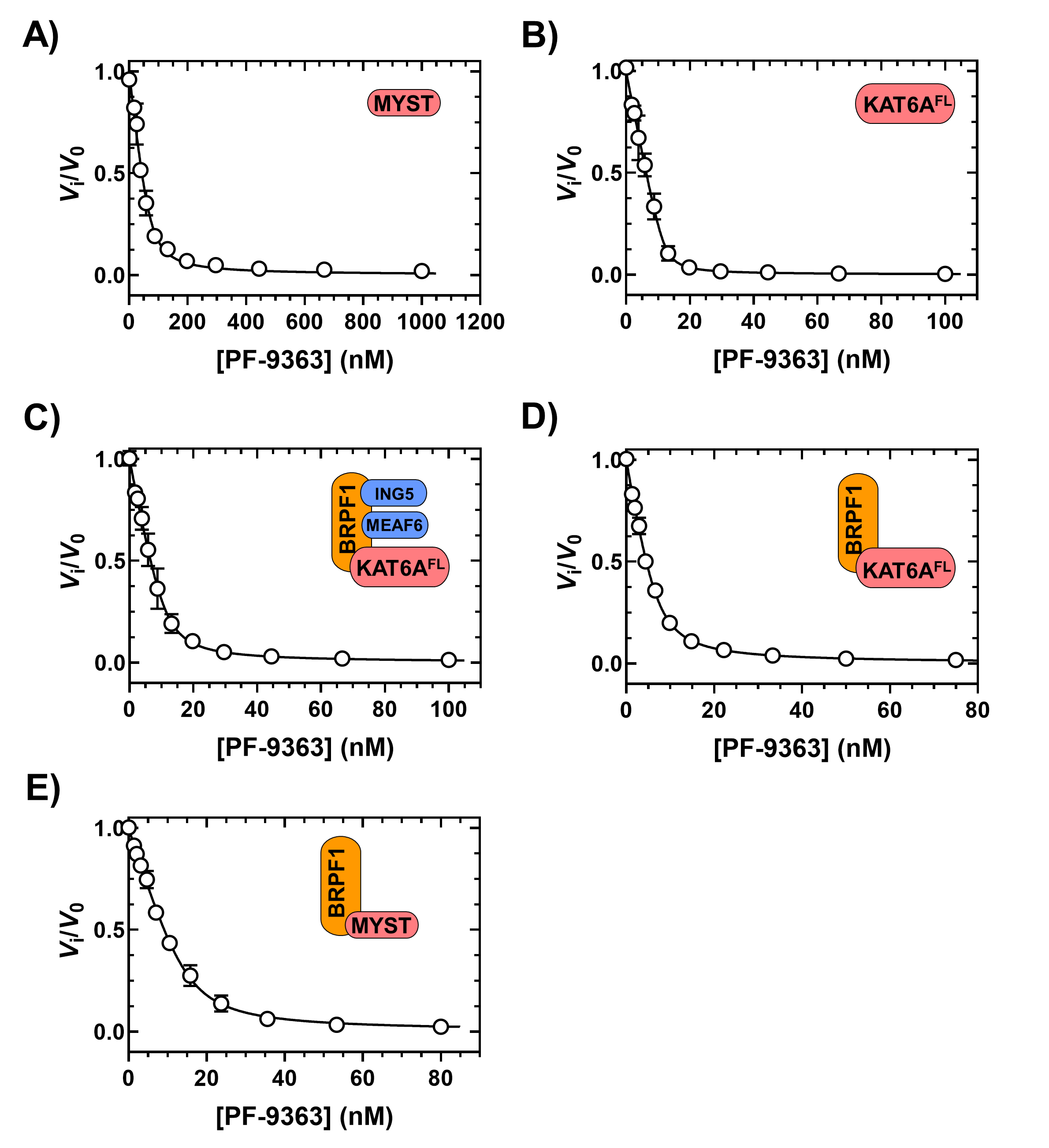


**Figure S4.** Determination of the active enzyme concentration of the KAT6A MYST domain (A), KAT6A^FL^ (B), the KAT6A^FL^ 4-plex (C), the KAT6A^FL^ 2-plex (D), and the MYST 2-plex (E). Active site titrations for each form of KAT6A were carried out through tight-binding inhibition by PF-9363. Reactions were performed with H3 (1-21) (5-10 µM) and acetyl-CoA (1-2 µM) with various concentrations of PF-9363 (0 – 1000 nM) in the presence of 50 mM HEPES (pH 7.5), 5 mM NaCl, 0.002% Tween-20, 0.1 mM EDTA, and 2% DMSO at 25 ^°^C. The initial velocity measured at a given concentration of PF-9363 (*V*_i_) was normalized with respect to the initial velocity in the absence of inhibitor (*V*_0_). Within each figure, each value represents the average and standard deviation from 2-3 measurements. The solid line was obtained from a fit of the data to equation 1 in Materials and Methods, with the apparent affinity and active enzyme concentration reported in Table S3.


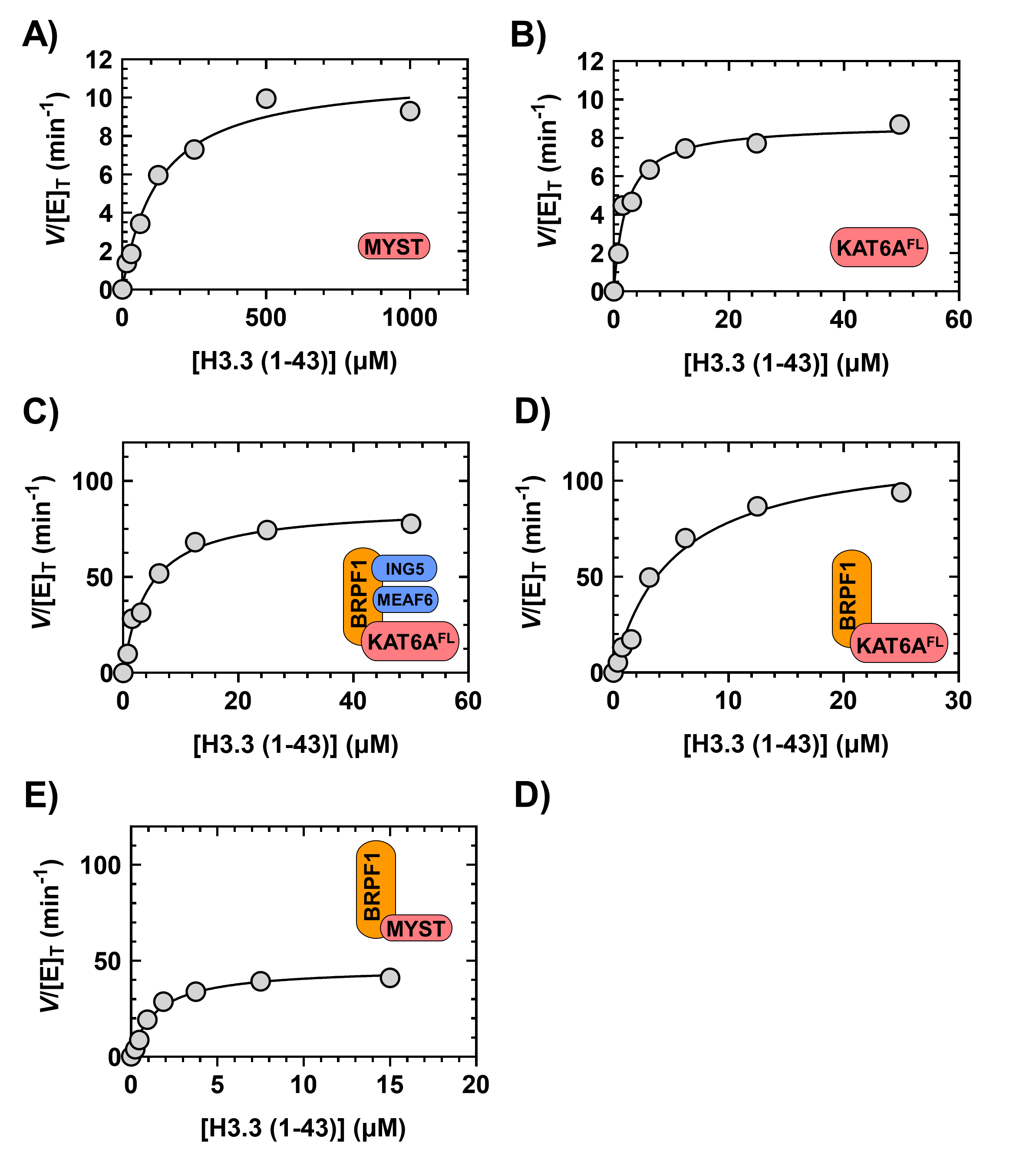


**Figure S5.** Acetylation of H3.3 (1-43) by the KAT6A MYST domain (A), KAT6A^FL^ (B), the KAT6A^FL^ 4-plex (C), the KAT6A^FL^ 2-plex (D), and the MYST 2-plex (E). Reactions were performed at 25 ^°^C in the presence of 50 mM HEPES (pH 7.5), 5 mM NaCl, 0.002% Tween-20, 0.1 mM EDTA, and 20 μM acetyl-CoA. The lines are fits of the data from equation 2 in Materials and Methods. Experiments were performed in duplicate with representative plots shown.


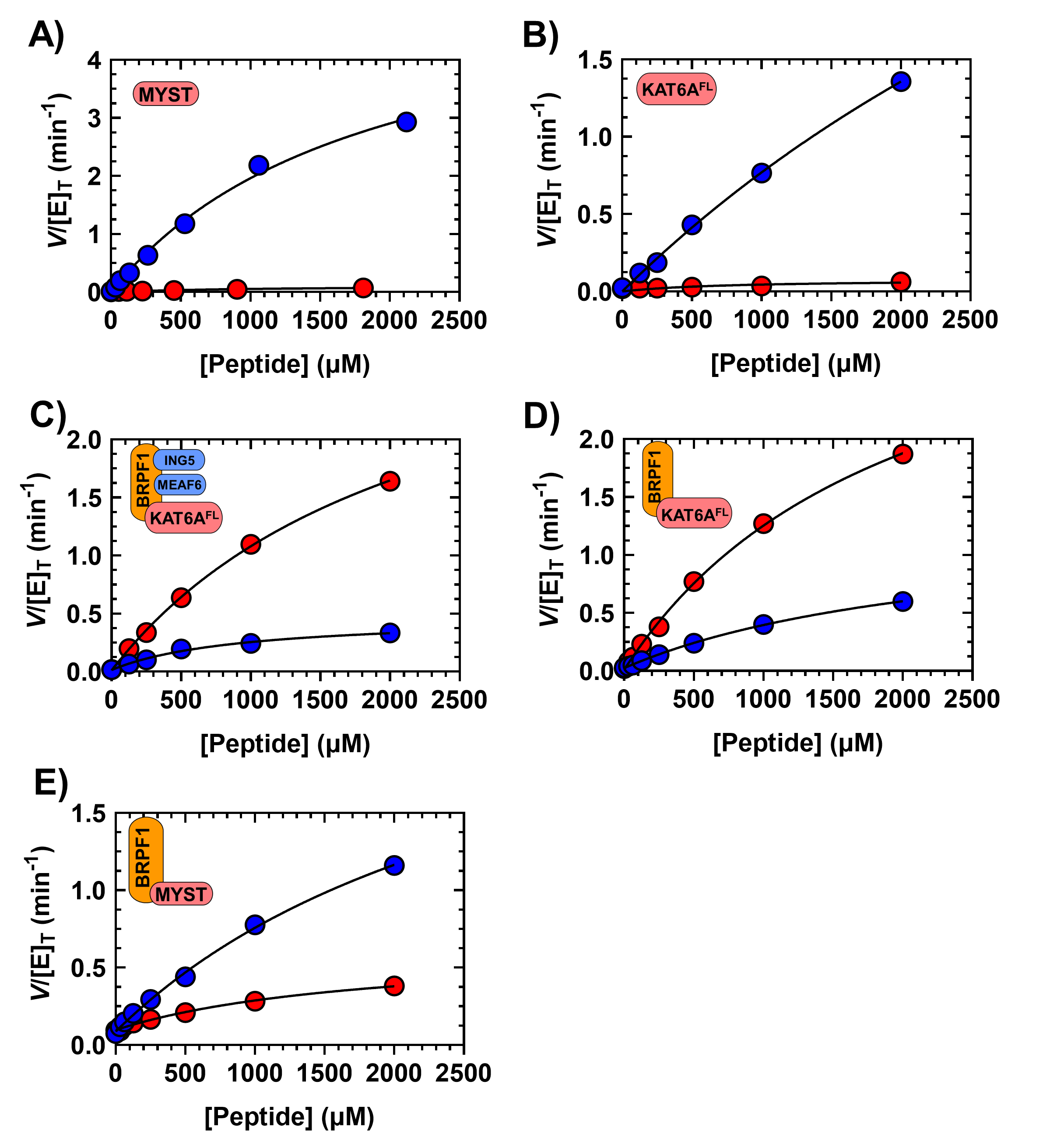


**Figure S6.** Acetylation of H3 (10-17) (⚫) and H3 (19-26) (⚫) by the KAT6A MYST domain (A), KAT6A^FL^ (B), the KAT6A^FL^ 4-plex (C), the KAT6A^FL^ 2-plex (D), and the MYST 2-plex (E). Reactions were performed at 25 ^°^C in the presence of 50 mM HEPES (pH 7.5), 5 mM NaCl, 0.002% Tween-20, 0.1 mM EDTA, and 20 μM acetyl-CoA. The lines are fits of the data from equation 2 in Materials and Methods. Experiments were performed in duplicate with representative plots shown.


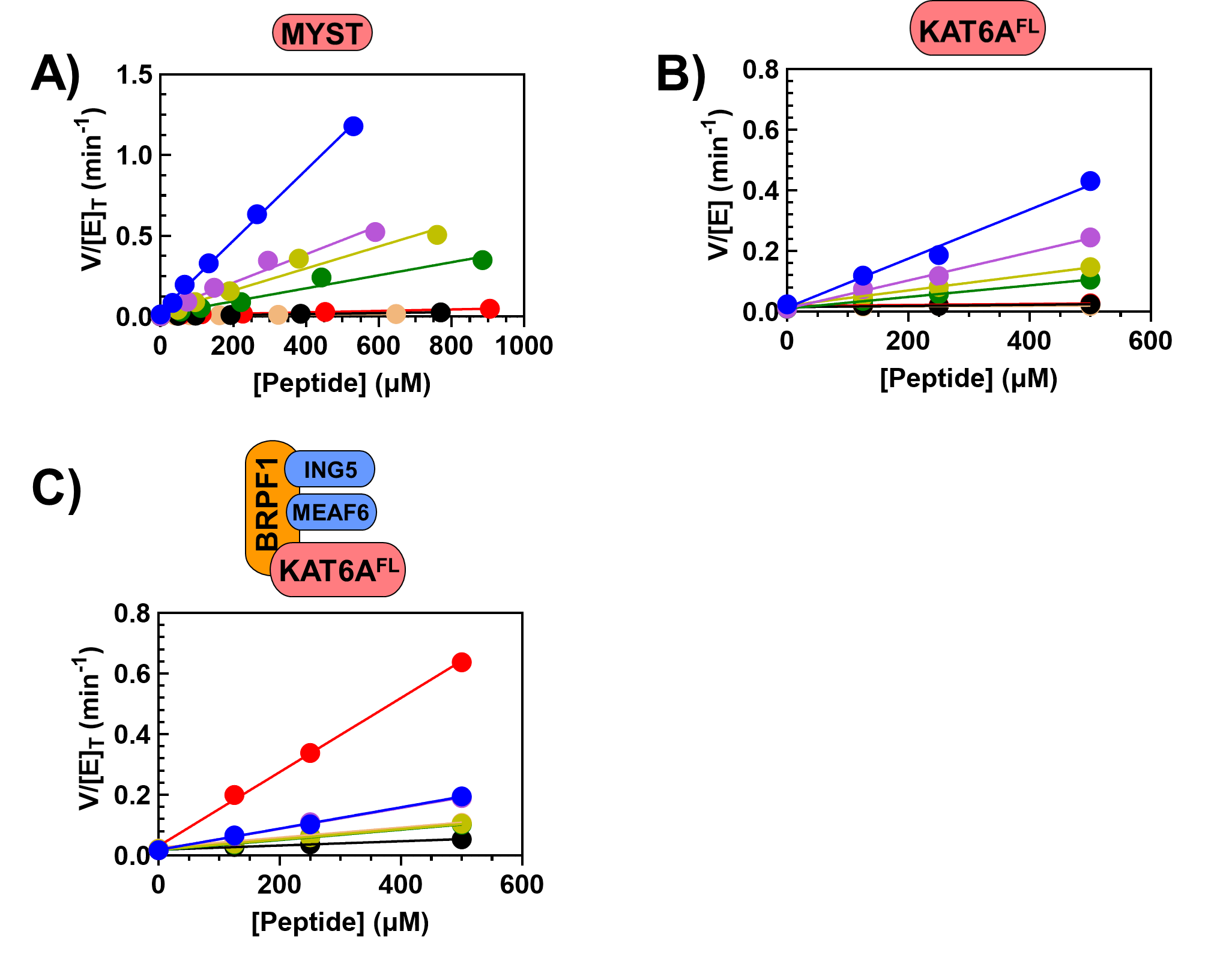


**Figure S7.** Acetylation of H3 chimeric peptides by the KAT6A MYST domain (A), KAT6A^FL^ (B), and the KAT6A^FL^ 4-plex (C), Peptides assayed include H3 (10-17) (⚫), P(+2)A (⚫), P(+2)A, S(–4)Q (⚫), P(+2)A, S(–4)Q, T(–3)L (⚫), P(+2)A, S(–4)Q, T(–3)L, G(–2)A (⚫), P(+2)A, S(–4)Q, T(–3)L, G(–1)T (⚫), and H3 (19-26) (⚫). Reactions were performed at 25 ^°^C in the presence of 50 mM HEPES (pH 7.5), 5 mM NaCl, 0.002% Tween-20, 0.1 mM EDTA, 20 μM acetyl-CoA, and at [peptide] ≤1,000 µM. The data are fit to a linear equation. Experiments were performed in duplicate with representative plots shown.


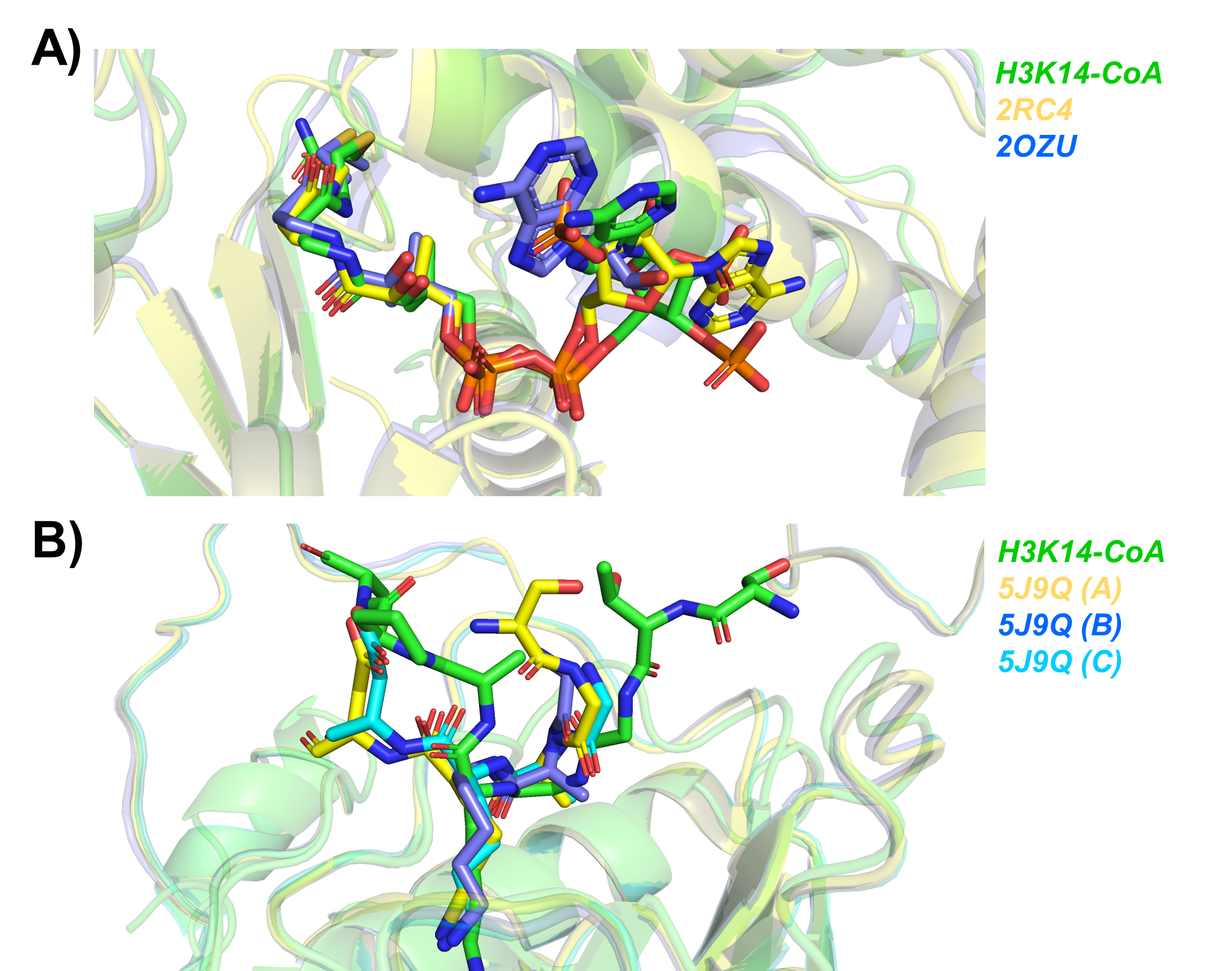


**Figure S8.** Overlay of the H3K14-CoA bisubstrate inhibitor (H3K14-CoA, green) with prior crystallographic data at the AcCoA binding site (A) and the peptide binding site (B). 2RC4 (yellow) and 2OZU (blue) refer to PDB accession numbers for two structures of the KAT6A MYST domain complexed with acetyl-CoA. 5J9Q refers to the PDB accession number for the NuA4 core complex, with A (yellow), B (blue), and C (cyan) corresponding to the 3 molecules of the asymmetric unit.


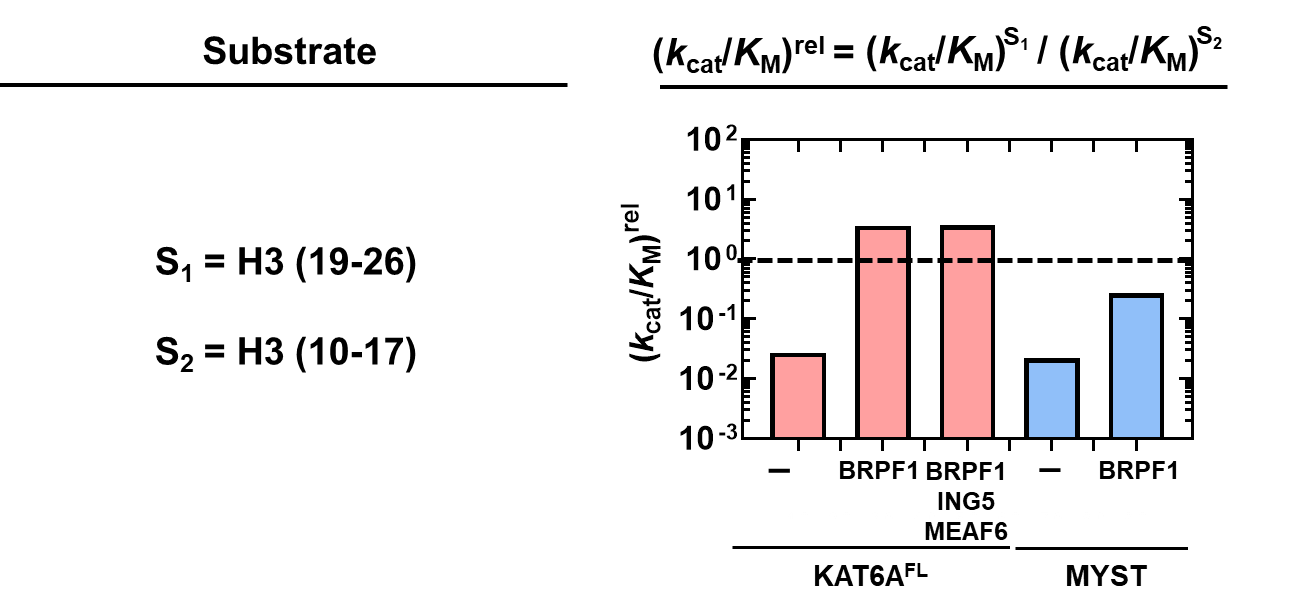


**Figure S9.** Changes in *k*_cat_/*K*_M_ for H3 (19-26) relative to H3 (10-17) for different forms of KAT6A. (*k*_cat_/*K*_M_)^rel^ refers to *k*_cat_/*K*_M_ or (*k*_cat_/*K*_M_)^app^ for H3 (19-26) relative to that for H3 (10-17) for each form of KAT6A. The dashed line corresponds to (*k*_cat_/*K*_M_)^rel^ = 1.


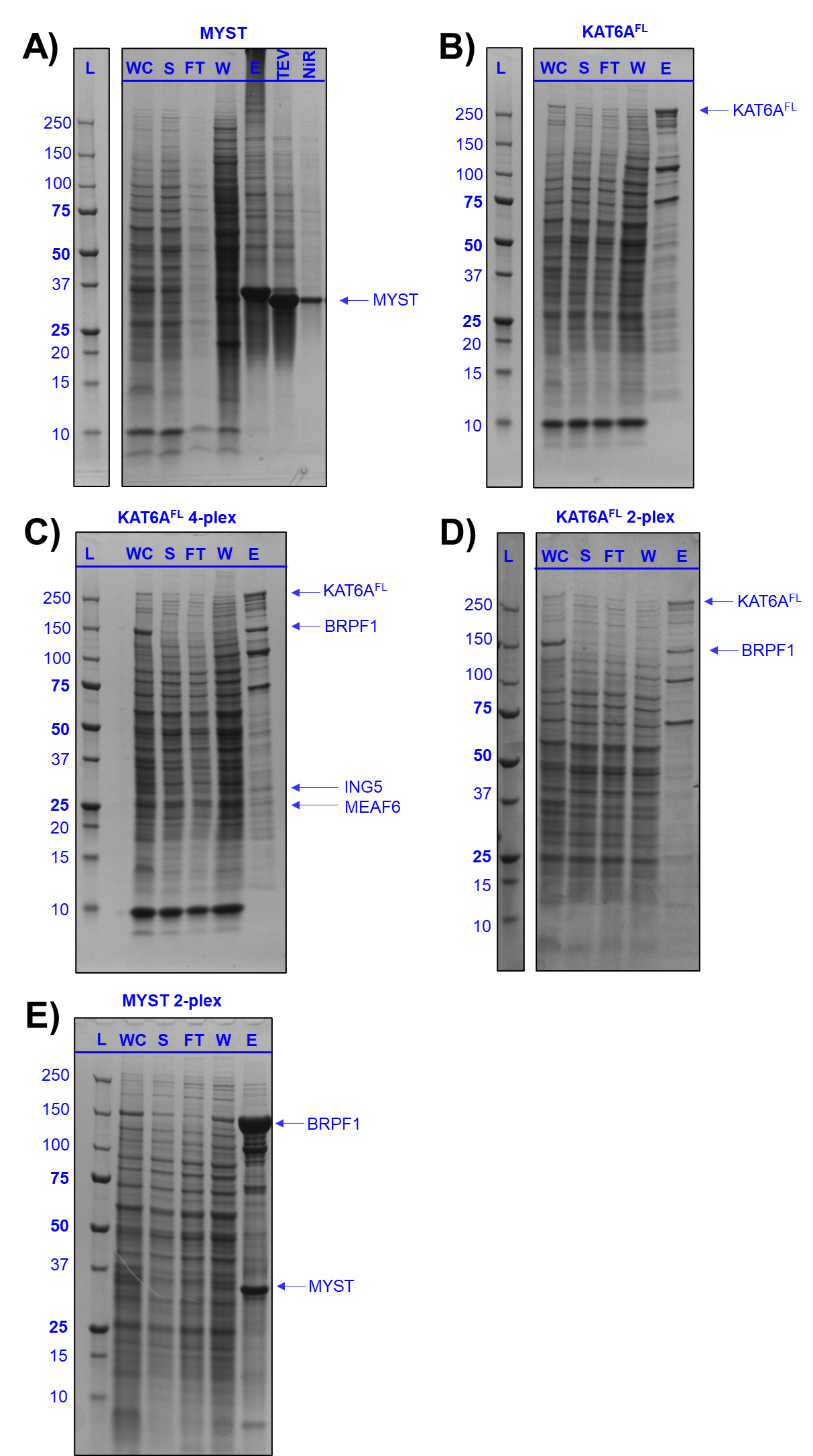


**Figure S10. Purification of the KAT6A MYST domain (A), KAT6A^FL^ (B), the KAT6A^FL^ 4-plex (C), the KAT6A^FL^ 2-plex (D) and the MYST 2-plex (E).** For each form of KAT6A, the SDS-PAGE gel includes a molecular weight protein ladder (L) as well as whole-cell lysate (WC) and soluble extract (S). The KAT6A MYST domain was purified via nickel affinity capture, with the flowthrough (FT), wash (W) and elute (E) fractions shown. The protein was subsequently treated with TEV protease (TEV) to cleave the N-terminal polyhistidine tag and purified via reverse nickel affinity chromatography (NiR). All other forms of KAT6A were purified by FLAG-tag purification, with the flowthrough (FT), wash (W) and elute (E) fractions shown. In panels A, B, and D, intervening lanes between the protein sample and the ladder were removed for clarity.


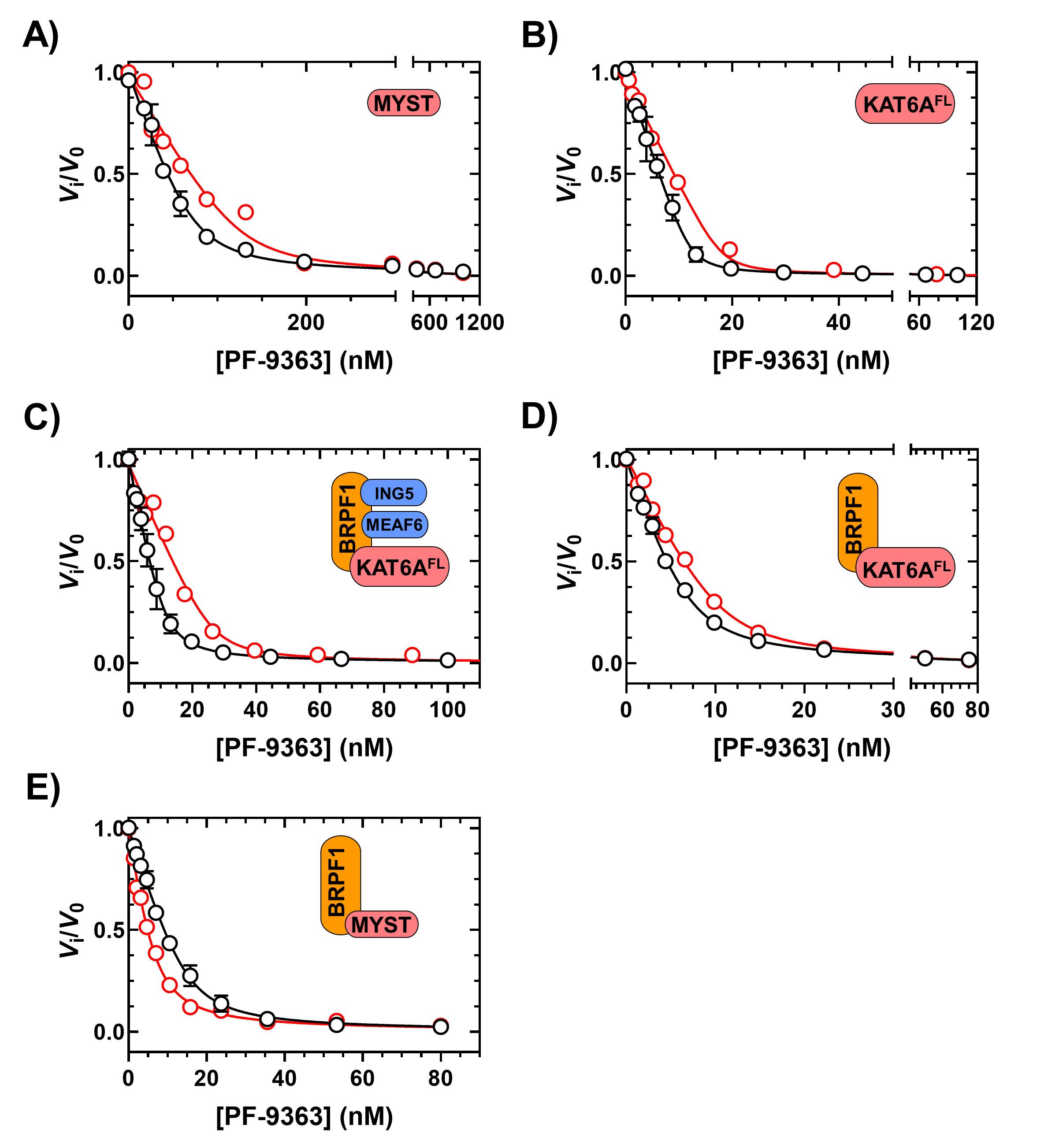


**Figure S11. Tight-binding inhibition by PF-9363 at different nominal enzyme concentrations (E_nominal_).** For the KAT6A MYST domain (A), [E]_nominal_ is 100 (🌕) and (🌕) 200 nM. For KAT6A^FL^ (B), [E]_nominal_ is 44 (🌕) and 68 (🌕) nM. For the KAT6A^FL^ 4-plex (C), [E]_nominal_ is 25 (🌕) and 50 (🌕) nM. For the KAT6A^FL^ 2-plex (D), [E]_nominal_ is 15 (🌕) and 25 (🌕) nM. For the MYST 2-plex (E), [E]_nominal_ is 91 (🌕) and 60 (🌕) nM. The initial velocity measured at a given concentration of PF-9363 (*V*_i_) was normalized with respect to the initial velocity in the absence of inhibitor (*V*_0_). Values in black are reproduced from the data shown in Figure S4 and the black lines were obtained from fits of the data to a quadratic binding isotherm, with the apparent affinity of PF-9363 and active enzyme concentration summarized in Table S3. The red lines correspond to quadratic fits of the data at a different [E]_nominal_, with the apparent affinity of PF-9363 constrained to the value reported in Table S3 for the given KAT6A form.

**References**

1. Jacquet, K., Fradet-Turcotte, A., Avvakumov, N., Lambert, J. P., Roques, C., Pandita, R. K., Paquet, E., Herst, P., Gingras, A. C., Pandita, T. K., Legube, G., Doyon, Y., Durocher, D., and Côté, J. (2016) The TIP60 Complex Regulates Bivalent Chromatin Recognition by 53BP1 through Direct H4K20me Binding and H2AK15 Acetylation. *Molecular Cell* **62**, 409-421

2. Wichmann, J., Pitt, C., Eccles, S., Garnham, A. L., Li-Wai-Suen, C. S. N., May, R., Allan, E., Wilcox, S., Herold, M. J., Smyth, G. K., Monahan, B. J., Thomas, T., and Voss, A. K. (2022) Loss of TIP60 (KAT5) abolishes H2AZ lysine 7 acetylation and causes p53, INK4A, and ARF-independent cell cycle arrest. *Cell Death Dis* **13**, 627

3. Janas, J. A., Zhang, L. C., Luu, J. H., Demeter, J., Meng, L. J., Marro, S. G., Mall, M., Mooney, N. A., Schaukowitch, K., Ng, Y. H., Yang, N., Huang, Y. H., Neumayer, G., Gozani, O., Elias, J. E., Jackson, P. K., and Wernig, M. (2022) Tip60-mediated H2A.Z acetylation promotes neuronal fate specification and bivalent gene activation. *Molecular Cell* **82**, 4627-+

4. Doyon, Y., Selleck, W., Lane, W. S., Tan, S., and Cöté, J. (2004) Structural and functional conservation of the NuA4 histone acetyltransferase complex from yeast to humans. *Molecular and Cellular Biology* **24**, 1884-1896

5. Yang, Z. L., Mameri, A., Cattoglio, C., Lachance, C., Ariza, A. J. F., Luo, J., Humbert, J., Sudarshan, D., Banerjea, A., Galloy, M., Fradet-Turcotte, A., Lambert, J. P., Ranish, J. A., Côté, J., and Nogales, E. (2024) Structural insights into the human NuA4/TIP60 acetyltransferase and chromatin remodeling complex. *Science* **385**

6. Li, C., Smirnova, E., Schnitzler, C., Crucifix, C., Concordet, J. P., Brion, A., Poterszman, A., Schultz, P., Papai, G., and Ben-Shem, A. (2024) Structure of human TIP60-C histone exchange and acetyltransferase complex. *Nature*

7. You, L. Y., Li, L., Zou, J. F., Yan, K. Z., Belle, J., Nijnik, A., Wang, E., and Yang, X. J. (2016) BRPF1 is essential for development of fetal hematopoietic stem cells. *J Clin Invest* **126**, 3247-3262

8. Yan, K. Z., Rousseau, J., Littlejohn, R. O., Kiss, C., Lehman, A., Rosenfeld, J. A., Stumpel, C. T. R., Stegmann, A. P. A., Robak, L., Scaglia, F., Nguyen, T. T. M., Fu, H., Ajeawung, N. F., Camurri, M. V., Li, L., Gardham, A., Panis, B., Almannai, M., Sacoto, M. J. G., Baskin, B., Ruivenkamp, C., Xia, F., Bi, W., Cho, M. T., Potjer, T. P., Santen, G. W. E., Parker, M. J., Canham, N., McKinnon, M., Potocki, L., MacKenzie, J. J., Roeder, E. R., Campeau, P. M., Yang, X. J., Study, D., and Study, C. (2017) Mutations in the Chromatin Regulator Gene *BRPF1* Cause Syndromic Intellectual Disability and Deficient Histone Acetylation. *American Journal of Human Genetics* **100**, 91-104

9. Yan, K., Rousseau, J., Machol, K., Cross, L. A., Agre, K. E., Gibson, C. F., Goverde, A., Engleman, K. L., Verdin, H., De Baere, E., Potocki, L., Zhou, D., Cadieux-Dion, M., Bellus, G. A., Wagner, M. D., Hale, R. J., Esber, N., Riley, A. F., Solomon, B. D., Cho, M. T., McWalter, K., Eyal, R., Hainlen, M. K., Mendelsohn, B. A., Porter, H. M., Lanpher, B. C., Lewis, A. M., Savatt, J., Thiffault, I., Callewaert, B., Campeau, P. M., and Yang, X. J. (2020) Deficient histone H3 propionylation by BRPF1-KAT6 complexes in neurodevelopmental disorders and cancer. *Sci Adv* **6**, eaax0021

10. Sharma, S., Chung, C. Y., Uryu, S., Petrovic, J., Cao, J., Rickard, A., Nady, N., Greasley, S., Johnson, E., Brodsky, O., Khan, S., Wang, H., Wang, Z., Zhang, Y., Tsaparikos, K., Chen, L., Mazurek, A., Lapek, J., Kung, P. P., Sutton, S., Richardson, P. F., Greenwald, E. C., Yamazaki, S., Jones, R., Maegley, K. A., Bingham, P., Lam, H., Stupple, A. E., Kamal, A., Chueh, A., Cuzzupe, A., Morrow, B. J., Ren, B., Carrasco-Pozo, C., Tan, C. W., Bhuva, D. D., Allan, E., Surgenor, E., Vaillant, F., Pehlivanoglu, H., Falk, H., Whittle, J. R., Newman, J., Cursons, J., Doherty, J. P., White, K. L., MacPherson, L., Devlin, M., Dennis, M. L., Hattarki, M. K., De Silva, M., Camerino, M. A., Butler, M. S., Dolezal, O., Pilling, P., Foitzik, R., Stupple, P. A., Lagiakos, H. R., Walker, S. R., Hediyeh-Zadeh, S., Nuttall, S., Spall, S. K., Charman, S. A., Connor, T., Peat, T. S., Avery, V. M., Bozikis, Y. E., Yang, Y., Zhang, M., Monahan, B. J., Voss, A. K., Thomas, T., Street, I. P., Dawson, S. J., Dawson, M. A., Lindeman, G. J., Davis, M. J., Visvader, J. E., and Paul, T. A. (2023) Discovery of a highly potent, selective, orally bioavailable inhibitor of KAT6A/B histone acetyltransferases with efficacy against KAT6A-high ER+ breast cancer. *Cell Chem Biol* **30**, 1191-1210 e1120

11. Klein, B. J., Jang, S. M., Lachance, C., Mi, W., Lyu, J., Sakuraba, S., Krajewski, K., Wang, W. W., Sidoli, S., Liu, J., Zhang, Y., Wang, X., Warfield, B. M., Kueh, A. J., Voss, A. K., Thomas, T., Garcia, B. A., Liu, W. R., Strahl, B. D., Kono, H., Li, W., Shi, X., Côté, J., and Kutateladze, T. G. (2019) Histone H3K23-specific acetylation by MORF is coupled to H3K14 acylation. *Nat Commun* **10**, 4724

12. Kueh, A. J., Dixon, M. P., Voss, A. K., and Thomas, T. (2011) HBO1 Is Required for H3K14 Acetylation and Normal Transcriptional Activity during Embryonic Development. *Molecular and Cellular Biology* **31**, 845-860

13. Mishima, Y., Miyagi, S., Saraya, A., Negishi, M., Endoh, M., Endo, T. A., Toyoda, T., Shinga, J., Katsumoto, T., Chiba, T., Yamaguchi, N., Kitabayashi, I., Koseki, H., and Iwama, A. (2011) The Hbo1-Brd1/Brpf2 complex is responsible for global acetylation of H3K14 and required for fetal liver erythropoiesis. *Blood* **118**, 2443-2453

14. Feng, Y. P., Vlassis, A., Roques, C., Lalonde, M. E., González-Aguilera, C., Lambert, J. P., Lee, S. B., Zhao, X. B., Alabert, C., Johansen, J. V., Paquet, E., Yang, X. J., Gingras, A. C., Côté, J., and Groth, A. (2016) BRPF3-HBO1 regulates replication origin activation and histone H3K14 acetylation. *Embo Journal* **35**, 176-192

15. Yan, K. Z., You, L. Y., Degerny, C., Ghorbani, M., Liu, X., Chen, L. L., Li, L., Miao, D. S., and Yang, X. J. (2016) The Chromatin Regulator BRPF3 Preferentially Activates the HBO1 Acetyltransferase but Is Dispensable for Mouse Development and Survival. *Journal of Biological Chemistry* **291**, 2647-2663

16. Foy, R. L., Song, I. Y., Chitalia, V. C., Cohen, H. T., Saksouk, N., Cayrou, C., Vaziri, C., Côté, J., and Panchenko, M. V. (2008) Role of Jade-1 in the histone acetyltransferase (HAT) HBO1 complex. *J Biol Chem* **283**, 28817-28826

17. Miotto, B., and Struhl, K. (2010) HBO1 Histone Acetylase Activity Is Essential for DNA Replication Licensing and Inhibited by Geminin. *Molecular Cell* **37**, 57-66

18. Lalonde, M. E., Avvakumov, N., Glass, K. C., Joncas, F. H., Saksouk, N., Holliday, M., Paquet, E., Yan, K., Tong, Q., Klein, B. J., Tan, S., Yang, X. J., Kutateladze, T. G., and Côté, J. (2013) Exchange of associated factors directs a switch in HBO1 acetyltransferase histone tail specificity. *Genes Dev* **27**, 2009-2024

19. Gaurav, N., Kanai, A., Lachance, C., Cox, K. L., Liu, J. Y., Grzybowski, A. T., Saksouk, N., Klein, B. J., Komata, Y., Asada, S., Ruthenburg, A. J., Poirier, M. G., Coté, J., Yokoyama, A., and Kutateladze, T. G. (2024) Guiding the HBO1 complex function through the JADE subunit. *Nature Structural & Molecular Biology* **31**

20. Taipale, M., Rea, S., Richter, K., Vilar, A., Lichter, P., Imhof, A., and Akhtar, A. (2005) hMOF histone acetyltransferase is required for histone H4 lysine 16 acetylation in mammalian cells. *Molecular and Cellular Biology* **25**, 6798-6810

21. Smith, E. R., Cayrou, C., Huang, R., Lane, W. S., Côté, J., and Lucchesi, J. C. (2005) A human protein complex homologous to the MSL complex is responsible for the majority of histone H4 acetylation at lysine 16. *Molecular and Cellular Biology* **25**, 9175-9188

22. Thomas, T., Dixon, M. P., Kueh, A. J., and Voss, A. K. (2008) Mof (MYST1 or KAT8) is essential for progression of embryonic development past the blastocyst stage and required for normal chromatin architecture. *Molecular and Cellular Biology* **28**, 5093-5105

23. Zhao, X. M., Su, J. M., Wang, F., Liu, D., Ding, J., Yang, Y., Conaway, J. W., Conaway, R. C., Cao, L. L., Wu, D. L., Wu, M., Cai, Y., and Jin, J. J. (2013) Crosstalk between NSL Histone Acetyltransferase and MLL/SET Complexes: NSL Complex Functions in Promoting Histone H3K4 Di-Methylation Activity by MLL/SET Complexes. *Plos Genet* **9**

24. Radzisheuskaya, A., Shliaha, P. V., Grinev, V. V., Shlyueva, D., Damhofer, H., Koche, R., Gorshkov, V., Kovalchuk, S., Zhan, Y. Q., Rodriguez, K. L., Johnstone, A. L., Keogh, M. C., Hendrickson, R. C., Jensen, O. N., and Helin, K. (2021) Complex-dependent histone acetyltransferase activity of KAT8 determines its role in transcription and cellular homeostasis. *Molecular Cell* **81**, 1749-1765

25. Cai, Y., Jin, J., Swanson, S. K., Cole, M. D., Choi, S. H., Florens, L., Washburn, M. P., Conaway, J. W., and Conaway, R. C. (2010) Subunit composition and substrate specificity of a MOF-containing histone acetyltransferase distinct from the male-specific lethal (MSL) complex. *J Biol Chem* **285**, 4268-4272

26. Weber, L. M., Jia, Y. L., Stielow, B., Gisselbrecht, S. S., Cao, Y. H., Ren, Y. P., Rohner, I., King, J., Rothman, E., Fischer, S., Simon, C., Forné, I., Nist, A., Stiewe, T., Bulyk, M. L., Wang, Z. X., and Liefke, R. (2023) The histone acetyltransferase KAT6A is recruited to unmethylated CpG islands via a DNA binding winged helix domain. *Nucleic Acids Research* **51**, 574-594

27. Becht, D. C., Klein, B. J., Kanai, A., Jang, S. M., Cox, K. L., Zhou, B. R., Phanor, S. K., Zhang, Y., Chen, R. W., Ebmeier, C. C., Lachance, C., Galloy, M., Fradet-Turcotte, A., Bulyk, M. L., Bai, Y., Poirier, M. G., Côté, J., Yokoyama, A., and Kutateladze, T. G. (2023) MORF and MOZ acetyltransferases target unmethylated CpG islands through the winged helix domain. *Nat Commun* **14**, 697

28. Ali, M., Yan, K. Z., Lalonde, M. E., Degerny, C., Rothbart, S. B., Strahl, B. D., Côté, J., Yang, X. J., and Kutateladze, T. G. (2012) Tandem PHD Fingers of MORF/MOZ Acetyltransferases Display Selectivity for Acetylated Histone H3 and Are Required for the Association with Chromatin. *J Mol Biol* **424**, 328-338

29. Qiu, Y., Liu, L., Zhao, C., Han, C., Li, F., Zhang, J., Wang, Y., Li, G., Mei, Y., Wu, M., Wu, J., and Shi, Y. (2012) Combinatorial readout of unmodified H3R2 and acetylated H3K14 by the tandem PHD finger of MOZ reveals a regulatory mechanism for HOXA9 transcription. *Genes Dev* **26**, 1376-1391

30. Dreveny, I., Deeves, S. E., Fulton, J., Yue, B., Messmer, M., Bhattacharya, A., Collins, H. M., and Heery, D. M. (2014) The double PHD finger domain of MOZ/MYST3 induces alpha-helical structure of the histone H3 tail to facilitate acetylation and methylation sampling and modification. *Nucleic Acids Res* **42**, 822-835

31. Xiong, X., Panchenko, T., Yang, S., Zhao, S., Yan, P., Zhang, W., Xie, W., Li, Y., Zhao, Y., Allis, C. D., and Li, H. (2016) Selective recognition of histone crotonylation by double PHD fingers of MOZ and DPF2. *Nat Chem Biol* **12**, 1111-1118

32. Klein, B. J., Simithy, J., Wang, X., Ahn, J., Andrews, F. H., Zhang, Y., Côté, J., Shi, X., Garcia, B. A., and Kutateladze, T. G. (2017) Recognition of Histone H3K14 Acylation by MORF. *Structure* **25**, 650-654 e652

33. Holbert, M. A., Sikorski, T., Carten, J., Snowflack, D., Hodawadekar, S., and Marmorstein, R. (2007) The human monocytic leukemia zinc finger histone acetyltransferase domain contains DNA-binding activity implicated in chromatin targeting. *J Biol Chem* **282**, 36603-36613

34. Ullah, M., Pelletier, N., Xiao, L., Zhao, S. P., Wang, K., Degerny, C., Tahmasebi, S., Cayrou, C., Doyon, Y., Goh, S. L., Champagne, N., Côté, J., and Yang, X. J. (2008) Molecular architecture of quartet MOZ/MORF histone acetyltransferase complexes. *Mol Cell Biol* **28**, 6828-6843

35. Yang, X. J. (2015) MOZ and MORF acetyltransferases: Molecular interaction, animal development and human disease. *Biochim Biophys Acta* **1853**, 1818-1826

36. Champagne, N., Bertos, N. R., Pelletier, N., Wang, A. H., Vezmar, M., Yang, Y., Heng, H. H., and Yang, X. J. (1999) Identification of a human histone acetyltransferase related to monocytic leukemia zinc finger protein. *J Biol Chem* **274**, 28528-28536

37. Champagne, N., Pelletier, N., and Yang, X. J. (2001) The monocytic leukemia zinc finger protein MOZ is a histone acetyltransferase. *Oncogene* **20**, 404-409

38. Kitabayashi, I., Aikawa, Y., Nguyen, L. A., Yokoyama, A., and Ohki, M. (2001) Activation of AML1-mediated transcription by MOZ and inhibition by the MOZ-CBP fusion protein. *EMBO J* **20**, 7184-7196

39. Pelletier, N., Champagne, N., Stifani, S., and Yang, X. J. (2002) MOZ and MORF histone acetyltransferases interact with the Runt-domain transcription factor Runx2. *Oncogene* **21**, 2729-2740

40. Klein, B. J., Cox, K. L., Jang, S. M., Côté, J., Poirier, M. G., and Kutateladze, T. G. (2020) Molecular Basis for the PZP Domain of BRPF1 Association with Chromatin. *Structure* **28**, 105-110 e103

41. Zheng, S. P., Bi, Y. C., Chen, H. N., Gong, B., Jia, S. J., and Li, H. T. (2021) Molecular basis for bipartite recognition of histone H3 by the PZP domain of PHF14. *Nucleic Acids Research* **49**, 8961-8973

42. Poplawski, A., Hu, K. F., Lee, W., Natesan, S., Peng, D. N., Carlson, S., Shi, X. B., Balaz, S., Markley, J. L., and Glass, K. C. (2014) Molecular Insights into the Recognition of N-Terminal Histone Modifications by the BRPF1 Bromodomain. *J Mol Biol* **426**, 1661-1676

43. Vezzoli, A., Bonadies, N., Allen, M. D., Freund, S. M., Santiveri, C. M., Kvinlaug, B. T., Huntly, B. J., Gottgens, B., and Bycroft, M. (2010) Molecular basis of histone H3K36me3 recognition by the PWWP domain of BRPF1. *Nat Struct Mol Biol* **17**, 617-619

44. Champagne, K. S., Saksouk, N., Peña, P. V., Johnson, K., Ullah, M., Yang, X. J., Côté, J., and Kutateladze, T. G. (2008) The crystal structure of the ING5 PHD finger in complex with an H3K4me3 histone peptide. *Proteins* **72**, 1371-1376

45. Xu, P., Li, C., Chen, Z., Jiang, S., Fan, S., Wang, J., Dai, J., Zhu, P., and Chen, Z. (2016) The NuA4 Core Complex Acetylates Nucleosomal Histone H4 through a Double Recognition Mechanism. *Mol Cell* **63**, 965-975

46. Weiss, M. S. (2001) Global indicators of X-ray data quality. *J Appl Crystallogr* **34**, 130-135
